# Probing Substituents in the 1- and 3-Position: Tetrahydropyrazino-Annelated Water-Soluble Xanthine Derivatives as Multi-Target Drugs With Potent Adenosine Receptor Antagonistic Activity

**DOI:** 10.3389/fchem.2018.00206

**Published:** 2018-06-26

**Authors:** Pierre Koch, Andreas Brunschweiger, Vigneshwaran Namasivayam, Stefan Ullrich, Annalisa Maruca, Beatrice Lazzaretto, Petra Küppers, Sonja Hinz, Jörg Hockemeyer, Michael Wiese, Jag Heer, Stefano Alcaro, Katarzyna Kiec-Kononowicz, Christa E. Müller

**Affiliations:** ^1^PharmaCenter Bonn, Pharmaceutical Institute, Pharmaceutical Chemistry I, University of Bonn, Bonn, Germany; ^2^Dipartimento di Scienze della Salute, Università degli Studi “Magna Græcia” di Catanzaro, Catanzaro, Italy; ^3^Pharmaceutical Institute, Pharmaceutical Chemistry II, University of Bonn, Bonn, Germany; ^4^UCB Celltech, UCB Pharma S.A., Slough, United Kingdom; ^5^Department of Technology and Biotechnology of Drugs, Faculty of Pharmacy, Jagiellonian University Medical College, Kraków, Poland

**Keywords:** caffeine derivatives, anellated xanthines, tetrahydropyrazino[2, 1-*f*]purinediones, adenosine A_2A_ receptor antagonists, adenosine A_1_ receptor antagonists, monoamine oxidase (MAO) B inhibitors, Alzheimer's disease, Parkinson's disease

## Abstract

Tetrahydropyrazino-annelated theophylline (1,3-dimethylxanthine) derivatives have previously been shown to display increased water-solubility as compared to the parent xanthines due to their basic character. In the present study, we modified this promising scaffold by replacing the 1,3-dimethyl residues by a variety of alkyl groups including combinations of different substituents in both positions. Substituted benzyl or phenethyl residues were attached to the N8 of the resulting 1,3-dialkyl-tetrahydropyrazino[2,1-*f* ]purinediones with the aim to obtain multi-target drugs that block human A_1_ and A_2A_ adenosine receptors (ARs) and monoaminoxidase B (MAO-B). 1,3-Diethyl-substituted derivatives showed high affinity for A_1_ ARs, e.g., **15d** (PSB-18339, 8-*m*-bromobenzyl-substituted) displayed a K_i_ value of 13.6 nM combined with high selectivity. 1-Ethyl-3-propargyl-substituted derivatives exhibited increased A_2A_ AR affinity. The 8-phenethyl derivative **20h** was selective for the A_2A_ AR (K_i_ 149 nM), while the corresponding 8-benzyl-substituted compound **20e** (PSB-1869) blocked A_1_ and A_2A_ ARs with equal potency (K_i_ A_1_, 180 nM; A_2A_, 282 nM). The 1-ethyl-3-methyl-substituted derivative **16a** (PSB-18405) bearing a *m,p*-dichlorobenzyl residue at N8 blocked all three targets, A_1_ ARs (K_i_ 396 nM), A_2A_ ARs (K_i_ 1,620 nM), and MAO-B (IC_50_ 106 nM) with high selectivity vs. the other subtypes (A_2B_ and A_3_ ARs, MAO-A), and can thus be considered as a multi-target drug. Our findings were rationalized by molecular docking studies based on previously published X-ray structures of the protein targets. The new drugs have potential for the treatment of neurodegenerative diseases, in particular Parkinson's disease.

## Introduction

**Figure 1:**
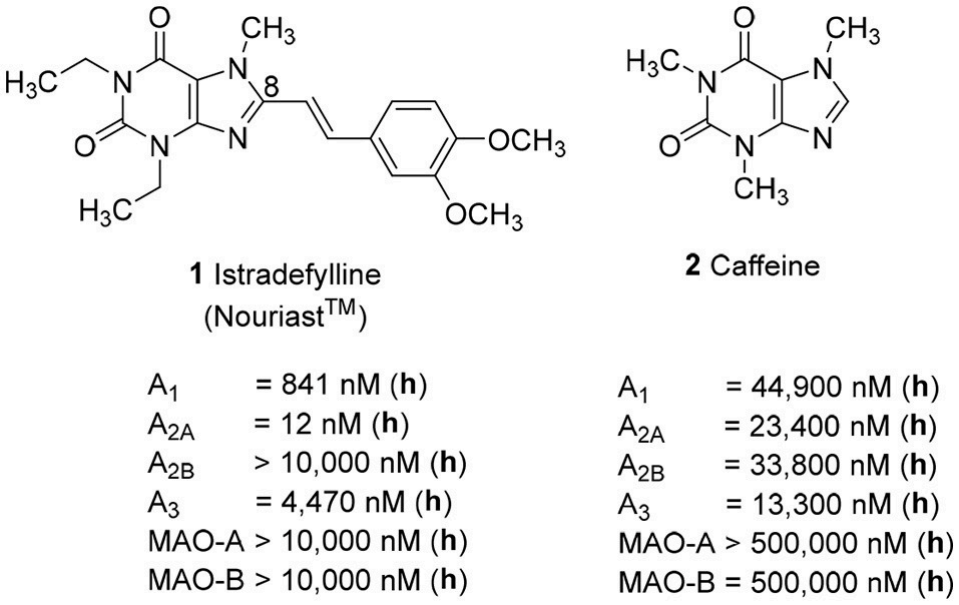
Structures and K_i_/IC_50_ values of the first marketed A_2A_-selective AR antagonist istradefylline (**1**) and the non-selective AR antagonist caffeine (**2**) (h = human; data taken from Petzer et al., 2009; Müller and Jacobson, 2011; Brunschweiger et al., 2014).

Adenosine receptors (ARs), specifically those of the A_2A_ subtype, have emerged as new targets for neurodegenerative diseases, in particular for Parkinson's (PD) and Alzheimer's disease (AD). Several A_2A_-selective AR antagonists have been evaluated in preclinical and clinical trials. The 8-stryrylxanthine derivative istradefylline (Nouriast®, **1**, Figure 1) was approved in Japan as adjunctive treatment of PD in combination with levodopa (Dungo and Deeks, 2013). The consumption of caffeine (**2**), which is a weakly potent and non-selective AR antagonist (Figure 1), was found to protect from PD and AD as demonstrated in a number of animal models as well as in large epidemiological studies in humans (Chen and Chern, 2011; Flaten et al., 2014).

The concept of multi-target drugs interacting simultaneously with two or more pharmacological targets was proposed as a strategy for the treatment of complex diseases such as cancer, psychiatric disorders and neurodegenerative diseases (Geldenhuys and Van Der Schyf, 2013). Multi-target drugs may exhibit high efficacy due to synergistic effects, show a reduced risk of side effects, and result in improved compliance, especially in elderly patients, as compared to combination therapies of two or more different drugs.

In 2009, Petzer et al. suggested that simultaneous targeting of the dopamine-metabolizing, H_2_O_2_-producing enzyme monoamine oxidase B (MAO-B) by inhibitors, and of A_2A_ ARs by antagonists, may be advantageous for the treatment of PD due to their dopamine-enhancing effects. While MAO-B inhibition directly inhibits the degradation of dopamine, A_2A_ AR blockade enhances dopamine-induced D_2_ receptor signaling in the A_2A_-D_2_ heteromeric receptor (Navarro et al., 2018). In addition, A_2A_ AR antagonists reduce cAMP production by blocking A_2A_ AR-induced activation of adenylate cyclase (AC); thus they exert the same intracellular effect as dopamine receptor agonists activating G_i_ protein-coupled receptors, thereby also inhibiting AC. Moreover, both, MAO-B inhibition and A_2A_ AR blockade, are expected to show additional neuroprotective activities, MAO-B inhibitors by reducing hydrogen peroxide production, and A_2A_ AR antagonists by various mechanisms (Fišar, 2016; Xu et al., 2016). Therefore, such a dual target-directed approach may result in synergistic or at least additive effects thereby possibly halting or reducing the devastating progression of neurodegenerative diseases.

**Figure 2:**
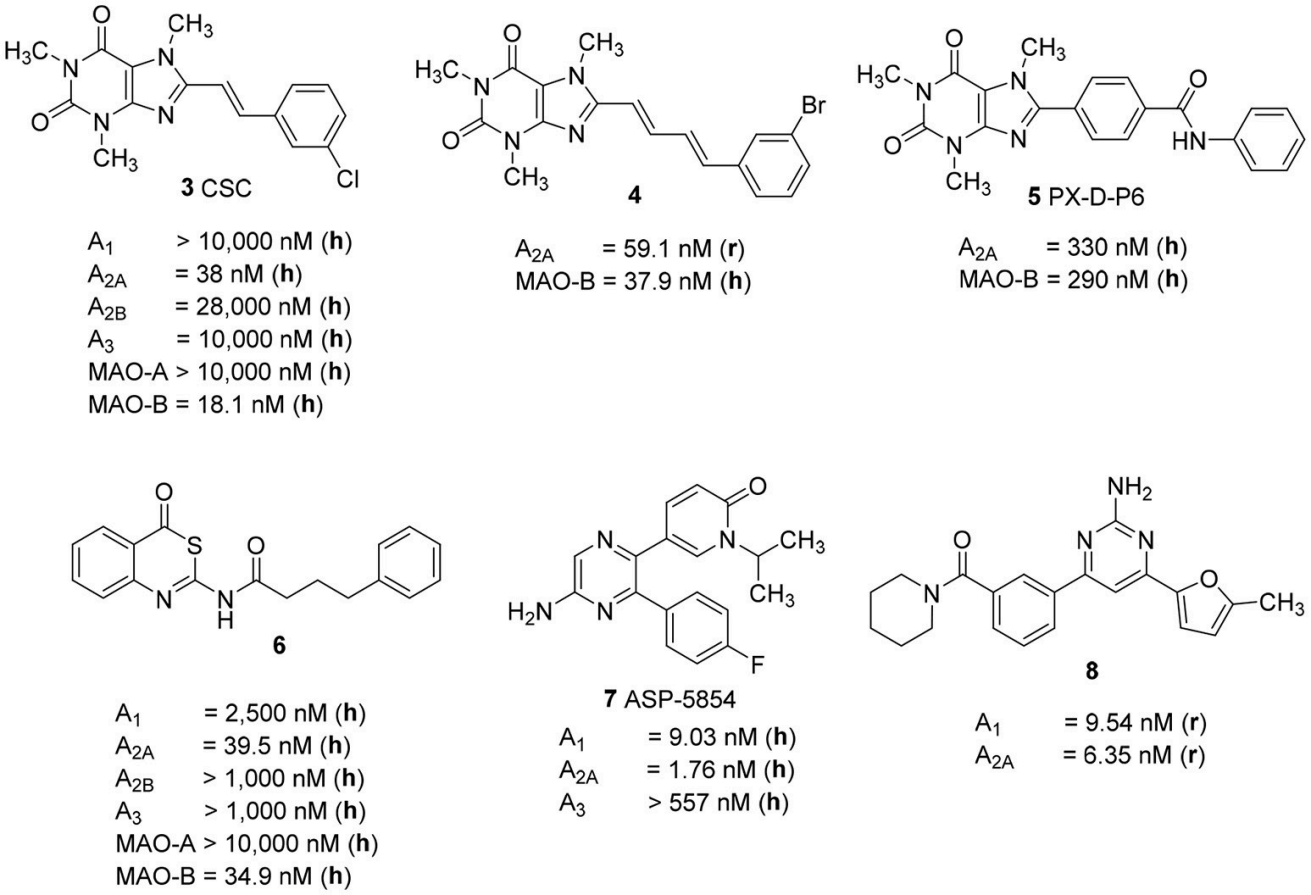
Structures and K_i_/IC_50_ values of dual A_2A_ AR antagonists/MAO-B inhibitors and dual A_1_/A_2A_ AR antagonists (r, rat; h, human).

Several studies focused on the design of caffeine derivatives that display A_2A_ AR antagonistic as well as MAO-B inhibitory activity have been published (Petzer and Petzer, 2015). 8-*m*-Chlorostyrylcaffeine (CSC, **3**) was the first reported example of an A_2A_ AR antagonist that also showed high MAO-B inhibitory activity (Figure 2; Chen et al., 2002). Petzer and coworkers reported on a series of (*E*,*E*)-8-(4-phenylbutadien-1-yl)xanthines, among which caffeine derivative **4** showed potent A_2A_ AR/MAO-B inhibitory activity (K_i_ rat A_2A_ AR: 59.1 nM; IC_50_ human MAO-B: 37.9 nM) (Pretorius et al., 2008). Recently, Wang *et al*. published another series of xanthine-based dual A_2A_ AR antagonists/MAO-B inhibitors. The most potent example of this series was PX-D-P6 (**5**) (K_i_ human A_2A_ AR: 330 nM; IC_50_ human MAO-B: 260 nM), which showed anti-cataleptic effects in a haloperidol model in rat (Wang et al., 2017). The first non-xanthine-derived dual A_2A_ AR antagonists/MAO-B inhibitors were reported by our group: *N*-(4-oxo-4*H*-3,1-benzothiazin-2-yl)-4-phenylbutanamide (**6**, Figure 2) was the most potent compound in that series of benzothiazines displaying a K_i_ value of 39.5 nM at the human A_2A_ AR, and an IC_50_ value of 34.9 nM at human MAO-B combined with excellent selectivity vs. other AR subtypes as well as vs. MAO-A (Stössel et al., 2013).

As a further dual-target drug approach for the treatment of PD, the combination of A_2A_ and A_1_ AR blockade was suggested. The dual A_1_/A_2A_ AR antagonist ASP-5854 (**7**) (K_i_ human A_1_ 9.03 nM; K_i_ human A_2A_ 1.76 nM) was extensively characterized in several animal models of PD, as well as for its effects on cognition. Compound **7** reversed haloperidol-induced catalepsy in monkeys. Moreover, it produced positive results in rats in the passive avoidance test, a model of cognition, in which the A_2A_-selective antagonist istradefylline (**1**) had been inactive (Mihara et al., 2007). The aminopyrimidine-based dual A_1_/A_2A_ AR antagonist **8**, which displays high affinity for both AR subtypes (K_i_ rat A_1_ 6.34 nM; K_i_ rat A_2A_ 9.54 nM), showed *in vivo* efficacy in a rat model of haloperidol-induced catalepsy (Robinson et al., 2015). These results support the hypothesis that a dual A_1_/A_2A_ AR antagonist may provide additional benefit to PD patients as compared to antagonists that selectively block A_2A_ ARs, due to their positive effects on cognitive impairment often associated with the disease.

**Figure 3:**
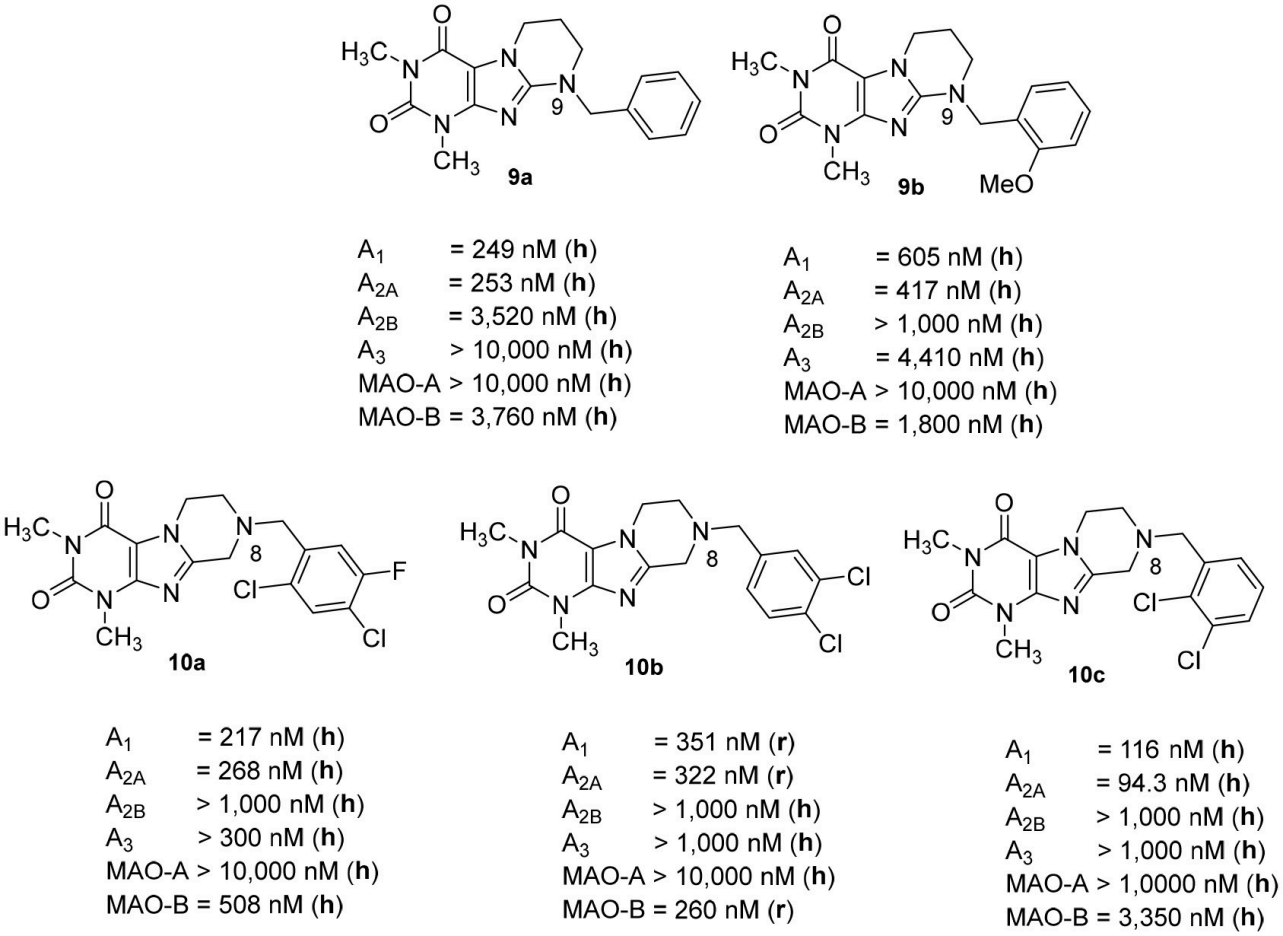
Structures and K_i_/IC_50_ values of tetrahydropyrimido[2,1-*f* ]purinediones (**9a**,**b**) and tetrahydropyrazino[2,1-*f* ]purinediones (**10a**,**b**,**c**) as dual- and multi-target drugs (r, rat; h, human).

We previously reported on the development of tetrahydropyrimido[2,1-*f* ]purinediones (e.g., compounds **9a**, **9b**) as AR antagonists and MAO-B inhibitors (Figure 3; Drabczynska et al., 2007; Koch et al., 2013). This class of compounds can be envisaged as tricyclic caffeine derivatives. They represent analogs of 8-styrylxanthines, that are sterically constrained by anellation of a tetrahydropyrimidine ring to the 7,8-position of xanthine mimicking the (*E)*-configurated styryl sub-structure of CSC (**6**). Compound **9a** is a potent dual A_1_/A_2A_ AR antagonist (K_i_, human receptors, A_1_: 249 nM, A_2A_: 253 nM), while compound **9b** is a moderately potent triple-target A_1_/A_2A_ AR antagonist/MAO-B inhibitor with K_i_-/IC_50_-values of 605, 417, and 1,800 nM, respectively. However, a major drawback of this class of compounds is their low water-solubility, similar to that of many xanthines such as **3**. In continuation of our efforts to develop improved, more water-soluble A_2A_ AR antagonists, structures **10** had been designed (Figure 3; Brunschweiger et al., 2014, 2016). In **10**, the nitrogen atom in position 9 of the tricyclic structures **9** was (formally) shifted to position 8. Consequently, the nitrogen atom is much more basic, and compounds **10** display improved water-solubility at physiological pH values. Several compounds of this series showed triple-target inhibition, one of the best derivatives being 8-(2,4-dichloro-5-fluorobenzyl)-1,3-dimethyl-6,7,8,9-tetrahydropyrazino[2,1-*f* ]purine-2,4(*1H,3H*)-dione (**10a**, human receptors: K_i_ A_1_: 217 nM, K_i_ A_2A_: 268 nM, IC_50_ human MAO-B: 508 nM). 8-(3,4-Dichlorobenzyl)-1,3-dimethyl-6,7,8,9-tetrahydropyrazino[2,1-*f* ]purine-2,4(1*H*,3*H*)-dione (**10b**) was the best triple-target drug in rat (K_i_ rat receptors A_1_: 351 nM, A_2A_: 322 nM, IC_50_ rat MAO-B: 260 nM) and should therefore be a suitable tool for animal studies. 1,3-Dimethyl-6,7,8,9-tetrahydropyrazino[2,1-*f* ]purine-2,4(1*H*,3*H*)-dione (**10c**, K_i_, human receptors, A_1_: 116 nM, A_2A_: 94 nM) was identified as a potent dual A_1_/A_2A_R antagonist.

**Figure 4:**
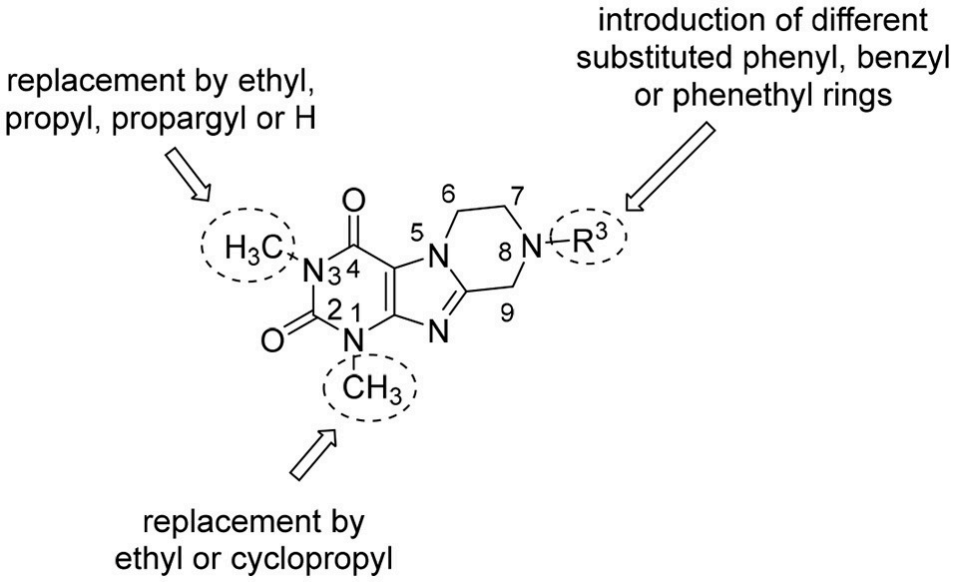
Structural modifications introduced into new tetrahydropyrazino[2,1-*f* ]purinedione derivatives.

In the present study, we report on the synthesis of a series of 64 novel tetrahydropyrazino[2,1-*f* ]purinedione derivatives **11**–**20**. The final products **13**–**20** were evaluated as antagonists at all four AR subtypes (A_1_, A_2A_, A_2B_, A_3_) and as inhibitors of both MAO isoenzymes (MAO-A and MAO-B). Substituents on positions N1, N3 and N8 were varied in order to modulate the biological activities of the compounds (Figure 4). Differently substituted benzyl and phenethyl residues were introduced at position 8 keeping the nitrogen atom N8 basic to allow for protonation. In order to study the effects of substituents at nitrogen atoms N1 and N3 on the biological activity of the compounds, methyl groups found in caffeine derivatives and in the majority of published tetrahydropyrazino[2,1-*f* ]purinediones were replaced by ethyl, propyl, cyclopropyl, or propargyl (prop-2-yn-1-yl) moieties, or remained unsubstituted. Within the series of 1-ethyl-3-propargyl-tetrahydropyrazino[2,1-*f* ]purine-2,4(1*H*,3*H*)-diones **20** phenyl residues bearing different substituents were additionally introduced in position 8 for comparison with benzyl- and phenethyl-substituted derivatives.

## Materials and methods

### General information

All commercially available reagents and solvents were used without further purification. The reactions were monitored by thin layer chromatography (TLC) using aluminum sheets coated with silica gel 60 F_254_ (Merck). Melting points were determined on a Büchi 530 melting point apparatus and are uncorrected. Column chromatography was carried out on silica gel 0.040–0.063 mm using a Sepacore flash chromatography system (Büchi). ^1^H NMR and ^13^C NMR data were recorded on a Bruker Avance spectrometer at 500 MHz for proton and 125 MHz for carbon at ambient temperature (for ^13^C NMR data, see Supplementary Materials). Shifts are given in ppm relative to the remaining protons of the deuterated solvents. The purity of the tested compounds was determined by HPLC-UV obtained on an LC-MS instrument (Applied Biosystems API 2000 LC-MS/MS, HPLC Agilent 1100) using the procedure as follows: dissolving of the compounds at a concentration of 1.0 mg/mL in methanol and if necessary sonication to complete dissolution. Then, 10 μL of the substance solution was injected into a Phenomenex Luna C18 HPLC column (50 × 2.00 mm, particle size 3 μm) and elution was performed for 30 min at a flow rate of 250 μL/min with a gradient of water: methanol either containing 2 mM ammonium acetate from 90:10 up to 0:100, starting the gradient after 10 min (system A) or containing 2 mM ammonium acetate and 0.1% formic acid from 90:10 up to 0:100, starting the gradient after 10 min (system B). UV absorption was detected from 220 to 400 nm using a diode array detector. Mass spectra were recorded on an API 2000 mass spectrometer (electron spray ion source, Applied Biosystems, Darmstadt, Germany) coupled with an Agilent 1100 HPLC system.

### Synthesis of final compounds

#### General procedure for the preparation of 8-substituted 6,7,8,9-tetrahydropyrazino[2,1-*f*]purine-2,4(1*H*,3*H*)-diones 13–15 (general procedure A)

7-(2-Bromoethyl)-8-hydroxymethylpurine-2,4-dione **23c**, **23d** or **23e** (400 mg) was dissolved in dry CH_2_Cl_2_ (50 mL). The solution was cooled to 0°C and PBr_3_ (0.4 mL) was added dropwise. The reaction mixture was allowed to warm to rt and stirred for 1 h. Then it was cooled to 0°C again. To hydrolyze the excess of PBr_3_, saturated aq. NaHCO_3_-solution (5 mL) was added and the pH was set to 7–8 by addition of NaHCO_3_. Then, the lower layer was separated in a separating funnel and the aqueous layer was extracted with CH_2_Cl_2_ (2 × 50 mL). The organic extracts were combined, dried over Na_2_SO_4_ and the solvent was removed by rotary evaporation. The residue was dissolved in a mixture of dimethoxyethane (10 mL) and DIPEA (0.5 mL). To effect ring closing reaction, an appropriate amine was added and the solution was stirred overnight at rt. The volatiles were removed by rotary evaporation and the product precipitated upon addition of H_2_O (20 mL). For purification, the compound was either filtered off and washed with H_2_O (3 × 5 mL) and diethylether (3 × 10 mL) or subjected to flash-chromatography (silica gel, CH_2_Cl_2_:MeOH 1:0 to 40:1).

#### 8-(2-bromobenzyl)-1-methyl-6,7,8,9-tetrahydropyrazino[2,1-*f*]purine-2,4(1*H*,3*H*)-dione (13a)

General procedure A. Yield: 83%; mp: 289°C; ^1^H-NMR (CDCl_3_) δ 7.55 (dd, ^3^*J* = 8.20 Hz, ^4^*J* = 1.25 Hz, 1H, H-3, phenyl), 7.44 (d, ^3^*J* = 7.25 Hz, 1H, H-6, phenyl), 7.28 (ddd, ^3^*J* = 7.60 Hz, ^3^*J* = 8.55 Hz, ^4^*J* = 1.60 Hz, 1H, H-4, phenyl), 7.14 (dd, ^3^*J* = 7.55 Hz, ^3^*J* = 7.55 Hz, 1H, H-5, phenyl), 4.35 (t, ^3^*J* = 5.35 Hz, 2H, 2 × H-6), 3.86 (s, 2H, N8-CH_2_), 3.83 (s, 2H, 2 × H-9), 3.51 (s, 3H, N1-CH_3_), 3.02 (t, ^3^*J* = 5.40 Hz, 2H, 2 × H-7). ESI-MS: positive mode 390.3 and 392.3 [M+H]^+^. HPLC: 99.9% (A) and 99.6% (B).

#### 1-methyl-8-(3-(trifluoromethyl)benzyl)-6,7,8,9-tetrahydropyrazino[2,1-*f*]purine-2,4(1*H*,3*H*)-dione (13b)

General procedure A. Yield: 65%; mp: 215°C; ^1^H-NMR (CDCl_3_) δ 9.41 (s, 1H, N3-H), 7.59 (br s, 1H, H-2, phenyl), 7.53–7.51 (m, 2H, H-5 and H-6, phenyl), 7.45–7.42 (m, 1H, H-4, phenyl), 4.32 (t, ^3^*J* = 5.35 Hz, 2H, 2 × H-6), 3.77 (s, 2H, N8-CH_2_), 3.73 (s, 2H, 2 × H-9), 3.45 (s, 3H, N1-CH_3_), 2.94 (t, ^3^*J* = 5.35 Hz, 2H, 2 × H-7). ESI-MS: positive mode 380.4 [M+H]^+^. HPLC: 99.4% (A) and 99.9% (B).

#### 8-(3-chlorophenethyl)-1-methyl-6,7,8,9-tetrahydropyrazino[2,1-*f*]purine-2,4(1*H*,3*H*)-dione (13c)

General procedure A. Yield: 62%; mp: 228°C; ^1^H-NMR (CDCl_3_) δ 8.11 (s, 1H, N3-H), 7.21–7.18 (m, 3H, H-2, H-4 and H-5, phenyl), 7.09–7.07 (m, 2H, H-6, phenyl), 4.30 (t, ^3^*J* = 5.35 Hz, 2H, 2 × H-6), 3.81 (s, 2H, 2 × H-9), 3.53 (s, 3H, N1-CH_3_), 2.96 (t, ^3^*J* = 5.35 Hz, 2H, 2 × H-7), 2.83 (s, 4H, N8-CH_2_-CH_2_). ESI-MS: negative mode 358.0 [M-H]^−^, positive mode 360.3 [M+H]^+^. HPLC: 98.8% (A) and 98.9% (B).

#### 8-(3-bromophenethyl)-1-methyl-6,7,8,9-tetrahydropyrazino[2,1-*f*]purine-2,4(1*H*,3*H*)-dione (13d)

General procedure A. Yield: 71%; mp: 240°C; ^1^H-NMR (CDCl_3_) δ 8.09 (s, 1H, N3-H), 7.35–7.33 (m, 2H, H-2 and H-5, phenyl), 7.17–7.11 (m, 2H, H-4 and H-6, phenyl), 4.30 (t, ^3^*J* = 5.35 Hz, 2H, 2 × H-6), 3.81 (s, 2H, 2 × H-9), 3.53 (s, 3H, N1-CH_3_), 2.97 (br s, 2H, 2 × H-7), 2.84 (br s, 4H, N8-CH_2_-CH_2_). ESI-MS: negative mode 404.0 [M-H]^−^, positive mode 406.4 [M+H]^+^. HPLC: 97.5% (A) and 97.5% (B).

#### 1-methyl-8-(3-(trifluoromethyl)phenethyl)-6,7,8,9-tetrahydropyrazino[2,1-*f*]purine-2,4(1*H*,3*H*)-dione (13e)

General procedure A. Yield: 63%; mp: 252°C; ^1^H-NMR (CDCl_3_) δ 8.10 (s, 1H, N3-H), 7.46 (d, ^4^*J* = 1.60 Hz, 1H, H-2, phenyl), 7.46–7.44 (m, 1H, H-5, phenyl), 7.38–7.35 (m, 2H, H-4 and H-6, phenyl), 4.33 (t, ^3^*J* = 5.35 Hz, 2H, 2 × H-6), 3.83 (s, 2H, 2 × H-9), 3.53 (s, 3H, N1-CH_3_), 2.98 (t, ^3^*J* = 5.35 Hz, 2H, 2 × H-7), 2.92–2.84 (m, 4H, N8-CH_2_-CH_2_). ESI-MS: negative mode 392.0 [M-H]^−^, positive mode 394.4 [M+H]^+^. HPLC: 97.7% (A) and 97.6% (B).

#### 8-(2,4-dichlorophenethyl)-1-methyl-6,7,8,9-tetrahydropyrazino[2,1-*f*]purine-2,4(1*H*,3*H*)-dione (13f)

General procedure A. Yield: 55%; mp: 260°C; ^1^H-NMR (CDCl_3_) δ 7.35 (s, 1H, H-3, phenyl), 7.17 (d, ^3^*J* = 7.90 Hz, 2H, H-5 and H-6, phenyl), 4.34 (br s, 2H, 2 × H-6), 3.87 (s, 2H, 2 × H-9), 3.53 (s, 3H, N1-CH_3_), 3.02 (br s, 2H, 2 × H-7), 2.97 (t, ^3^*J* = 6.90 Hz, 2H, N8-CH_2_), 2.84 (t, ^3^*J* = 7.25 Hz, 2H, N8-CH_2_-*CH*_2_). ESI-MS: negative mode 392.0 [M-H]^−^, positive mode 394.4 [M+H]^+^. HPLC: 95.0% (A) and 95.2% (B).

#### 8-(3,4-dichlorophenethyl)-1-methyl-6,7,8,9-tetrahydropyrazino[2,1-*f*]purine-2,4(1*H*,3*H*)-dione (13g)

General procedure A. Yield: 69%; mp: 241°C; ^1^H-NMR (CDCl_3_) δ 7.33 (d, ^3^*J* = 8.15 Hz, 1H, H-5, phenyl), 7.29 (d, ^4^*J* = 1.90 Hz, 1H, H-2, phenyl), 7.02 (dd, ^3^*J* = 8.15 Hz, ^4^*J* = 2.25 Hz, 1H, H-6, phenyl), 4.34 (t, ^3^*J* = 5.05 Hz, 2H, 2 × H-6), 3.81 (s, 2H, 2 × H-9), 3.54 (s, 3H, N1-CH_3_), 2.97 (t, ^3^*J* = 5.05 Hz, 2H, 2 × H-7), 2.82 (br s, 4H, N8-CH_2_-CH_2_). ESI-MS: negative mode 392.0 [M-H]^−^, positive mode 394.3 [M+H]^+^. HPLC: 99.9% (A) and 99.1% (B).

#### 8-(3,4-dichlorophenethyl)-1-methyl-6,7,8,9-tetrahydropyrazino[2,1-*f*]purine-2,4(1*H*,3*H*)-dione (13h)

General procedure A. Yield: 56%; mp: 276°C; ^1^H-NMR (DMSO-*d*_6_) δ 11.01 (s, 1H, N1-H), 7.63 (d, ^4^*J* = 1.9 Hz, 1H, H-2, phenyl), 7.61 (d, ^4^*J* = 8.2 Hz, 1H, H-4, phenyl), 7.37 (dd, ^3^*J* = 8.2 Hz, ^4^*J* = 1.9Hz, 1H, H-5, phenyl), 4.18 (t, *J* = 5.4 Hz, 2H, 2 × H-6), 3.75 (s, 2H, N8-CH_2_), 3.73 (s, 2H, 2 × H-9), 3.31 (s, 3H, N1-CH_3_), 2.92 (t, 2H, 2 × H-7). ESI-MS: negative mode 378.3 [M-H]^−^, positive mode 380.1 [M+H]^+^. HPLC: 99.2% (C).

#### 8-(2-chloro-5-(trifluoromethyl)benzyl)-1-methyl-6,7,8,9-tetrahydropyrazino[2,1-*f*]purine-2,4(1*H*,3*H*)-dione (13i)

General procedure A. Yield: 71%; mp: 253°C; ^1^H-NMR (CDCl_3_) δ 7.74 (s, 1H, H-6, phenyl), 7.49 (2 × d, ^3^*J* = 8.50 Hz, 2H, H-3 and H-4, phenyl), 4.35 (t, ^3^*J* = 5.35 Hz, 2H, 2 × H-6), 3.89 (s, 2H, N8-CH_2_), 3.82 (s, 2H, 2 × H-9), 3.53 (s, 3H, N1-CH_3_), 3.02 (t, ^3^*J* = 5.35 Hz, 2H, 2 × H-7). ESI-MS: positive mode 414.3 [M+H]^+^. HPLC: 97.1% (A) and 97.4% (B).

#### 8-(2-bromobenzyl)-3-ethyl-1-methyl-6,7,8,9-tetrahydropyrazino[2,1-*f*]purine-2,4(1*H*,3*H*)-dione (14a)

General Procedure A. Yield: 75%; mp: 224°C; ^1^H-NMR (CDCl_3_) δ 7.55 (dd, ^3^*J* = 8.20 Hz, ^4^*J* = 1.25 Hz, 1H, H-3, phenyl), 7.44 (d, ^3^*J* = 7.65 Hz, ^4^*J* = 1.65 Hz, 1H, H-6, phenyl), 7.30–7.27 (m, 1H, H-4, phenyl), 7.16–7.13 (m, 1H, H-5, phenyl), 4.35 (t, ^3^*J* = 5.35 Hz, 2H, 2 × H-6), 4.04 (q, ^3^*J* = 6.90 Hz, 2H, N3-CH_2_), 3.86 (s, 2H, N8-CH_2_), 3.83 (s, 2H, 2 × H-9), 3.51 (s, 3H, N1-CH_3_), 3.02 (t, ^3^*J* = 5.40 Hz, 2H, 2 × H-7), 1.21 (t, ^3^*J* = 7.25 Hz, 3H, N3-CH_2_-*CH*_3_). ESI-MS: positive mode 418.3 and 420.3 [M+H]^+^. HPLC: 98.4% (A) and 99.0% (B).

#### 8-(3-bromobenzyl)-3-ethyl-1-methyl-6,7,8,9-tetrahydropyrazino[2,1-*f*]purine-2,4(1*H*,3*H*)-dione (14b)

General Procedure A. Yield: 81%; mp: 194°C; ^1^H-NMR (CDCl_3_) δ 7.50 (s, 1H, H-2, phenyl), 7.42–7.40 (m, 1H, H-4, phenyl), 7.26–7.23 (m, 1H, H-6, phenyl), 7.21–7.18 (m, 1H, H-5, phenyl), 4.55 (t, ^3^*J* = 5.35 Hz, 2H, 2 × H-6), 4.04 (q, ^3^*J* = 6.90 Hz, 2H, N3-CH_2_), 3.93 (br s, 4H, N8-CH_2_, 2 × H-9), 3.52 (s, 3H, N1-CH_3_), 3.18 (t, ^3^*J* = 5.05 Hz, 2H, 2 × H-7), 1.21 (t, ^3^*J* = 7.25 Hz, 3H, N3-CH_2_-*CH*_3_). ESI-MS: positive mode 418.3 and 420.3 [M+H]^+^. HPLC: 99.7% (A) and 99.9% (B).

#### 8-(4-bromobenzyl)-3-ethyl-1-methyl-6,7,8,9-tetrahydropyrazino[2,1-*f*]purine-2,4(1*H*,3*H*)-dione (14c)

General Procedure A. Yield: 42%; mp: 156°C; ^1^H-NMR (CDCl_3_) δ 7.45 (d, ^3^*J* = 8.20 Hz, 2H, H-3 and H-5, phenyl), 7.44 (d, ^3^*J* = 8.20 Hz, 2H, H-2 and H-6, phenyl), 4.32 (t, ^3^*J* = 5.35 Hz, 2H, 2 × H-6), 4.04 (q, ^3^*J* = 6.90 Hz, 2H, N3-CH_2_), 3.72 (s, 2H, N8-CH_2_), 3.67 (s, 2H, 2 × H-9), 3.51 (s, 3H, N1-CH_3_), 2.92 (t, ^3^*J* = 5.35 Hz, 2H, 2 × H-7), 1.21 (t, ^3^*J* = 7.25 Hz, 3H, N3-CH_2_-*CH*_3_). ESI-MS: positive mode 418.3 and 420.3 [M+H]^+^. HPLC: 98.7% (A) and 99.5% (B).

#### 3-ethyl-1-methyl-8-(2-(trifluoromethyl)benzyl)-6,7,8,9-tetrahydropyrazino[2,1-*f*]purine-2,4(1*H*,3*H*)-dione (14d)

General Procedure A. Yield: 60%; mp: 182°C; ^1^H-NMR (CDCl_3_) δ 7.72 (d, ^3^*J* = 7.75 Hz, 1H, H-6, phenyl), 7.66 (d, ^3^*J* = 7.80 Hz, 1H, H-3, phenyl), 7.53 (dd, ^3^*J* = 7.60 Hz, ^3^*J* = 7.20 Hz, 1H, H-5, phenyl), 7.39 (dd, ^3^*J* = 7.60 Hz, ^3^*J* = 7.65 Hz, 1H, H-4, phenyl), 4.33 (t, ^3^*J* = 5.35 Hz, 2H, 2 × H-6), 4.04 (q, ^3^*J* = 6.90 Hz, 2H, N3-CH_2_), 3.90 (s, 2H, N8-CH_2_), 3.78 (s, 2H, 2 × H-9), 3.51 (s, 3H, N1-CH_3_), 2.97 (t, ^3^*J* = 5.85 Hz, 2H, 2 × H-7), 1.21 (t, ^3^*J* = 7.25 Hz, 3H, N3-CH_2_-*CH*_3_). ESI-MS: positive mode 408.1 [M+H]^+^. HPLC: 99.9% (A) and 99.0% (B).

#### 3-ethyl-1-methyl-8-(3-(trifluoromethyl)benzyl)-6,7,8,9-tetrahydropyrazino[2,1-*f*]purine-2,4(1*H*,3*H*)-dione (14e)

General Procedure A. Yield: 65%; mp: 198°C; ^1^H-NMR (CDCl_3_) δ 7.60 (s, 1H, H-2, phenyl), 7.58–7.52 (m, 1H, H-5 and H-6, phenyl), 7.47–7.44 (m, 1H, H-4, phenyl), 4.47 (t, ^3^*J* = 5.35 Hz, 2H, 2 × H-6), 4.04 (q, ^3^*J* = 6.90 Hz, 2H, N3-CH_2_), 4.03 (s, 2H, N8-CH_2_), 3.84 (s, 2H, 2 × H-9), 3.51 (s, 3H, N1-CH_3_), 3.08 (t, ^3^*J* = 5.35 Hz, 2H, 2 × H-7), 1.21 (t, ^3^*J* = 7.25 Hz, 3H, N3-CH_2_-*CH*_3_). ESI-MS: positive mode 408.4 [M+H]^+^. HPLC: 98.4% (A) and 99.6% (B).

#### 3-ethyl-1-methyl-8-(4-(trifluoromethyl)benzyl)-6,7,8,9-tetrahydropyrazino[2,1-*f*]purine-2,4(1*H*,3*H*)-dione (14f)

General Procedure A. Yield: 44%; mp: 162°C; ^1^H-NMR (CDCl_3_) δ 8.00 (d, ^3^*J* = 8.50 Hz, 2H, H-3 and H-5, phenyl), 7.64 (d, ^3^*J* = 8.50 Hz, 2H, H-2 and H-6, phenyl), 4.35 (t, ^3^*J* = 5.35 Hz, 2H, 2 × H-6), 4.04 (q, ^3^*J* = 6.90 Hz, 2H, N3-CH_2_), 3.96 (s, 2H, N8-CH_2_), 3.87 (s, 2H, 2 × H-9), 3.48 (s, 3H, N1-CH_3_), 3.05 (t, ^3^*J* = 5.40 Hz, 2H, 2 × H-7), 1.21 (t, ^3^*J* = 7.25 Hz, 3H, N3-CH_2_-*CH*_3_). ESI-MS: positive mode 408.3 [M+H]^+^. HPLC: 99.9% (A) and 99.0% (B).

#### 3-ethyl-8-(3-fluorobenzyl)-1-methyl-6,7,8,9-tetrahydropyrazino[2,1-*f*]purine-2,4(1*H*,3*H*)-dione (14g)

General Procedure A. Yield: 54%; mp: 154°C; ^1^H-NMR (CDCl_3_) δ 7.31–7.27 (m, 1H, H-5, phenyl), 7.10 (d, ^3^*J* = 7.60 Hz, 1H, H-2, phenyl), 7.08–7.05 (m, 1H, H-6, phenyl), 7.00–6.96 (m, 1H, H-4, phenyl), 4.33 (t, ^3^*J* = 5.35 Hz, 2H, 2 × H-6), 4.04 (q, ^3^*J* = 6.90 Hz, 2H, N3-CH_2_), 3.71 (br s, 4H, N8-CH_2_ and 2 × H-9), 3.51 (s, 3H, N1-CH_3_), 2.93 (t, ^3^*J* = 5.70 Hz, 2H, 2 × H-7), 1.21 (t, ^3^*J* = 7.25 Hz, 3H, N3-CH_2_-*CH*_3_). ESI-MS: positive mode 358.3 [M+H]^+^. HPLC: 98.2% (A) and 99.5% (B).

#### 3-ethyl-8-(4-fluorobenzyl)-1-methyl-6,7,8,9-tetrahydropyrazino[2,1-*f*]purine-2,4(1*H*,3*H*)-dione (14h)

General Procedure A. Yield: 33%; mp: 158°C; ^1^H-NMR (CDCl_3_) δ 7.30–7.28 (m, 2H, H-2 and H-6, phenyl), 7.03–7.00 (m, 2H, H-3 and H-5, phenyl), 4.33 (t, ^3^*J* = 5.35 Hz, 2H, 2 × H-6), 4.04 (q, ^3^*J* = 6.90 Hz, 2H, N3-CH_2_), 3.72 (s, 2H, N8-CH_2_), 3.71 (s, 2H, 2 × H-9), 3.51 (s, 3H, N1-CH_3_), 2.94 (t, ^3^*J* = 5.45 Hz, 2H, 2 × H-7), 1.21 (t, ^3^*J* = 7.25 Hz, 3H, N3-CH_2_-*CH*_3_). ESI-MS: positive mode 358.3 [M+H]^+^. HPLC: 99.2% (A) and 98.4% (B).

#### 8-(3-chlorobenzyl)-3-ethyl-1-methyl-6,7,8,9-tetrahydropyrazino[2,1-*f*]purine-2,4(1*H*,3*H*)-dione (14i)

General Procedure A. Yield: 74%; mp: 202°C; ^1^H-NMR (CDCl_3_) δ 7.34 (d, ^4^*J* = 1.25 Hz, 1H, H-2, phenyl), 7.27–7.26 (m, 2H, H-4 and H-6, phenyl), 7.21–7.19 (m, 1H, H-5, phenyl), 4.81 (t, ^3^*J* = 5.00 Hz, 2H, 2 × H-6), 4.04 (q, ^3^*J* = 6.90 Hz, 2H, N3-CH_2_), 3.83 (br s, 4H, N8-CH_2_, 2 × H-9), 3.53 (s, 3H, N1-CH_3_), 3.38 (t, ^3^*J* = 5.35 Hz, 2H, 2 × H-7), 1.21 (t, ^3^*J* = 7.25 Hz, 3H, N3-CH_2_-*CH*_3_). ESI-MS: positive mode 374.3 [M+H]^+^. HPLC: 99.9% (A) and 99.9% (B).

#### 8-(2,5-dichlorobenzyl)-3-ethyl-1-dimethyl-6,7,8,9-tetrahydropyrazino[2,1-*f*]purine-2,4(1*H*,3*H*)-dione (14j)

General Procedure A. Yield: 80%; mp: 165°C; ^1^H-NMR (CDCl_3_) δ 7.44 (d, ^4^*J* = 2.50 Hz, 1H, H-6, phenyl), 7.30 (d, ^3^*J* = 8.50 Hz, 1H, H-3, phenyl), 7.20 (dd, ^3^*J* = 8.50 Hz, ^4^*J* = 2.50 Hz, 1H, H-4, phenyl), 4.36 (t, ^3^*J* = 5.35 Hz, 2H, 2 × H-6), 4.04 (q, ^3^*J* = 6.90 Hz, 2H, N3-CH_2_), 3.86 (s, 2H, N8-CH_2_), 3.84 (s, 2H, 2 × H-9), 3.52 (s, 3H, N1-CH_3_), 3.04 (t, ^3^*J* = 5.35 Hz, 2H, 2 × H-7), 1.21 (t, ^3^*J* = 7.25 Hz, 3H, N3-CH_2_-*CH*_3_). ESI-MS: positive mode 409.3 [M+H]^+^. HPLC: 98.6% (A) and 99.1% (B).

#### 8-(2,6-dichlorobenzyl)-3-ethyl-1-methyl-6,7,8,9-tetrahydropyrazino[2,1-*f*]purine-2,4(1*H*,3*H*)-dione (14k)

General Procedure A. Yield: 64%; mp: 210°C; ^1^H-NMR (CDCl_3_) δ 7.33 (d, ^3^*J* = 8.40 Hz, 2H, H-3 and H-5, phenyl), 7.18 (dd, ^3^*J* = 8.40 Hz, 1H, H-4, phenyl), 4.38 (t, ^3^*J* = 5.35 Hz, 2H, 2 × H-6), 4.04 (q, ^3^*J* = 6.90 Hz, 2H, N3-CH_2_), 3.91 (s, 2H, N8-CH_2_), 3.83 (s, 2H, 2 × H-9), 3.53 (s, 3H, N1-CH_3_), 3.02 (t, ^3^*J* = 5.35 Hz, 2H, 2 × H-7), 1.21 (t, ^3^*J* = 7.25 Hz, 3H, N3-CH_2_-*CH*_3_). ESI-MS: positive mode 394.1 [M+H]^+^. HPLC: 99.0% (A) and 99.0% (B).

#### 3-ethyl-8-(2-fluoro-3-(trifluoromethyl)benzyl)-1-methyl-6,7,8,9-tetrahydropyrazino[2,1-*f*]purine-2,4(1*H*,3*H*)-dione (14l)

General Procedure A. Yield: 42%; mp: 178°C; ^1^H-NMR (CDCl_3_) δ 7.63–7.60 (m, 1H, H-4, phenyl), 7.57–7.54 (m, 1H, H-5, phenyl), 7.25–7.22 (m, 1H, H-6, phenyl), 4.45 (t, ^3^*J* = 5.05 Hz, 2H, 2 × H-6), 4.04 (q, ^3^*J* = 6.90 Hz, 2H, N3-CH_2_), 3.97 (s, 2H, N8-CH_2_), 3.88 (s, 2H, 2 × H-9), 3.53 (s, 3H, N1-CH_3_), 3.11 (t, ^3^*J* = 5.35 Hz, 2H, 2 × H-7), 1.21 (t, ^3^*J* = 7.25 Hz, 3H, N3-CH_2_-*CH*_3_). ESI-MS: positive mode 426.3 [M+H]^+^. HPLC: 98.4% (A) and 98.4% (B).

#### 8-(2-chloro-5-(trifluoromethyl)benzyl)-3-ethyl-1-methyl-6,7,8,9-tetrahydropyrazino[2,1-*f*]purine-2,4(1*H*,3*H*)-dione (14m)

General Procedure A. Yield: 64%; mp: 172°C; ^1^H-NMR (CDCl_3_) δ 7.74 (s, 1H, H-6, phenyl), 7.50–7.49 (m, 2H, H-3 and H-4, phenyl), 4.35 (t, ^3^*J* = 5.35 Hz, 2H, 2 × H-6), 4.05 (q, ^3^*J* = 6.90 Hz, 2H, N3-CH_2_), 3.89 (s, 2H, N8-CH_2_), 3.82 (s, 2H, 2 × H-9), 3.53 (s, 3H, N1-CH_3_), 3.02 (t, ^3^*J* = 5.35 Hz, 2H, 2 × H-7), 1.22 (t, ^3^*J* = 7.25 Hz, 3H, N3-CH_2_-*CH*_3_). ESI-MS: positive mode 442.3 [M+H]^+^. HPLC: 99.6% (A) and 98.9% (B).

#### 8-(3-chloro-5-fluorobenzyl)-3-ethyl-1-methyl-6,7,8,9-tetrahydropyrazino[2,1-*f*]purine-2,4(1*H*,3*H*)-dione (14n)

General Procedure A. Yield: 81%; mp: 171°C; ^1^H-NMR (CDCl_3_) δ 7.15 (s, 1H, H-2, phenyl), 7.02 (d, ^3^*J*_H, F_ = 8.20 Hz, 1H, H-4, phenyl), 7.00 (d, ^3^*J*_H, F_ = 9.45 Hz, 1H, H-6, phenyl), 4.37 (t, ^3^*J* = 5.35 Hz, 2H, 2 × H-6), 4.05 (q, ^3^*J* = 6.90 Hz, 2H, N3-CH_2_), 3.76 (s, 2H, N8-CH_2_), 3.72 (s, 2H, 2 × H-9), 3.52 (s, 3H, N1-CH_3_), 2.98 (t, ^3^*J* = 5.35 Hz, 2H, 2 × H-7), 1.22 (t, ^3^*J* = 7.25 Hz, 3H, N3-CH_2_-*CH*_3_). ESI-MS: positive mode 392.2 [M+H]^+^. HPLC: 99.7% (A) and 99.1% (B).

#### 8-(5-bromo-2-fluorobenzyl)-3-ethyl-1-methyl-6,7,8,9-tetrahydropyrazino[2,1-*f*]purine-2,4(1*H*,3*H*)-dione (14o)

General Procedure A. Yield: 71%; mp: 208°C; ^1^H-NMR (CDCl_3_) δ 7.52–7.50 (m, 1H, H-4, phenyl), 7.39–7.35 (m, 1H, H-6, phenyl), 6.96–6.92 (m, 1H, H-3, phenyl), 4.35 (t, ^3^*J* = 5.35 Hz, 2H, 2 × H-6), 4.05 (q, ^3^*J* = 6.90 Hz, 2H, N3-CH_2_), 3.76 (s, 4H, N8-CH_2_ and 2 × H-9), 3.52 (s, 3H, N1-CH_3_), 2.98 (t, ^3^*J* = 5.35 Hz, 2H, 2 × H-7), 1.22 (t, ^3^*J* = 7.25 Hz, 3H, N3-CH_2_-*CH*_3_). ESI-MS: positive mode 436.0 and 438.0 [M+H]^+^. HPLC: 96.4% (A) and 98.5% (B).

#### 3-ethyl-8-(3-fluoro-5-(trifluoromethyl)benzyl)-1-methyl-6,7,8,9-tetrahydropyrazino[2,1-*f*]purine-2,4(1*H*,3*H*)-dione (14p)

General Procedure A. Yield: 66%; mp: 166°C; ^1^H-NMR (CDCl_3_) δ 7.41 (br s, 1H, H-6, phenyl), 7.29–7.25 (m, 2H, H-2 and H-4, phenyl), 4.37 (t, ^3^*J* = 5.05 Hz, 2H, 2 × H-6), 4.05 (q, ^3^*J* = 6.90 Hz, 2H, N3-CH_2_), 3.78 (s, 2H, N8-CH_2_), 3.74 (s, 2H, 2 × H-9), 3.51 (s, 3H, N1-CH_3_), 2.97 (t, ^3^*J* = 5.35 Hz, 2H, 2 × H-7), 1.22 (t, ^3^*J* = 7.25 Hz, 3H, N3-CH_2_-*CH*_3_). ESI-MS: positive mode 426.3 [M+H]^+^. HPLC: 98.4% (A) and 99.6% (B).

#### 8-(3,5-bis(trifluoromethyl)benzyl)-3-ethyl-1-methyl-6,7,8,9-tetrahydropyrazino[2,1-*f*]purine-2,4(1*H*,3*H*)-dione (14q)

General Procedure A. Yield: 51%; mp: 236°C; ^1^H-NMR (CDCl_3_) δ 7.81 (s, 2H, H-2 and H-6, phenyl), 7.80 (s, 1H, H-4, phenyl), 4.37 (t, ^3^*J* = 5.05 Hz, 2H, 2 × H-6), 4.05 (q, ^3^*J* = 6.90 Hz, 2H, N3-CH_2_), 3.85 (s, 2H, N8-CH_2_), 3.76 (s, 2H, 2 × H-9), 3.53 (s, 3H, N1-CH_3_), 2.99 (t, ^3^*J* = 5.35 Hz, 2H, 2 × H-7), 1.22 (t, ^3^*J* = 7.25 Hz, 3H, N3-CH_2_-*CH*_3_). ESI-MS: positive mode 476.1 [M+H]^+^. HPLC: 99.9% (A) and 99.1% (B).

#### 1,3-diethyl-8-(2-fluorobenzyl)-6,7,8,9-tetrahydropyrazino[2,1-*f*]purine-2,4(1*H*,3*H*)-dione (15a)

General procedure A. Yield: 55%; mp: 143°C; ^1^H-NMR (CDCl_3_) δ 7.35 (dd, ^3^*J* = 7.55 Hz, ^3^*J* = 5.95 Hz, ^4^*J* = 1.90 Hz, ^4^*J*_H, F_ = 5.95 Hz, 1H, H-4, phenyl), 7.27 (ddd, ^3^*J* = 5.95 Hz, ^4^*J* = 1.85 Hz, ^3^*J*_H, F_ = 9.45 Hz, 1H, H-3, phenyl), 7.07 (dd, ^3^*J* = 6.25 Hz, ^4^*J* = 1.30 Hz, 1H, H-6, phenyl), 7.02 (pseudo-t, ^3^*J* = 8.55 Hz, ^3^*J* = 5.95 Hz, 2H, H-5, phenyl), 4.33 (t, ^3^*J* = 5.35 Hz, 2H, 2 × H-6), 4.09 (q, ^3^*J* = 7.25 Hz, 2H, N1-CH_2_), 4.04 (q, ^3^*J* = 6.90 Hz, 2H, N3-CH_2_), 3.81 (s, 2H, N8-CH_2_), 3.77 (s, 2H, 2 × H-9), 2.97 (t, ^3^*J* = 5.35 Hz, 2H, 2 × H-7), 1.29 (t, ^3^*J* = 7.25 Hz, 3H, N1-CH_2_-*CH*_3_), 1.21 (t, ^3^*J* = 7.25 Hz, 3H, N3-CH_2_-*CH*_3_). ESI-MS: positive mode 372.2 [M+H]^+^. HPLC: 98.1% (A) and 98.5% (B).

#### 1,3-diethyl-8-(3-fluorobenzyl)-6,7,8,9-tetrahydropyrazino[2,1-*f*]purine-2,4(1*H*,3*H*)-dione (15b)

General procedure A. Yield: 62%; mp: 145°C; ^1^H-NMR (CDCl_3_) δ 7.30 (dd, ^3^*J* = 7.90 Hz, ^4^*J* = 1.90 Hz, 1H, H-5, phenyl), 7.08 (pseudo-t, ^3^*J* = 7.90 Hz, ^3^*J* = 8.20 Hz, 1H, H-4, phenyl), 7.06 (d, ^3^*J* = 8.20 Hz, 1H, H-6, phenyl), 6.98 (m, 1H, ^3^*J* = 7.55 Hz, ^4^*J* = 2.20 Hz, ^3^*J*_H, F_ = 9.80 Hz, H-2, phenyl), 4.33 (t, ^3^*J* = 5.35 Hz, 2H, 2 × H-6), 4.09 (q, ^3^*J* = 7.25 Hz, 2H, N1-CH_2_), 4.04 (q, ^3^*J* = 6.90 Hz, 2H, N3-CH_2_), 3.76 (s, 2H, N8-CH_2_), 3.71 (s, 2H, 2 × H-9), 2.93 (t, ^3^*J* = 5.70 Hz, 2H, 2 × H-7), 1.29 (t, ^3^*J* = 7.25 Hz, 3H, N1-CH_2_-*CH*_3_), 1.21 (t, ^3^*J* = 7.25 Hz, 3H, N3-CH_2_-*CH*_3_). ESI-MS: positive mode 372.1 [M+H]^+^. HPLC: 99.8% (A) and 99.9% (B).

#### 8-(3-chlorobenzyl)-1,3-diethyl-6,7,8,9-tetrahydropyrazino[2,1-*f*]purine-2,4(1*H*,3*H*)-dione (15c)

General procedure A. Yield: 68%; mp: 153°C; ^1^H-NMR (CDCl_3_) δ 7.35–7.34 (m, 1H, H-2, phenyl), 7.27–7.26 (m, 2H, H-5 and H-6, phenyl), 7.22–7.20 (m, 1H, H-4, phenyl), 4.35 (t, ^3^*J* = 5.00 Hz, 2H, 2 × H-6), 4.09 (q, ^3^*J* = 7.25 Hz, 2H, N1-CH_2_), 4.04 (q, ^3^*J* = 6.90 Hz, 2H, N3-CH_2_), 3.83 (br s, 4H, N-8-CH_2_, 2 × H-9), 3.38 (t, ^3^*J* = 5.35 Hz, 2H, 2 × H-7), 1.29 (t, ^3^*J* = 7.25 Hz, 3H, N1-CH_2_-*CH*_3_), 1.21 (t, ^3^*J* = 7.25 Hz, 3H, N3-CH_2_-*CH*_3_). ESI-MS: positive mode 388.3 [M+H]^+^. HPLC: 99.7% (A) and 99.0% (B).

#### 8-(3-bromobenzyl)-1,3-diethyl-6,7,8,9-tetrahydropyrazino[2,1-*f*]purine-2,4(1*H*,3*H*)-dione (15d)

General procedure A. Yield: 81%; mp: 135°C; ^1^H-NMR (CDCl_3_) δ 7.50 (s, 1H, H-2, phenyl), 7.51 (dd, ^3^*J* = 8.20 Hz, ^4^*J* = 1.20 Hz, 1H, H-4, phenyl), 7.42 (d, ^3^*J* = 6.90 Hz, 1H, H-6, phenyl), 7.24 (pseudo-t, ^3^*J* = 7.90 Hz, ^3^*J* = 7.55 Hz, 1H, H-5, phenyl), 7.19 (dd, ^3^*J* = 7.55 Hz, ^3^*J* = 7.55 Hz, 1H, H-5, phenyl), 4.55 (t, ^3^*J* = 5.35 Hz, 2H, 2 × H-6), 4.09 (q, ^3^*J* = 7.25 Hz, 2H, N1-CH_2_), 4.04 (q, ^3^*J* = 6.90 Hz, 2H, N3-CH_2_), 3.72 (s, 2H, N8-CH_2_), 3.69 (s, 2H, 2 × H-9), 2.94 (t, ^3^*J* = 5.05 Hz, 2H, 2 × H-7), 1.29 (t, ^3^*J* = 7.25 Hz, 3H, N1-CH_2_-*CH*_3_), 1.22 (t, ^3^*J* = 7.25 Hz, 3H, N3-CH_2_-*CH*_3_). ESI-MS: positive mode 432.0 and 434.0 [M+H]^+^. HPLC: 99.7% (A) and 99.9% (B).

#### 8-(4-bromobenzyl)-1,3-diethyl-6,7,8,9-tetrahydropyrazino[2,1-*f*]purine-2,4(1*H*,3*H*)-dione (15e)

General procedure A. Yield: 48%; mp: 140°C; ^1^H-NMR (CDCl_3_) δ 7.45 (d, ^3^*J* = 8.20 Hz, 2H, H-3 and H-5, phenyl), 7.21 (d, ^3^*J* = 8.20 Hz, 2H, H-2 and H-6, phenyl), 4.32 (t, ^3^*J* = 5.35 Hz, 2H, 2 × H-6), 4.09 (q, ^3^*J* = 7.25 Hz, 2H, N1-CH_2_), 4.04 (q, ^3^*J* = 6.90 Hz, 2H, N3-CH_2_), 3.72 (s, 2H, N8-CH_2_), 3.67 (s, 2H, 2 × H-9), 2.92 (t, ^3^*J* = 5.35 Hz, 2H, 2 × H-7), 1.29 (t, ^3^*J* = 7.25 Hz, 3H, N1-CH_2_-*CH*_3_), 1.21 (t, ^3^*J* = 7.25 Hz, 3H, N3-CH_2_-*CH*_3_). ESI-MS: positive mode 432.0 and 434.0 [M+H]^+^. HPLC: 99.9% (A) and 99.7% (B).

#### 1,3-diethyl-8-(2-(trifluoromethyl)benzyl)-6,7,8,9-tetrahydropyrazino[2,1-*f*]purine-2,4(1*H*,3*H*)-dione (15f)

General procedure A. Yield: 63%; mp: 142°C; ^1^H-NMR (CDCl_3_) δ 7.72 (d, ^3^*J* = 7.80 Hz, 1H, H-6, phenyl), 7.65 (d, ^3^*J* = 7.80 Hz, 1H, H-2, phenyl), 7.73 (dd, ^3^*J* = 7.50 Hz, ^3^*J* = 7.50 Hz, 1H, H-5, phenyl), 7.65 (dd, ^3^*J* = 7.60 Hz, ^3^*J* = 7.70 Hz, 1H, H-4, phenyl), 4.33 (t, ^3^*J* = 5.35 Hz, 2H, 2 × H-6), 4.09 (q, ^3^*J* = 7.25 Hz, 2H, N1-CH_2_), 4.04 (q, ^3^*J* = 7.25 Hz, 2H, N3-CH_2_), 3.90 (s, 2H, N8-CH_2_), 3.78 (s, 2H, 2 × H-9), 2.97 (t, ^3^*J* = 5.85 Hz, 2H, 2 × H-7), 1.29 (t, ^3^*J* = 7.25 Hz, 3H, N1-CH_2_-*CH*_3_), 1.22 (t, ^3^*J* = 7.25 Hz, 3H, N3-CH_2_-*CH*_3_). ESI-MS: positive mode 422.0 [M+H]^+^. HPLC: 99.9% (A) and 99.3% (B).

#### 1,3-diethyl-8-(3-(trifluoromethyl)benzyl)-6,7,8,9-tetrahydropyrazino[2,1-*f*]purine-2,4(1*H*,3*H*)-dione (15g)

General Procedure A. Yield: 60%; mp: 124°C; ^1^H-NMR (CDCl_3_) δ 7.60 (br s, 2H, H-2, phenyl), 7.55–7.52 (m, 2H, H-5 and H-6, phenyl), 7.47–7.44 (m, 1H, H-4, phenyl), 4.35 (t, ^3^*J* = 5.35 Hz, 2H, 2 × H-6), 4.09 (q, ^3^*J* = 7.25 Hz, 2H, N1-CH_2_), 4.04 (q, ^3^*J* = 7.25 Hz, 2H, N3-CH_2_), 3.78 (s, 2H, N8-CH_2_), 3.73 (s, 2H, 2 × H-9), 2.95 (t, ^3^*J* = 5.35 Hz, 2H, 2 × H-7), 1.30 (t, ^3^*J* = 7.25 Hz, 3H, N1-CH_2_-*CH*_3_), 1.21 (3H, t, ^3^*J* = 7.25 Hz, N3-CH_2_-*CH*_3_). ESI-MS: positive mode 422.0 [M+H]^+^. HPLC: 99.8% (A) and 99.4% (B).

#### 1,3-diethyl-8-(2-fluoro-3-(trifluoromethyl)benzyl)-6,7,8,9-tetrahydropyrazino[2,1-*f*]purine-2,4(1*H*,3*H*)-dione (15h)

General Procedure A. Yield: 35%; mp: 162°C; ^1^H-NMR (CDCl_3_) δ 7.63–7.60 (m, 1H, H-4, phenyl), 7.58–7.55 (m, 1H, H-5, phenyl), 7.26–7.23 (m, 1H, H-6, phenyl), 4.45 (t, ^3^*J* = 5.05 Hz, 2H, 2 × H-6), 4.09 (q, ^3^*J* = 7.25 Hz, 2H, N1-CH_2_), 4.04 (q, ^3^*J* = 6.90 Hz, 2H, N3-CH_2_), 3.97 (s, 2H, N8-CH_2_), 3.88 (s, 2H, 2 × H-9), 3.11 (t, ^3^*J* = 5.35 Hz, 2H, 2 × H-7), 1.30 (t, ^3^*J* = 7.25 Hz, 3H, N1-CH_2_-*CH*_3_), 1.22 (t, ^3^*J* = 7.25 Hz, 3H, N3-CH_2_-*CH*_3_). ESI-MS: positive mode 440.4 [M+H]^+^. HPLC: 99.5% (A) and 99.9% (B).

#### 1,3-diethyl-8-(4-fluoro-3-(trifluoromethyl)benzyl)-6,7,8,9-tetrahydropyrazino[2,1-*f*]purine-2,4(1*H*,3*H*)-dione (15i)

General Procedure A. Yield: 66%; mp: 171°C; ^1^H-NMR (CDCl_3_) δ 7.60–7.58 (m, 1H, H-6, phenyl), 7.54–7.51 (m, 1H, H-2, phenyl), 7.19–7.16 (m, 1H, H-5, phenyl), 4.34 (t, ^3^*J* = 5.05 Hz, 2H, 2 × H-6), 4.10 (q, ^3^*J* = 7.25 Hz, 2H, N1-CH_2_), 4.04 (q, ^3^*J* = 6.90 Hz, 2H, N3-CH_2_), 3.74 (s, 2H, N8-CH_2_), 3.72 (s, 2H, 2 × H-9), 2.95 (t, ^3^*J* = 5.35 Hz, 2H, 2 × H-7), 1.29 (t, ^3^*J* = 7.25 Hz, 3H, N1-CH_2_-*CH*_3_), 1.22 (t, ^3^*J* = 7.25 Hz, 3H, N3-CH_2_-*CH*_3_). ESI-MS: positive mode 440.3 [M+H]^+^. HPLC: 99.5% (A) and 99.0% (B).

#### 1,3-diethyl-8-(2-fluoro-5-(trifluoromethyl)benzyl)-6,7,8,9-tetrahydropyrazino[2,1-*f*]purine-2,4(1*H*,3*H*)-dione (15j)

General Procedure A. Yield: 73%; mp: 154°C; ^1^H-NMR (CDCl_3_) δ 7.70–7.69 (m, 1H, H-6, phenyl), 7.58–7.55 (m, 1H, H-4, phenyl), 7.20–7.16 (m, 1H, H-3, phenyl), 4.36 (t, ^3^*J* = 5.35 Hz, 2H, 2 × H-6), 4.10 (q, ^3^*J* = 7.25 Hz, 2H, N1-CH_2_), 4.04 (q, ^3^*J* = 6.90 Hz, 2H, N3-CH_2_), 3.87 (s, 2H, N8-CH_2_), 3.81 (s, 2H, 2 × H-9), 3.52 (s, 3H, N1-CH_3_), 3.37 (s, 3H, N3-CH_3_), 3.02 (t, ^3^*J* = 5.35 Hz, 2H, 2 × H-7), 1.29 (t, ^3^*J* = 7.25 Hz, 3H, N1-CH_2_-*CH*_3_), 1.22 (t, ^3^*J* = 7.25 Hz, 3H, N3-CH_2_-*CH*_3_). ESI-MS: positive mode 440.3 [M+H]^+^. HPLC: 99.9% (A) and 99.9% (B).

#### 8-(3-bromo-4-fluorobenzyl)-1,3-diethyl-6,7,8,9-tetrahydropyrazino[2,1-*f*]purine-2,4(1*H*,3*H*)-dione (15k)

General Procedure A. Yield: 44%; mp: 156°C; ^1^H-NMR (CDCl_3_) δ 7.56–7.54 (m, 1H, H-2, phenyl), 7.26–7.23 (m, 1H, H-6, phenyl), 7.10–7.06 (m, 1H, H-5, phenyl), 4.35 (t, ^3^*J* = 5.35 Hz, 2H, 2 × H-6), 4.09 (q, ^3^*J* = 7.25 Hz, 2H, N1-CH_2_), 4.04 (q, ^3^*J* = 6.90 Hz, 2H, N3-CH_2_), 3.75 (s, 2H, N8-CH_2_), 3.72 (s, 2H, 2 × H-9), 3.37 (s, 3H, N3-CH_3_), 2.98 (t, ^3^*J* = 5.35 Hz, 2H, 2 × H-7), 1.29 (t, ^3^*J* = 7.25 Hz, 3H, N1-CH_2_-*CH*_3_), 1.21 (t, ^3^*J* = 7.25 Hz, 3H, N3-CH_2_-*CH*_3_). ESI-MS: positive mode 450.3 and 452.3 [M+H]^+^. HPLC: 99.5% (A) and 99.5% (B).

#### 8-(5-bromo-2-fluorobenzyl)-1,3-diethyl-6,7,8,9-tetrahydropyrazino[2,1-*f*]purine-2,4(1*H*,3*H*)-dione (15l)

General Procedure A. Yield: 70%; mp: 190°C; ^1^H-NMR (CDCl_3_) δ 7.53–7.51 (m, 1H, H-4, phenyl), 7.40–7.37 (m, 1H, H-6, phenyl), 6.97–6.93 (m, 1H, H-3, phenyl), 4.35 (t, ^3^*J* = 5.35 Hz, 2H, 2 × H-6), 4.10 (q, ^3^*J* = 7.25 Hz, 2H, N1-CH_2_), 4.04 (q, ^3^*J* = 6.90 Hz, 2H, N3-CH_2_), 3.77 (s, 4H, 2 × H-9 and N8-CH_2_), 2.98 (t, ^3^*J* = 5.35 Hz, 2H, 2 × H-7), 1.29 (t, ^3^*J* = 7.25 Hz, 3H, N1-CH_2_-*CH*_3_), 1.21 (t, ^3^*J* = 7.25 Hz, 3H, N3-CH_2_-*CH*_3_). ESI-MS: positive mode 436.0 and 438.0 [M+H]^+^. HPLC: 99.5% (A) and 99.4% (B).

#### 8-(3,5-dichlorobenzyl)-1,3-diethyl-6,7,8,9-tetrahydropyrazino[2,1-*f*]purine-2,4(1*H*,3*H*)-dione (15m)

General Procedure A. Yield: 70%; mp: 182°C; ^1^H-NMR (CDCl_3_) δ 7.28 (d, ^4^*J* = 1.90 Hz, 1H, H-4, phenyl), 7.24 (d, ^4^*J* = 1.90 Hz, 2H, H-2 and H-6, phenyl), 4.36 (t, ^3^*J* = 5.35 Hz, 2H, 2 × H-6), 4.09 (q, ^3^*J* = 7.25 Hz, 2H, N1-CH_2_), 4.04 (q, ^3^*J* = 6.90 Hz, 2H, N3-CH_2_), 3.73 (s, 2H, N8-CH_2_), 3.68 (s, 2H, 2 × H-9), 2.95 (t, ^3^*J* = 5.35 Hz, 2H, 2 × H-7), 1.30 (t, ^3^*J* = 7.25 Hz, 3H, N1-CH_2_-*CH*_3_), 1.22 (t, ^3^*J* = 7.25 Hz, 3H, N3-CH_2_-*CH*_3_). ESI-MS: positive mode 423.3 [M+H]^+^. HPLC: 98.0% (A) and 99.6% (B).

#### 8-(2-chloro-5-(trifluoromethyl)benzyl)-1,3-diethyl-6,7,8,9-tetrahydropyrazino[2,1-*f*]purine-2,4(1*H*,3*H*)-dione (15n)

General Procedure A. Yield: 60%; mp: 152°C; ^1^H-NMR (CDCl_3_) δ 7.74 (s, 1H, H-6, phenyl), 7.50–7.49 (m, 2H, H-3 and H-4, phenyl), 4.38 (t, ^3^*J* = 5.35 Hz, 2H, 2 × H-6), 4.10 (q, ^3^*J* = 7.25 Hz, 2H, N1-CH_2_), 4.05 (q, ^3^*J* = 6.90 Hz, 2H, N3-CH_2_), 3.89 (s, 2H, N8-CH_2_), 3.83 (s, 2H, 2 × H-9), 3.01 (t, ^3^*J* = 5.35 Hz, 2H, 2 × H-7), 1.30 (t, ^3^*J* = 7.25 Hz, 3H, N1-CH_2_-*CH*_3_), 1.22 (t, ^3^*J* = 7.25 Hz, 3H, N3-CH_2_-*CH*_3_). ESI-MS: positive mode 442.3 [M+H]^+^. HPLC: 99.5% (A) and 99.4% (B).

#### 8-(4-chloro-2-(trifluoromethyl)benzyl)-1,3-diethyl-6,7,8,9-tetrahydropyrazino[2,1-*f*]purine-2,4(1*H*,3*H*)-dione (15o)

General Procedure A. Yield: 27%; mp: 188°C; ^1^H-NMR (CDCl_3_) δ 7.69 (d, ^3^*J* = 8.55 Hz, 1H, H-6, phenyl), 7.64 (dd, ^3^*J* = 8.55 Hz, ^4^*J* = 1.90 Hz, 1H, H-5, phenyl), 7.49 (dd, ^3^*J* = 8.55 Hz, ^4^*J* = 2.20 Hz, 1H, H-3, phenyl), 4.34 (t, ^3^*J* = 5.35 Hz, 2H, 2 × H-6), 4.10 (q, ^3^*J* = 7.25 Hz, 2H, N1-CH_2_), 4.04 (q, ^3^*J* = 6.90 Hz, 2H, N3-CH_2_), 3.86 (s, 2H, N8-CH_2_), 3.77 (s, 2H, 2 × H-9), 2.95 (t, ^3^*J* = 5.35 Hz, 2H, 2 × H-7), 1.30 (t, ^3^*J* = 7.25 Hz, 3H, N1-CH_2_-*CH*_3_), 1.22 (t, ^3^*J* = 7.25 Hz, 3H, N3-CH_2_-*CH*_3_). ESI-MS: positive mode 428.0 [M+H]^+^. HPLC: 98.9% (A) and 99.4% (B).

#### 8-(4-chloro-3-(trifluoromethyl)benzyl)-1,3-diethyl-6,7,8,9-tetrahydropyrazino[2,1-*f*]purine-2,4(1*H*,3*H*)-dione (15p)

General Procedure A. Yield: 38%; mp: 165°C; ^1^H-NMR (CDCl_3_) δ 7.67 (s, 1H, H-2, phenyl), 7.48 (d, ^3^*J* = 8.20 Hz, 1H, H-6, phenyl), 7.45 (d, ^3^*J* = 8.20 Hz, 1H, H-5, phenyl), 4.35 (t, ^3^*J* = 5.05 Hz, 2H, 2 × H-6), 4.10 (q, ^3^*J* = 7.25 Hz, 2H, N1-CH_2_), 4.04 (q, ^3^*J* = 6.90 Hz, 2H, N3-CH_2_), 3.76 (s, 2H, N8-CH_2_), 3.73 (s, 2H, 2 × H-9), 3.51 (s, 3H, N1-CH_3_), 3.37 (s, 3H, N3-CH_3_), 2.96 (t, ^3^*J* = 5.35 Hz, 2H, 2 × H-7), 1.29 (t, ^3^*J* = 7.25 Hz, 3H, N1-CH_2_-*CH*_3_), 1.21 (t, ^3^*J* = 7.25 Hz, 3H, N3-CH_2_-*CH*_3_). ESI-MS: positive mode 456.3 [M+H]^+^. HPLC: 99.7% (A) and 99.7% (B).

#### 8-(3,5-bis(trifluoromethyl)benzyl)-1,3-diethyl-6,7,8,9-tetrahydropyrazino[2,1-*f*]purine-2,4(1*H*,3*H*)-dione (15q)

General Procedure A. Yield: 51%; mp: 141°C; ^1^H-NMR (CDCl_3_) δ 7.81 (br s, 3H, H-2, H-4 and H-6, phenyl), 4.37 (t, ^3^*J* = 5.05 Hz, 2H, 2 × H-6), 4.09 (q, ^3^*J* = 7.25 Hz, 2H, N1-CH_2_), 4.04 (q, ^3^*J* = 6.90 Hz, 2H, N3-CH_2_), 3.85 (s, 2H, N8-CH_2_), 3.76 (s, 2H, 2 × H-9), 2.99 (t, ^3^*J* = 5.35 Hz, 2H, 2 × H-7), 1.29 (t, ^3^*J* = 7.25 Hz, 3H, N1-CH_2_-*CH*_3_), 1.21 (t, ^3^*J* = 7.25 Hz, 3H, N3-CH_2_-*CH*_3_). ESI-MS: positive mode 490.4 [M+H]^+^. HPLC: 99.7% (A) and 99.7% (B).

#### 1,3-diethyl-8-(3,4,5-trifluorobenzyl)-6,7,8,9-tetrahydropyrazino[2,1-*f*]purine-2,4(1*H*,3*H*)-dione (15r)

General Procedure A. Yield: 43%; mp: 166°C; ^1^H-NMR (CDCl_3_) δ 7.00–6.97 (m, 2H, H-2 and H-6, phenyl), 4.33 (t, ^3^*J* = 5.35 Hz, 2H, 2 × H-6), 4.10 (q, ^3^*J* = 7.25 Hz, 2H, N1-CH_2_), 4.04 (q, ^3^*J* = 6.90 Hz, 2H, N3-CH_2_), 3.73 (s, 2H, N8-CH_2_), 3.66 (s, 2H, 2 × H-9), 2.96 (t, ^3^*J* = 5.35 Hz, 2H, 2 × H-7), 1.30 (t, ^3^*J* = 7.25 Hz, 3H, N1-CH_2_-*CH*_3_), 1.22 (t, ^3^*J* = 7.25 Hz, 3H, N3-CH_2_-*CH*_3_). ESI-MS: positive mode 408.3 [M+H]^+^. HPLC: 97.8% (A) and 96.5% (B).

#### Preparation of 8-substituted 6,7,8,9-tetrahydropyrazino[2,1-*f*]purine-2,4(1*H*,3*H*)-diones 16–17 via alkylation of the 3-position (general procedure B)

The 3-unsubstituted tetrahydropyrazino[2,1-*f* ]purinediones **11a**, **11b** or **12** (0.25 mmol), potassium *tert*-butoxide (56 mg, 0.5 mmol) and an alkylating agent (1.5 mmol, 6 eq.) were dissolved in 4 mL of dry THF and stirred for 4 h at rt under argon. The progress of the reaction was checked by TLC after 3 h and, if necessary, a further 4 eq. of the alkylating agent was added to drive the reaction to completion. The volatiles were removed by rotary evaporation and the product was purified by silica gel column chromatography using a gradient of CH_2_Cl_2_ to CH_2_Cl_2_/MeOH 40:1 as eluent.

#### 8-(3,4-dichlorobenzyl)-1-ethyl-3-methyl-6,7,8,9-tetrahydropyrazino[2,1-*f*]purine-2,4(1*H*,3*H*)-dione (16a)

General procedure B starting from **11a**. Yield: 31%; mp: 164°C; ^1^H-NMR (CDCl_3_) δ 7.44 (d, ^4^*J* = 1.90 Hz, 1H, H-2, phenyl), 7.39 (d, ^3^*J* = 8.20 Hz, 1H, H-5, phenyl), 7.17 (dd, ^3^*J* = 8.20 Hz and ^4^*J* = 2.40 Hz, 1H, H-6, phenyl), 4.33 (t, ^3^*J* = 5.35 Hz, 2H, 2 × H-6), 4.08 (q, ^3^*J* = 7.25 Hz, 2H, N1-CH_2_), 3.72 (s, 2H, N8-CH_2_), 3.68 (s, 2H, 2 × H-9), 3.36 (s, 3H, N3-CH_3_), 2.93 (t, ^3^*J* = 5.35 Hz, 2H, 2 × H-7), 1.28 (t, ^3^*J* = 7.25 Hz, 3H, N1-CH_2_-*CH*_3_). ESI-MS: positive mode 408.3 [M+H]^+^. HPLC: 99.7% (A) and 99.7% (B).

#### 8-(3,5-dichlorobenzyl)-1-ethyl-3-methyl-6,7,8,9-tetrahydropyrazino[2,1-*f*]purine-2,4(1*H*,3*H*)-dione (16b)

General procedure B starting from **11b**. Yield: 32%; mp: 183°C; ^1^H-NMR (CDCl_3_) δ 7.29 (d, ^4^*J* = 1.90 Hz, 1H, H-4, phenyl), 7.24 (s, 2H, H-2 and H-6, phenyl), 4.35 (t, ^3^*J* = 5.35 Hz, 2H, 2 × H-6), 4.09 (q, ^3^*J* = 7.25 Hz, 2H, N1-CH_2_), 3.73 (s, 2H, N8-CH_2_), 3.68 (s, 2H, 2 × H-9), 3.35 (s, 3H, N3-CH_3_), 2.95 (t, ^3^*J* = 5.35 Hz, 2H, 2 × H-7), 1.29 (t, ^3^*J* = 7.25 Hz, 3H, N1-CH_2_-*CH*_3_). ESI-MS: positive mode 408.3 [M+H]^+^. HPLC: 99.7% (A) and 99.7% (B).

#### 8-(3,4-dichlorobenzyl)-1-cyclopropyl-3-methyl-6,7,8,9-tetrahydropyrazino[2,1-*f*]purine-2,4(1*H*,3*H*)-dione (17)

General procedure B starting from **12**. Yield: 35%; mp: 200°C; ^1^H-NMR (CDCl_3_) δ 7.44 (d, ^4^*J* = 1.85 Hz, 1H, H-2, phenyl), 7.40 (d, ^3^*J* = 8.20 Hz, 1H, H-5, phenyl), 7.16 (dd, ^3^*J* = 8.20 Hz, ^4^*J* = 1.90 Hz, 1H, H-6, phenyl), 4.33 (t, ^3^*J* = 5.35 Hz, 2H, 2 × H-6), 3.74 (s, 2H, N8-CH_2_), 3.68 (s, 2H, 2 × H-9), 3.34 (s, 3H, N3-CH_3_), 2.94–2.92 (m, 3H, 2 × H-7 and H-1, cyclopropyl), 1.19–1.12 (m, 2H, H-2 and H-3, cyclopropyl), 1.00–0.96 (m, 2H, H-2 and H-3, cyclopropyl). ESI-MS: positive mode 420.1 [M+H]^+^. HPLC: 98.2% (A) and 97.5% (B).

#### Preparation of 8-substituted 6,7,8,9-tetrahydropyrazino[2,1-*f*]purine-2,4(1*H*,3*H*)-diones 18–19 via alkylation of the 3-position (general procedure C)

The 3-unsubstituted tetrahydropyrazino[2,1-*f* ]purinediones **13h** or **13i** (0.25 mmol), sodium hydride (60% in mineral oil) and the appropriate alkylating agent were dissolved in dry DMF and stirred for 4 h at rt under argon. The volatiles were removed by rotary evaporation and the product was purified by flash-chromatography (silica gel, CH_2_Cl_2_: CH_2_Cl_2_/MeOH 1:0 to 40:1).

#### 8-(3,4-dichlorobenzyl)-1-methyl-3-propargyl-6,7,8,9-tetrahydropyrazino[2,1-*f*]purine-2,4(1*H*,3*H*)-dione (18)

General procedure C starting from **13h**. Yield: 43%; mp: 159°C; ^1^H-NMR (CDCl_3_) δ 7.44 (d, ^4^*J* = 2.00 Hz, 1H, H-2, phenyl), 7.40 (d, ^3^*J* = 8.20 Hz, 1H, H-5, phenyl), 7.17 (dd, ^3^*J* = 8.20 Hz, ^4^*J* = 2.00 Hz, 1H, H-6, phenyl), 4.34 (t, ^3^*J* = 5.45 Hz, 2H, 2 × H-6), 3.95–3.92 (m, 2H, N3-CH_2_), 3.72 (s, 2H, N8-CH_2_), 3.68 (s, 2H, 2 × H-9), 3.51 (s, 3H, N1-CH_3_), 2.95–2.92 (m, 2H, 2 × H-7), 1.69–1.62 (m, 3H, N3-CH_2_-*CH*_2_), 0.93 (t, ^3^*J* = 7.45 Hz, 3H, N3-CH_2_-CH_2_-*CH*_3_). ESI-MS: positive mode 422.2 [M+H]^+^. HPLC: 96.9% (C).

#### 8-(3,4-dichlorobenzyl)-1-methyl-3-propargyl-6,7,8,9-tetrahydropyrazino[2,1-*f*]purine-2,4(1*H*,3*H*)-dione (19a)

General procedure C starting from **13h**. Yield: 58%; mp: 228°C; ^1^H-NMR (CDCl_3_) δ 7.44 (d, ^4^*J* = 2.00 Hz, 1H, H-2, phenyl), 7.41 (d, ^3^*J* = 8.20 Hz, 1H, H-5, phenyl), 7.17 (dd, ^3^*J* = 8.20 Hz, ^4^*J* = 2.00 Hz, 1H, H-6, phenyl), 4.76 (d, ^4^*J* = 2.45 Hz, 2H, N3-CH_2_), 4.35 (t, ^3^*J* = 5.45 Hz, 2H, 2 × H-6), 3.73 (s, 2H, N8-CH_2_), 3.68 (s, 2H, 2 × H-9), 3.53 (s, 3H, N1-CH_3_), 2.95–2.93 (m, 2H, 2 × H-7), 2.15 (t, ^4^*J* = 2.45 Hz, 1H, N3-CH_2_-*CH*). ESI-MS: positive mode 418.2 [M+H]^+^. HPLC: 98.6% (C).

#### 8-(2-chloro-5-(trifluoromethyl)benzyl)-1-methyl-3-propargyl-6,7,8,9-tetrahydropyrazino[2,1-*f*]purine-2,4(1*H*,3*H*)-dione (19b)

General procedure C starting from **13i**. Yield: 28%; mp: 211°C; ^1^H-NMR (CDCl_3_) δ 7.73 (s, 1H, H-6, phenyl), 7.50 (2 br s, 2H, H-3 and H-4, phenyl), 4.76 (t, ^3^*J* = 5.35 Hz, 2H, 2 × H-6), 4.38 (s, 2H, N3-CH_2_), 3.89 (s, 2H, N8-CH_2_), 3.83 (s, 2H, 2 × H-9), 3.54 (s, 3H, N1-CH_3_), 3.02 (t, ^3^*J* = 5.35 Hz, 2H, 2 × H-7), 2.15 (s, 1H, N3-CH_2_-*CH*). ESI-MS: positive mode 442.3 [M+H]^+^. HPLC: 98.6% (A) and 98.7% (B).

#### Synthesis of 8-substituted 1-ethyl-3-propargyl-8-6,7,8,9-tetrahydropyrazino[2,1-*f*]purine-2,4(1*H*,3*H*)-diones (20) (general procedure D)

7-(2-Bromoethyl)-8-*N-*boc-aminomethyl-3-methyl-1-propargylxanthine (**28**) (150 mg, 0.33 mmol) was stirred in a solution of 4N-HCl in dry dioxane (4 mL) for 30 min at rt. The deprotected xanthine **29** precipitated upon addition of diethylether (30 mL). It was filtered off, washed with diethylether (3 × 10 mL) and used directly in the next step. The xanthine **29** was dissolved in a mixture of 1,2-dimethoxyethane (10 mL) and DIPEA (0.5 mL) and stirred for 6 h at rt. Then, the appropriate halide (0.5 mmol) was added and the solution was stirred overnight at rt. The volatiles were removed in vacuo and the final product **20** was purified by column chromatography.

#### 1-ethyl-8-(2-fluorophenyl)-3-propargyl-6,7,8,9-tetrahydropyrazino[2,1-*f*]purine-2,4(1*H*,3*H*)-dione (20a)

General procedure D. Yield: 28%; mp: 151°C; ^1^H-NMR (CDCl_3_) δ 7.03–7.00 (m, 2H, H-3 and H-4, phenyl), 6.95–6.92 (m, 2H, H-5 and H-6, phenyl), 4.77 (d, ^4^*J* = 2.20 Hz, 2H, N3-CH_2_), 4.50 (s, 2H, 2 × H-9), 4.45 (t, ^3^*J* = 5.35 Hz, 2H, 2 × H-6), 4.16 (q, ^3^*J* = 7.25 Hz, 2H, N1-CH_2_), 3.71 (t, ^3^*J* = 5.35 Hz, 2H, 2 × H-7), 2.15 (t, ^4^*J* = 2.50 Hz, 1H, N3-CH_2_-*CH*), 1.35 (t, ^3^*J* = 7.25 Hz, 3H, N1-CH_2_-*CH*_3_). ESI-MS: positive mode 368.0 [M+H]^+^. HPLC: 95.2% (A) and 95.5% (B).

#### 1-ethyl-8-(4-fluorophenyl)-3-propargyl-6,7,8,9-tetrahydropyrazino[2,1-*f*]purine-2,4(1*H*,3*H*)-dione (20b)

General procedure D. Yield: 31%; mp: 157°C; ^1^H-NMR (CDCl_3_) δ 6.83–6.70 (m, 4H, ^3^*J* = 8.20 Hz, and ^4^*J* = 2.20 Hz, H-2, H-3, H-5 and H-6, phenyl), 4.77 (d, ^4^*J* = 2.20 Hz, 2H, N3-CH_2_), 4.44 (s, 2H, 2 × H-9), 4.18 (q, ^3^*J* = 7.25 Hz, 2H, N1-CH_2_), 3.81 (t, ^3^*J* = 5.35 Hz, 2H, 2 × H-6), 3.62 (t, ^3^*J* = 5.35 Hz, 2H, 2 × H-7), 2.15 (t, ^4^*J* = 2.50 Hz, 1H, N3-CH_2_-*CH*), 1.35 (t, ^3^*J* = 7.25 Hz, 3H, N1-CH_2_-*CH*_3_). ESI-MS: positive mode 368.0 [M+H]^+^. HPLC: 94.1% (A) and 94.3% (B).

#### 1-ethyl-8-(3-methoxyphenyl)-3-propargyl-6,7,8,9-tetrahydropyrazino[2,1-*f*]purine-2,4(1*H*,3*H*)-dione (20c)

General procedure D. Yield: 23%; mp: 169°C; ^1^H-NMR (CDCl_3_) δ 7.26 (pseudo-t, ^3^*J* = 8.20 Hz, 1H, H-5, phenyl), 6.57 (dd, ^3^*J* = 8.15 Hz, and ^4^*J* = 2.55 Hz, 1H, H-6, phenyl), 6.51 (dd, ^3^*J* = 7.90 Hz, and ^4^*J* = 1.85 Hz, 1H, H-4, phenyl), 6.50 (s, 1H, H-2, phenyl), 4.77 (d, ^4^*J* = 2.20 Hz, 2H, N3-CH_2_), 4.50 (s, 2H, 2 × H-9), 4.45 (t, ^3^*J* = 5.35 Hz, 2H, 2 × H-6), 4.16 (q, ^3^*J* = 7.25 Hz, 2H, N1-CH_2_), 3.79 (s, 3H, OCH_3_), 3.71 (t, ^3^*J* = 5.35 Hz, 2H, 2 × H-7), 2.15 (t, ^4^*J* = 2.50 Hz, 1H, N3-CH_2_-*CH*), 1.35 (t, ^3^*J* = 7.25 Hz, 3H, N1-CH_2_-*CH*_3_). ESI-MS: positive mode 380.4 [M+H]^+^. HPLC: 96.3% (A) and 95.1% (B).

#### 8-(3,4-dimethoxyphenyl)-1-ethyl-3-propargyl-6,7,8,9-tetrahydropyrazino[2,1-*f*]purine-2,4(1*H*,3*H*)-dione (20d)

General procedure D. Yield: 28%; mp: 125°C; ^1^H-NMR (CDCl_3_) δ 6.81 (d, ^3^*J* = 8.80 Hz, 1H, H-6, phenyl), 6.59 (d, ^4^*J* = 2.55 Hz, 1H, H-2, phenyl), 6.48 (dd, ^3^*J* = 8.50 Hz, ^4^*J* = 2.80 Hz, 1H, H-6, phenyl), 4.77 (d, ^4^*J* = 2.20 Hz, 2H, N3-CH_2_), 4.45 (t, ^3^*J* = 5.35 Hz, 2H, 2 × H-6), 4.41 (s, 2H, 2 × H-9), 4.16 (q, ^3^*J* = 7.25 Hz, 2H, N1-CH_2_), 3.87 (s, 3H, OCH_3_), 3.84 (s, 3H, OCH_3_), 3.60 (t, ^3^*J* = 5.35 Hz, 2H, 2 × H-7), 2.15 (t, ^4^*J* = 2.50 Hz, 1H, N3-CH_2_-*CH*), 1.35 (t, ^3^*J* = 7.25 Hz, 3H, N1-CH_2_-*CH*_3_). ESI-MS: positive mode 410.4 [M+H]^+^. HPLC: 97.0% (A) and 95.7% (B).

#### 8-benzyl-1-ethyl-3-propargyl-6,7,8,9-tetrahydropyrazino[2,1-*f*]purine-2,4(1*H*,3*H*)-dione (20e)

General procedure D. Yield: 19%; mp: 148°C; ^1^H-NMR (CDCl_3_) δ 7.64–7.62 (m, 2H, H-3 and H-5, phenyl), 7.43–7.39 (m, 3H, H-2, H-4 and H-6, phenyl), 4.86 (s, 2H, N8-CH_2_), 4.64 (d, ^4^*J* = 2.20 Hz, 2H, N3-CH_2_), 4.58 (s, 2H, 2 × H-9), 4.45 (t, ^3^*J* = 5.35 Hz, 2H, 2 × H-6), 3.98 (q, ^3^*J* = 7.25 Hz, 2H, N1-CH_2_), 3.83 (t, ^3^*J* = 5.35 Hz, 2H, 2 × H-7), 2.16 (t, ^4^*J* = 2.50 Hz, 1H, N3-CH_2_-*CH*), 1.21 (t, ^3^*J* = 7.25 Hz, 3H, N1-CH_2_-*CH*_3_). ESI-MS: positive mode: 364.1 [M+H]^+^. HPLC: 97.0% (A) and 96.7% (B).

#### 1-ethyl-8-(2-methoxybenzyl)-3-propargyl-6,7,8,9-tetrahydropyrazino[2,1-*f*]purine-2,4(1*H*,3*H*)-dione (20f)

General procedure D. Yield: 26%; mp: 180°C; ^1^H-NMR (CDCl_3_) δ 7.31–7.29 (m, 1H, H-4, phenyl), 7.28–7.25 (m, 1H, H-6, phenyl), 6.95–6.91 (m, 1H, H-5, phenyl), 6.88 (d, ^3^*J* = 8.20 Hz, 1H, H-3, phenyl), 4.75 (d, ^4^*J* = 2.50 Hz, 2H, N3-CH_2_), 4.33 (t, ^3^*J* = 5.35 Hz, 2H, 2 × H-6), 4.12 (q, ^3^*J* = 7.25 Hz, 2H, N1-CH_2_), 3.82 (s, 3H, OCH_3_), 3.78 (s, 4H, 2 × H-9 and N8-CH_2_), 2.97 (t, ^3^*J* = 5.35 Hz, 2H, 2 × H-7), 2.13 (t, ^4^*J* = 2.50 Hz, 1H, N3-CH_2_-*CH*), 1.30 (t, ^3^*J* = 7.25 Hz, 3H, N1-CH_2_-*CH*_3_). ESI-MS: positive mode 394.3 [M+H]^+^. HPLC: 97.3% (A) and 98.1% (B).

#### 1-ethyl-8-(3-methoxybenzyl)-3-propargyl-6,7,8,9-tetrahydropyrazino[2,1-*f*]purine-2,4(1*H*,3*H*)-dione (20g)

General procedure D. Yield: 19%; mp: 157°C; ^1^H-NMR (CDCl_3_) δ 7.22 (dd, ^3^*J* = 6.95 Hz, ^3^*J* = 7.60 Hz, 1H, H-5, phenyl), 6.90 (s, 1H, H-2, phenyl), 6.88 (d, ^3^*J* = 6.80 Hz, 1H, H-4, phenyl), 6.82 (dd, ^3^*J* = 7.25 Hz, ^4^*J* = 1.55 Hz, 1H, H-6, phenyl), 4.75 (d, ^4^*J* = 2.55 Hz, 2H, N3-CH_2_), 4.33 (t, ^3^*J* = 5.35 Hz, 2H, 2 × H-6), 4.12 (q, ^3^*J* = 7.25 Hz, 2H, N1-CH_2_), 3.79 (s, 3H, OCH_3_), 3.74 (s, 2H, N8-CH_2_), 3.70 (s, 2H, 2 × H-9), 2.93 (t, ^3^*J* = 5.35 Hz, 2H, 2 × H-7), 2.14 (t, ^4^*J* = 2.50 Hz, 1H, N3-CH_2_-*CH*), 1.30 (t, ^3^*J* = 7.25 Hz, 3H, N1-CH_2_-*CH*_3_). ESI-MS: positive mode 394.3 [M+H]^+^. HPLC: 98.1% (A) and 98.8% (B).

#### 1-ethyl-8-phenethyl-3-propargyl-6,7,8,9-tetrahydropyrazino[2,1-*f*]purine-2,4(1h,3h)-dione (20h)

General procedure D. Yield: 20%; mp: 144°C; ^1^H-NMR (CDCl_3_) δ 7.33–7.20 (m, 5H, H-2-H-6, phenyl), 4.74 (d, ^4^*J* = 2.55 Hz, 2H, N3-CH_2_), 4.56 (t, ^3^*J* = 5.35 Hz, 2H, 2 × H-6), 4.26 (s, 2H, 2 × H-9), 4.11 (q, ^3^*J* = 7.25 Hz, 2H, N1-CH_2_), 3.43 (t, ^3^*J* = 5.35 Hz, 2H, 2 × H-7), 3.22 (t, ^3^*J* = 7.55 Hz, 2H, N8-CH_2_), 3.02 (t, ^3^*J* = 7.55 Hz, 2H, N8-CH_2_-*CH*_2_), 2.15 (t, ^4^*J* = 2.50 Hz, 1H, N3-CH_2_-*CH*), 1.35 (t, ^3^*J* = 7.25 Hz, 3H, N1-CH_2_-*CH*_3_). ESI-MS: positive mode: 378.4 [M+H]^+^. HPLC: 98.2% (A) and 98.8% (B).

#### 1-ethyl-8-(2-methoxyphenethyl)-3-propargyl-6,7,8,9-tetrahydropyrazino[2,1-*f*]purine-2,4(1*H*,3*H*)-dione (20i)

General procedure D. Yield: 24%; mp: 167°C; ^1^H-NMR (CDCl_3_) δ 7.21–7.18 (m, 1H, H-6, phenyl), 7.12–7.10 (m, 1H, H-4, phenyl), 6.89–6.82 (m, 2H, H-3 and H-5, phenyl), 4.76 (d, ^4^*J* = 2.20 Hz, 2H, N3-CH_2_), 4.34 (t, ^3^*J* = 5.35 Hz, 2H, 2 × H-6), 4.33 (s, 2H, 2 × H-9), 4.14 (q, ^3^*J* = 7.25 Hz, 2H, N1-CH_2_), 3.85 (s, 3H, OCH_3_), 3.80 (t, ^3^*J* = 5.35 Hz, 2H, 2 × H-7), 3.32 (t, ^3^*J* = 7.55 Hz, 2H, N8-CH_2_), 2.97 (t, ^3^*J* = 7.55 Hz, 2H, N8-CH_2_-*CH*_2_), 2.15 (t, ^4^*J* = 2.50 Hz, 1H, N3-CH_2_-*CH*), 1.33 (t, ^3^*J* = 7.25 Hz, 3H, N1-CH_2_-*CH*_3_). ESI-MS: positive mode 408.3 [M+H]^+^. HPLC: 95.6% (A) and 96.3% (B).

#### 1-ethyl-8-(3-methoxyphenethyl)-3-propargyl-6,7,8,9-tetrahydropyrazino[2,1-*f*]purine-2,4(1*H*,3*H*)-dione (20j)

General procedure D. Yield: 21%; mp: 212°C; ^1^H-NMR (CDCl_3_) δ 7.21–7.18 (m, 1H, H-5, phenyl), 6.80–6.78 (m, H-6, phenyl), 6.76–6.74 (m, 2H, H-2 and H-4, phenyl), 4.75 (d, ^4^*J* = 2.50 Hz, 2H, N3-CH_2_), 4.33 (t, ^3^*J* = 5.35 Hz, 2H, 2 × H-6), 4.13 (q, ^3^*J* = 7.25 Hz, 2H, N1-CH_2_), 3.82 (s, 2H, 2 × H-9), 3.77 (s, 3H, OCH_3_), 2.96 (t, ^3^*J* = 5.35 Hz, 2H, 2 × H-7), 2.83 (br s, 4H, N8-CH_2_-CH_2_), 2.14 (t, ^4^*J* = 2.55 Hz, 1H, N3-CH_2_-*CH*), 1.35 (t, ^3^*J* = 7.25 Hz, 3H, N1-CH_2_-*CH*_3_). ESI-MS: positive mode 408.3 [M+H]^+^. HPLC: 97.0% (A) and 98.5% (B).

#### 1-ethyl-8-(4-methoxyphenethyl)-3-propargyl-6,7,8,9-tetrahydropyrazino[2,1-*f*]purine-2,4(1*H*,3*H*)-dione (20k)

General procedure D. Yield: 19%; mp: 164°C; ^1^H-NMR (CDCl_3_) δ 7.11 (dd, ^3^*J* = 8.50 Hz, ^4^*J* = 2.20 Hz, 2H, H-2 and H-6, phenyl), 6.84 (dd, ^3^*J* = 8.50 Hz, ^4^*J* = 2.20 Hz, 2H, H-3 and H-5, phenyl), 4.75 (d, ^4^*J* = 2.50 Hz, 2H, N3-CH_2_), 4.45 (t, ^3^*J* = 5.35 Hz, 2H, 2 × H-6), 4.22 (s, 2H, 2 × H-9), 4.12 (q, ^3^*J* = 7.25 Hz, 2H, N1-CH_2_), 3.77 (s, 3H, OCH_3_), 3.39 (t, ^3^*J* = 5.35 Hz, 2H, 2 × H-7), 3.16 (t, ^3^*J* = 7.55 Hz, 2H, N8-CH_2_), 2.96 (t, ^3^*J* = 7.55 Hz, 2H, N8-CH_2_-*CH*_2_), 2.15 (t, ^4^*J* = 2.50 Hz, 1H, N3-CH_2_-*CH*), 1.31 (t, ^3^*J* = 7.25 Hz, 3H, N1-CH_2_-*CH*_3_). ESI-MS: positive mode 408.3 [M+H]^+^. HPLC: 95.0% (A) and 94.1% (B).

#### 1-ethyl-8-(2,3-dimethoxyphenethyl)-3-propargyl-6,7,8,9-tetrahydropyrazino[2,1-*f*]purine-2,4(1*H*,3*H*)-dione (20l)

General procedure D. Yield: 25%; mp: 141°C; ^1^H-NMR (CDCl_3_) δ 6.80–6.71 (m, 3H, H-2, H-5 and H-6, phenyl), 4.77 (d, ^4^*J* = 2.50 Hz, 2H, N3-CH_2_), 4.45 (t, ^3^*J* = 5.35 Hz, 2H, 2 × H-6), 4.39 (s, 2H, 2 × H-9), 4.11 (q, ^3^*J* = 7.25 Hz, 2H, N1-CH_2_), 3.84 (s, 3H, OCH_3_), 3.85 (s, 3H, OCH_3_), 3.57 (t, ^3^*J* = 5.35 Hz, 2H, 2 × H-7), 3.32 (t, ^3^*J* = 7.55 Hz, 2H, N8-CH_2_), 3.02 (t, ^3^*J* = 7.55 Hz, 2H, N8-CH_2_-*CH*_2_), 2.16 (t, ^4^*J* = 2.20 Hz, 1H, N3-CH_2_-*CH*), 1.31 (t, ^3^*J* = 7.25 Hz, 3H, N1-CH_2_-*CH*_3_). ESI-MS: positive mode 438.4 [M+H]^+^. HPLC: 97.7% (A) and 97.2% (B).

### Biological evaluation

#### Radioligand binding assays at adenosine receptors

The following highly (>100-fold) selective radioligands were employed: A_1_ARs, [^3^H]2-chloro-N^6^cyclopentyladenosine ([^3^H]CCPA, Klotz et al., 1989, 1 nM, K_D_ human A_1_: 0.61 nM, K_D_: rat A_1_: 0.2 nM); A_2A_ARs, [^3^H]3-(3-hydroxypropyl)-7-methyl-8-(*m*-methoxystyryl)-1-propargylxanthine ([^3^H]MSX-2, Müller et al., 2000, 1 nM, K_D_ human A_2A_: 7.3 nM, K_D_: rat A_2A_: 8 nM); A_2B_ARs, [^3^H]8-(4-[4-(4-chlorophenyl)piperazine-1-sulfonyl]phenyl)-1-propyl-2,3,6,7-tetrahydro-1*H*-purine-2,6-Dione ([^3^H]PSB-603, Borrmann et al., 2009, 0.3 nM, K_D_ human A_2B_: 0.41 nM); A_3_ARs, [^3^H](*R*)-8-ethyl-4-methyl-2-(phenyl)1,4,7,8-tetrahydro-5*H*-imidazo[2,1-*i*]purin-5-one ([^3^H]PSB-11, Müller et al., 2002, 1 nM, K_D_ human A_3_: 4.9 nM). The radioligands were obtained from Quotient Bioresearch (now Pharmaron): [^3^H]CCPA (58 Ci/mmol), [^3^H]MSX-2 (84 Ci/mmol), [^3^H]PSB-603 (73 Ci/mmol) and [^3^H]PSB-11 (53 Ci/mmol). The non-radioactive precursors of [^3^H]MSX-2, [^3^H]PSB-603 and [^3^H]PSB-11 were synthesized in our laboratory, the precursor for [^3^H]CCPA was synthesized in the laboratory of Gloria Cristalli, University of Camerino, Italy. Membrane preparations and radioligand binding assays at rat A_1_ (rat brain cortex) and rat A_2A_ ARs (rat brain striatum) were performed as previously described (Ozola et al., 2003; Alnouri et al., 2015). For assays at human A_1_, A_2A_, A_2B_, and A_3_ ARs, CHO cell membranes expressing one of the human AR subtypes were used as previously reported (Klotz et al., 1998; Alnouri et al., 2015; De Filippo et al., 2016).

#### Monoamine oxidase assay

The determination of MAO-A and MAO-B inhibition was performed using commercially available recombinant human MAO-A and MAO-B enzymes expressed in baculovirus-infected insects sells (Sigma-Aldrich, M7316 and M7441) applying the commercially available Amplex® Red monoamine oxidase assay kit (Invitrogen A12214). The assays were performed as previously described. The determination of rat MAO-B inhibition was performed using mitochondrial-enriched fractions from male Sprague Dawley rat livers. The assays were conducted as previously described (Stössel et al., 2013).

### Molecular modeling

#### Molecular docking studies at adenosine receptors

The recent co-crystal structures of the human A_1_AR (5N2S.pdb, Cheng et al., 2017) and the A_2A_AR (5N2R.pdb, Cheng et al., 2017) with the antagonist PSB-36 was obtained from the RCSB (Research Collaboratory for Structural Bioinformatics) Protein Data Bank (PDB) (Berman et al., 2000). The downloaded crystal structures were prepared by means of the Molecular Operation Enviroment (MOE 2016.08), chemical computing group. Montreal, Quebec, Canada, 2014) protein structure preparation tool. The hydrogen atoms were assigned according to Protonate-3D implemented in MOE 2016.08. The crystal structures of the human A_1_AR and A_2A_AR were applied for flexible ligand docking using AutoDock 4.2 (Morris et al., 2009). During the docking simulations, the ligands were fully flexible while the residues of the receptor were treated as rigid. Selected compounds were docked into the active site of the receptors to predict the binding modes of the compounds. The atomic partial charges were added using AutoDockTools (Sanner, 1999; Morris et al., 2009). Three-dimensional energy scoring grids for a box of 60 × 60 × 60 points with a spacing of 0.375 Å were computed. The grids were centered based on the co-crystallized ligand, PSB-36. Fifty independent docking calculations using the *var*CPSO-ls algorithm from PSO@Autodock implemented in AutoDock4.2 were performed and terminated after 500,000 evaluation steps (Namasivayam and Günther, 2007). Parameters of *var*CPSO-ls algorithm, the cognitive and social coefficients c1 and c2 were set at 6.05 with 60 individual particles as swarm size. All the other parameters of the algorithm were set at their default values. Possible binding modes of the compounds were explored by visual inspection of the resulting docking poses.

#### Molecular docking studies at monoamine oxidase B

The following programs were used: LigPrep; Maestro; Schrödinger Suites; Schrödinger, LLC: New York, NY, USA, 2017. The X-ray structure of the complex of human MAO-B/safinamide with the accession code 2V5Z was downloaded from the PDB. For the X-ray model, the Protein Preparation Wizard (Schrödinger Inc.) was used in order to add hydrogen atoms, to assign partial charges, and to build missing atoms, side chains and loops. The resulting structures were submitted to energy optimization by using a specific workflow already reported in a previous study (Gidaro et al., 2015). The co-crystallized ligand, safinamide, was used to generate the docking grid box and to check the prediction of the binding affinity. Finally, re-docking simulations were carried out in order to get a protocol validation observing a good capability of the docking software to reproduce the experimental pose of the co-crystallized inhibitor. In standard virtual docking studies, ligands are docked into the binding site of a receptor held as rigid, and the ligand is free to move. However, the assumption of a rigid receptor can give misleading results, since in reality many proteins undergo side-chain or back-bone movements, or both, upon ligand binding. These changes allow the receptor to adapt its binding site to the presence of a certain ligand, a process that is often referred to as the induced fit docking (IFD). This is one of the main complicating factors in structure-based drug design. For that reason, docking studies were carried out by using a specific, previously described IFD workflow (Varela et al., 2012). An initial Glide SP docking of each ligand was performed by using a softened potential, a van der Waals radius scaling factor of 0.50 for receptor/ligand atoms, and a number of 20 poses per ligand to be energy minimized with the OPLS-2005 force field. The poses were saved for each ligand and submitted to the subsequent Prime side chain orientation prediction of residues with a distance cutoff of 5 Å around each ligand. After the Prime energy minimization of the residues and the ligand for each pose, a Glide SP re-docking of each protein/ligand complex structure within 30 kcal/mol above the global minimum was performed. Finally, each output pose was estimated by the binding energy (G-score) and visually examined.

## Results and discussion

### Chemistry

**SCHEME 1:**
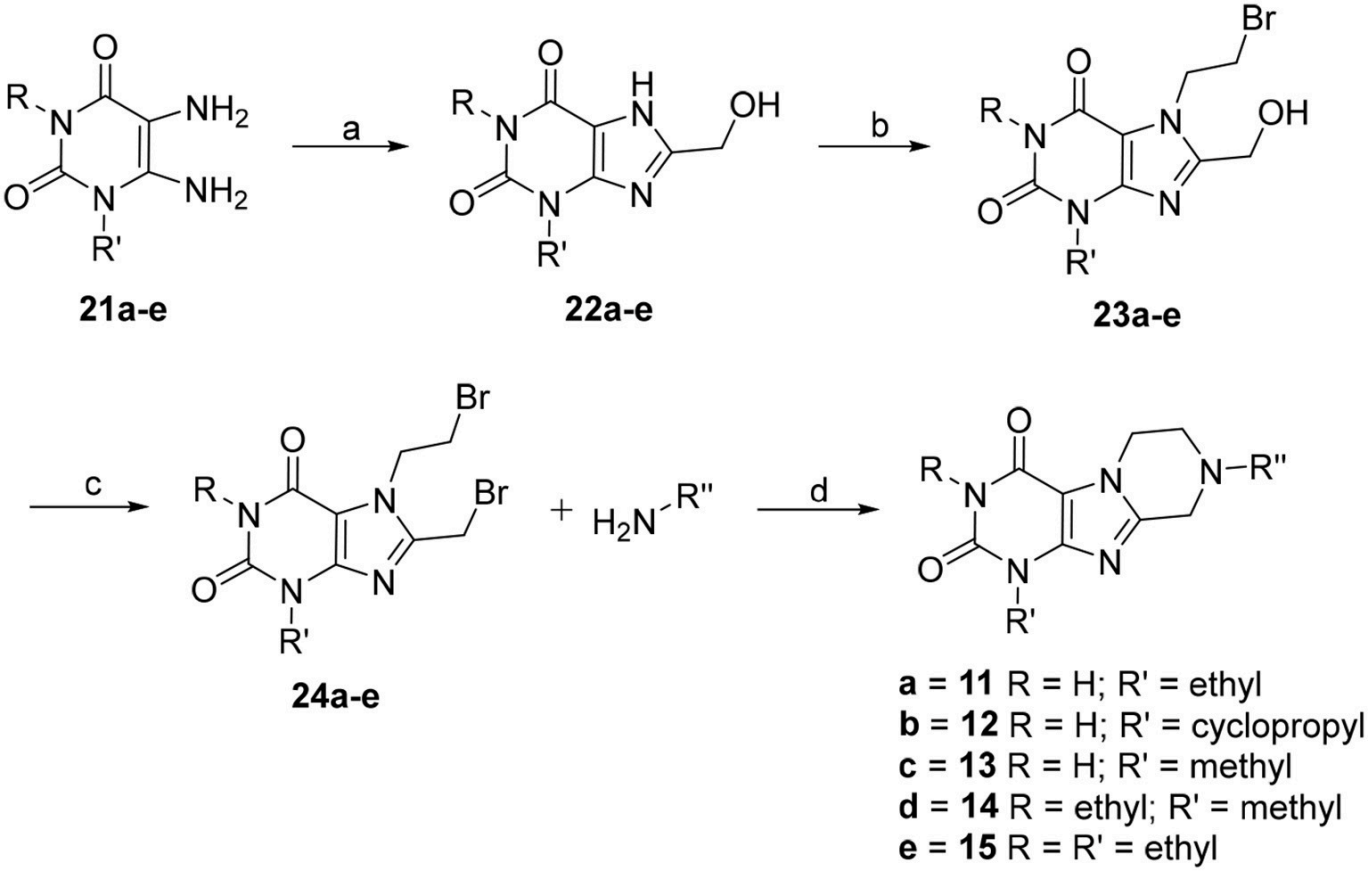
Synthesis of 8-substituted 6,7,8,9-tetrahydropyrazino[2,1-*f* ]purine-2,4(1*H*,3*H*)-diones **11**–**15**. Reagents and conditions: (a) i. glycolic acid (1.2 eq.), 1 h, 100°C, neat, ii. H_2_O, NaOH, pH 12–13, 100°C, 4 h; (b) 1,2-dibromoethane (6 eq.), dimethylformamide (DMF), DIPEA, 70°C, 16 h; (c) PBr_3_ (4 eq.), CH_2_Cl_2_, 0°C to rt, 1 h; (d) amine (2 eq.), dimethoxyethane, DIPEA, rt, 16 h.

The synthetic pathway toward tetrahydropyrazino[2,1-*f* ]purinediones **11**–**15** starting from 5,6-diaminouracil derivatives **21** is depicted in Scheme 1. Compounds **21** were heated together with glycolic acid to 100°C, then brought to pH 12–13 by addition of aqueous NaOH solution, and subsequently heated for 4 h at 100°C to accomplish ring closure reaction yielding **22**. 8-Hydroxymethylxanthines **22** were then alkylated in position 7 by reaction with 1,2-dibromoethane in the presence of diisopropylethylamine (DIPEA). Finally, the hydroxy function of compounds **23** was reacted with PBr_3_, and the resulting 7-(2-bromoethyl)-8-bromomethylpurine-2,4-diones **24** were subsequently treated with different amines under basic conditions yielding the desired tetrahydropyrazino[2,1-*f* ]purinediones **11**–**15**.

**SCHEME 2:**
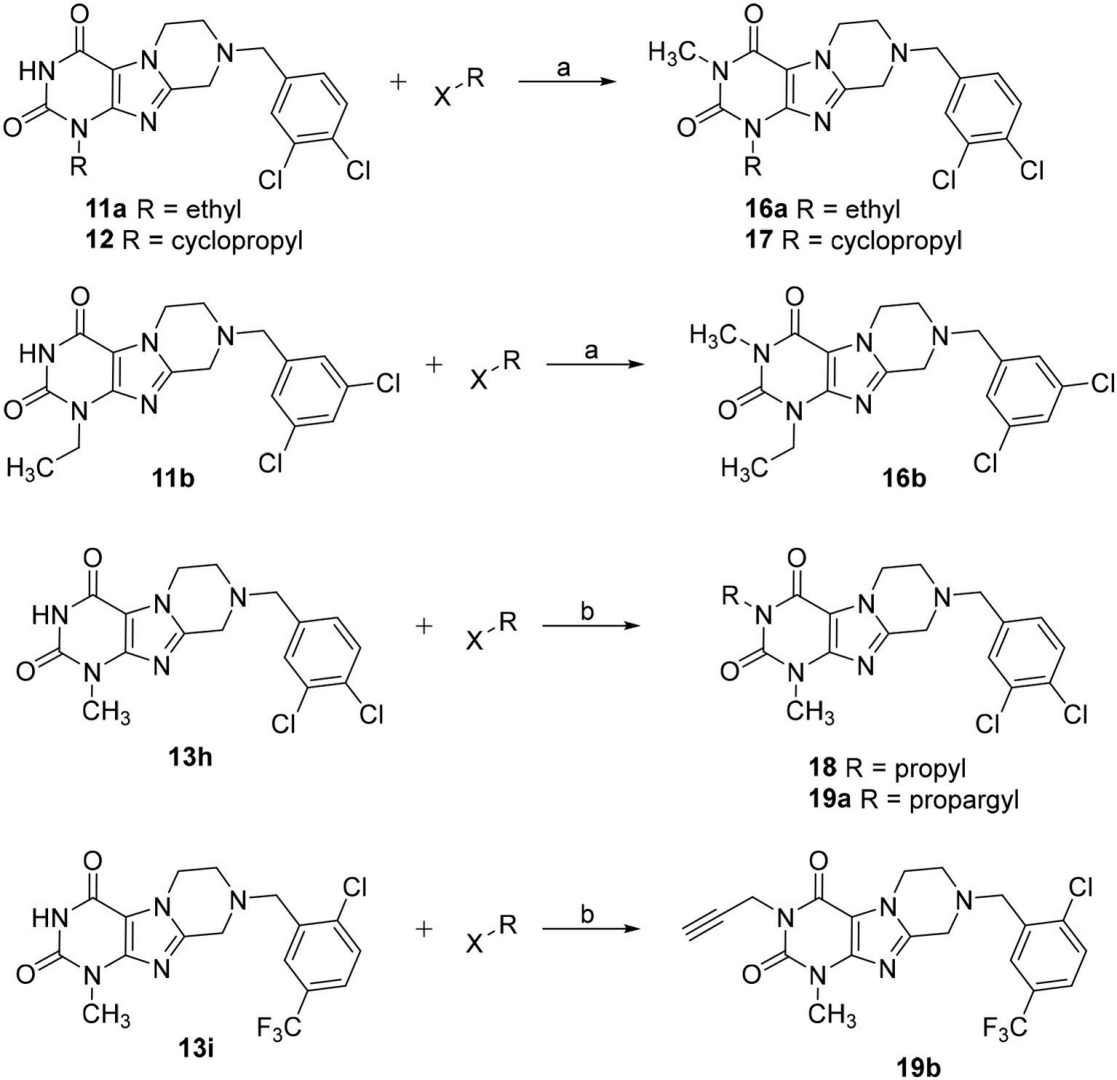
Synthesis of 3-substituted 8-(dichlorobenzyl)-6,7,8,9-tetrahydropyrazino[2,1-*f* ]purine-2,4(1*H*,3*H*)-diones **16**–**18**, **19a** and 8-(2-chloro-5-(trifluoromethyl)benzyl)-1-methyl-3-propargyl-6,7,8,9-tetrahydropyrazino[2,1-*f* ]purine-2,4(1*H*,3*H*)-dione **19b**. Reagents and conditions: (a) MeI (6 eq.), KOtBu (2 eq.), THF, rt, 4 h; (b) alkyl halide (1.1 eq.), NaH (60% in mineral oil) (1.5 eq.), DMF, 8 h, 60°C.

Tetrahydropyrazino[2,1-*f* ]purinediones **16**–**19** were synthesized from the corresponding tetrahydropyrazino[2,1-*f* ]purinediones **11a**, **11b**, **12**, **13h** or **13i** by alkylation of the *N*3-position with the appropriate alkyl halide using sodium *tert*-butoxide or sodium hydride as a base (Scheme 2).

**SCHEME 3:**
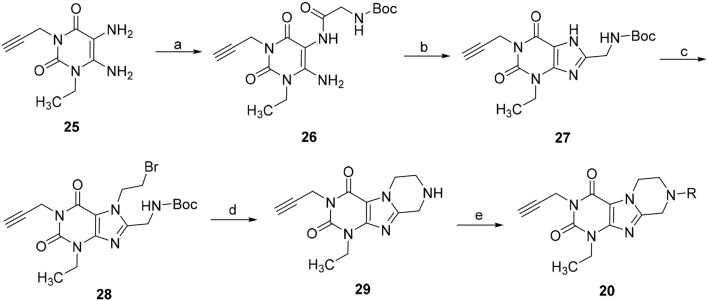
Synthesis of 8-substituted 1-ethyl-3-propargyl-6,7,8,9-tetrahydropyrazino[2,1-*f* ]purine-2,4(1*H*,3*H*)-diones **20**. Reagents and conditions: (a) i. *N*-boc-glycine (1.3 eq.), EDC (1.3 eq.), MeOH, rt, 1 h; (b) aq. 1N-NaOH/dioxane, 100°C, 10 min; (c) 1,2-dibromoethane (6 eq.), DMF, DIPEA, 70°C, 16 h; (d) 4N-HCl in dry dioxane, rt, 0.5 h; (e) 4 eq. DIPEA, dimethoxyethane, rt, 4 h, then R-X (2 eq.), rt, 16 h.

Due to the instability of the propargyl group under bromination conditions, 1-ethyl-3-propargyl-substituted tetrahydropyrazino[2,1-*f* ]purinediones **20** had to be synthesized in a different manner starting from 1-ethyl-3-propargyl-5,6-diaminouracil (**25**) (Scheme 3). Compound **25** was first reacted with *N*-boc-glycine in the presence of 1-ethyl-3-(3-dimethylaminopropyl)carbodiimide (EDC) to form the amide bond, then 1-N NaOH/dioxane solution was added, and the mixture was heated for 10 min at 100°C to accomplish ring closure yielding **27**. The *N*-boc-protected 8-aminomethylxanthine **27** was subsequently alkylated in position 7 by treatment with 1,2-dibromoethane/DIPEA. Finally, the protecting (boc) group was cleaved off under acidic conditions, and subsequent ring closure under basic conditions yielded 1-ethyl-3-propargyl-6,7,8,9-tetrahydropyrazino[2,1-*f* ]purine-2,4(1*H*,3*H*)-dione (**29**). Alkylation of the *N*8-position with different halides resulted in the desired tetrahydropyrazino[2,1-*f* ]purinediones **20**.

The structures of all products were confirmed by nuclear magnetic resonance (^1^H NMR, and in many cases additional ^13^C NMR) and mass spectral analyses. Melting points were determined for all new compounds. The purity of the tested compounds was confirmed by high-performance liquid chromatography (HPLC) coupled to electrospray ionization mass spectrometry (ESI-MS) using two different methods (for details, see Experimental Section) and demonstrated to be generally greater than 95%, except for compounds **20b** and **20k** (purity > 94%).

### Biological evaluation

The synthesized tetrahydropyrazino[2,1-*f* ]purinediones **13**–**20** were evaluated in radioligand binding assays for their affinity to A_1_ ARs of rat brain cortical membrane and to A_2A_ ARs of rat brain striatal membrane preparations. Selected compounds were further investigated for their affinity to human A_1_ and A_2A_ ARs recombinantly expressed in Chinese hamster ovary (CHO) cells. All compounds were additionally investigated for their affinity to human A_2B_ and A_3_ ARs recombinantly expressed in CHO cells to determine their AR subtype selectivity. The following radioligands were employed for radioligand binding studies: [^3^H]2-Chloro-N^6^-cyclopentyladenosine ([^3^H]CCPA, A_1_) (Klotz et al., 1989), [^3^H]3-(3-hydroxypropyl)-8-(*m*-methoxystyryl)-7-methyl-1-propargylxanthine ([^3^H]MSX-2, A_2A_) (Müller et al., 2000), [^3^H]8-(4-(4-(4-chlorophenyl)piperazine-1-sulfonyl)phenyl)-1-propylxanthine ([^3^H]PSB-603, A_2B_) (Borrmann et al., 2009), and [^3^H]2-phenyl-8-ethyl-4-methyl-(8*R*)-4,5,7,8-tetrahydro-1*H*-imidazo[2,1-*i*]purine-5-one ([^3^H]PSB-11, A_3_) (Müller et al., 2002). It is well known that all xanthine derivatives lacking a ribose moiety, including tricyclic compounds, can only block ARs, but never act as AR agonists; therefore, additional functional studies were not required. All compounds were initially tested for inhibition of human MAO-B at a concentration of 10 μM. For compounds that showed an inhibition of greater than 70% full concentration-inhibition curves were recorded and IC_50_ values were determined. Potent MAO-B inhibitors were additionally investigated for inhibition of human MAO-A to assess their selectivity. Results are presented in Tables 1–6, and data of standard ligands are included for comparison.

**Table 1:** Adenosine receptor affinities of 3-ethyl-1-methyltetrahydropyrazino[2,1-*f* ]purinediones **14** and standard antagonists.

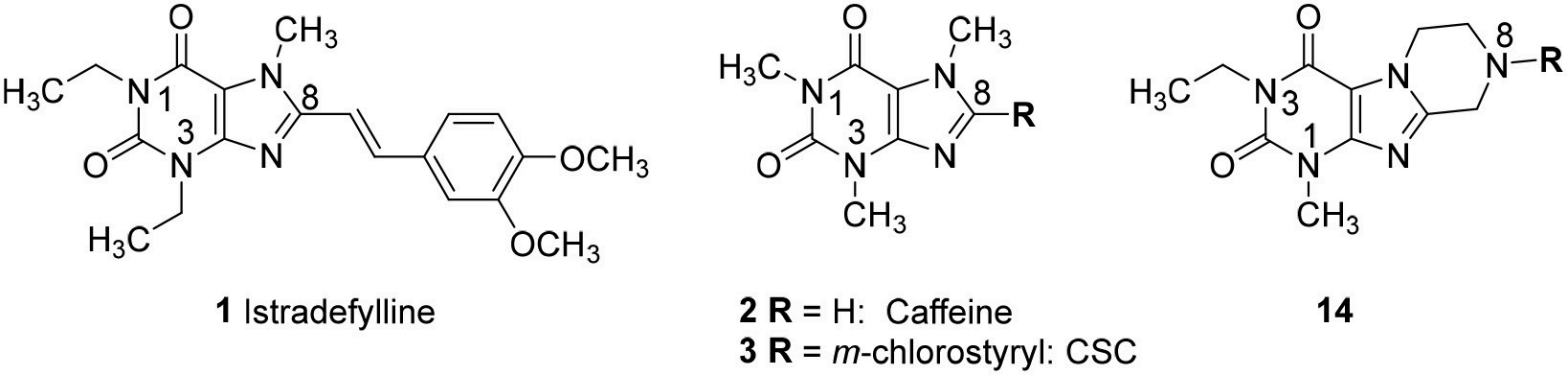					
		**K**_i_ ± **SEM (nM) human (h); rat (r)**			
**Compd**	**R**	**A_1_ vs. [^3^H]CCPA^a^**	**A_2A_ vs. [^3^H]MSX-2^a^**	**A_2B_ vs. [^3^H]PSB-603^b^**	**A_3_ vs. [^3^H]PSB-11^b^**
Istradefylline (**1**)^c^		841 (**h**)	12 (**h**)	>10,000 (**h**)	4,470 (**h**)
		230 (**r**)	4.46 (**r**)		
Caffeine (**2**)^c^		44,900 (**h**)	23,400 (**h**)	33,800 (**h**)	13,300 (**h**)
		41,000 (**r**)	32,500 (**r**)	30,000 (**r**)	>10,000 (**r**)
CSC (**3**)^c^		>10,000 (14%)^d^ (**h**)	38.0 ± 11.0 (**h**)	8,200 (**h**)	>10,000 (**h**)
		28,000 (**r**)	54 (**r**)		
**3-ETHYL-1-METHYLTETRAHYDROPYRAZINO[2,1-*****f*****]PURINEDIONES 14**					
**14a**	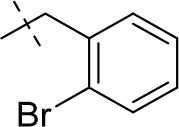	41.7 ± 8.3 (**h**)	497 ± 62 (**h**)	>1,000 (**h**) (18%)^d^	>10,000 (**h**) (36%)^d^
		123 ± 34 (**r**)	408 ± 90 (**r**)		
**14b**	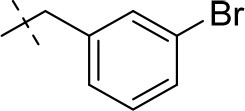	70.7 ± 12.3 (**h**)		>1,000 (**h**) (9%)^d^	>10,000 (**h**) (39%)^d^
		28.8 ± 1.5 (**r**)	1,140 ± 160 (**r**)		
**14c**	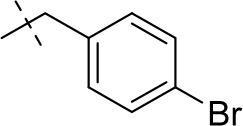	1,350 ± 340 (**h**)		>1,000 (**h**) (1%)^d^	9,390 ± 1,830 (**h**)^a^
		156 ± 21 (**r**)	>1,000 (**r**) (35%)^d^		
**14d**	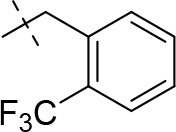	244 ± 74 (**h**)		>1,000 (**h**) (2%)^d^	8,760 ± 930 (**h**)^a^
		114 ± 15 (**r**)	>1,000 (**r**) (20%)^d^		
**14e**	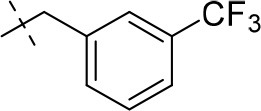	156 ± 49 (**h**)	1,780 ± 770 (**h**)	>100 (**h**) (2%)^d^	>10,000 (**h**) (38%)^d^
		49.1 ± 6.2 (**r**)	708 ± 25 (**r**)		
**14f**	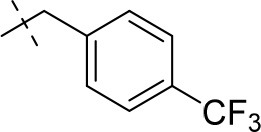			>100 (**h**) (1%)^d^	18,600 ± 2,800 (**h**)^a^
		>1,500 (**r**) (9%)^d^	>1,000 (**r**) (7%)^d^		
**14g**	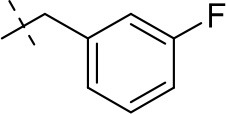	85.3 ± 5.5 (**h**)		>100 (**h**) (15%)^d^	>10,000 (**h**) (24%)^d^
		59.4 ± 7.7 (**r**)	>1,000 (**r**) (18%)^d^		
**14h**	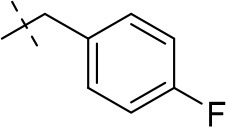	5,120 ± 150 (**h**)		>1,000 (**h**) (9%)^d^	>10,000 (**h**) (37%)^d^
		164 ± 28 (**r**)	>1,000 (**r**) (7%)^d^		
**14i**	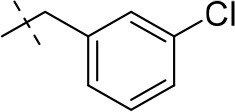	121 ± 45 (**h**)	>1,000 (**h**) (8%)^d^	>10,000 (**h**) (36%)^d^	
		53.0 ± 5.8 (**r**)	>1,000 (**r**) (33%)^d^		
**14j**	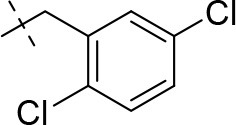	391 ± 77 (**h**)	906 ± 73 (**h**)	>1,000 (**h**) (2%)^d^	>10,000 (**h**) (42%)^d^
		36.9 ± 6.1 (**r**)	617 ± 114 (**r**)		
**14k**	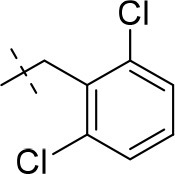	197 ± 5 (**h**)		>1,000 (**h**) (8%)^d^	>10,000 (**h**) (17%)^d^
		142 ± 32 (**r**)	1,100 ± 380 (**r**)		
**14l**	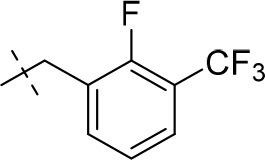	495 ± 80 (**h**)	2,020 ± 480 (**h**)	>1,000 (**h**) (8%)^d^	>10,000 (**h**) (29%)^d^
		236 ± 57 (**r**)	667 ± 68 (**r**)		
**14m**	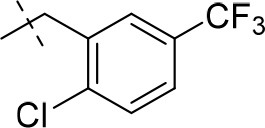	55.9 ± 8.3 (**h**)	881 ± 96 (**h**)	>1,000 (**h**) (3%)^d^	>10,000 (**h**) (35%)^d^
		76.2 ± 9.0 (**r**)	592 ± 28 (**r**)		
**14n**	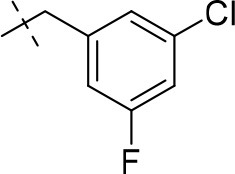	298 ± 52 (**h**)		>1,000 (**h**) (1%)^d^	15,600 ± 4,000 (**h**)^a^
		36.2 ± 9.9 (**r**)	981 ± 116 (**r**)		
**14o**	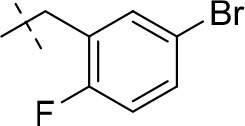	116 ± 27 (**h**)	1,700 ± 150 (**h**)	>1,000 (**h**) (11%)^d^	>10,000 (**h**) (39%)^d^
		36.0 ± 18.4 (**r**)	880 ± 174 (**r**)		
**14p**	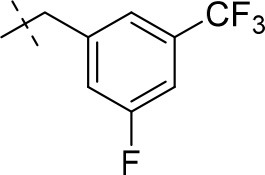	330 ± 67 (**h**)	503 ± 121 (**h**)	>1,000 (**h**) (5%)^d^	>10,000 (**h**) (39%)^d^
		111 ± 25 (**r**)	801 ± 99 (**r**)		
**14q**	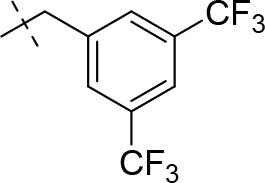			>1,000 (**h**) (2%)^d^	>10,000 (**h**) (32%)^d^
		>1,500 (**r**) (31%)^d^	>1,000 (**r**) (39%)^d^		

a *n = 3*.

b *n = 2*.

c *data taken from Müller and Jacobson (2011); Brunschweiger et al. (2014)*.

d *% inhibition of radioligand binding at indicated concentration*.

#### Structure-activity relationships at adenosine receptors

It should be noted that the N1 of xanthines corresponds to the N3 of pyrazino[2,1-*f* ]purinediones and vice versa (see Table 1). Within the series of the N8-benzyl-substituted 3-ethyl-1-methyltetrahydropyrazino[2,1-*f* ]purinediones **14** several potent A_1_ AR antagonists and dual A_1_/A_2A_ AR antagonists showing K_i_ values down to the double-digit nanomolar range were identified (Table 1). As a general trend within this series, all compounds showed a preference for the A_1_ vs. the A_2A_ AR at both the human and the rat ARs. None of the compounds out of this series showed any significant binding to the human A_2B_ AR. Two derivatives displayed low affinity for the A_3_ ARs (**14c**, K_i_ = 9,390 nM and **14d** K_i_ = 8,760 nM).

The N8-(2-bromobenzyl)-substituted compound **14a** was found to be a potent dual A_1_/A_2A_ AR antagonist displaying >10-fold selectivity for the human A_1_ vs. the human A_2A_ AR (K_i_, A_1_ AR = 41.7 nM; K_i_, A_2A_ AR = 497 nM). Species differences between the human and rat A_1_ AR were most prominent in case of the *para*-substituted benzyl derivatives. Compounds **14c** and **14h** were significantly more potent at the rat A_1_ AR as compared to the human A_1_ AR. Further derivatives, **14j**-**14q**, bearing a disubstituted benzyl moiety at position N8 were designed, which are lacking a substituent in the *para*-position. A 2,3-, 3,4- or 3,5-disubstitution pattern on the benzene ring was well tolerated by the A_1_ AR and also improved in most cases the affinity for the A_2A_ AR. However, compound **14q** bearing two larger CF_3_-groups in positions 3 and 5 was nearly inactive.

Compound **14m** having a 2-Cl-5-CF_3_-benzyl moiety at position N8 of the tetrahydropyrazino[2,1-*f* ]purinedione core was found to be a potent A_1_ AR antagonist at both rat and human ARs (K_i_ human A_1_ = 55.9 nM; K_i_ rat A_1_ = 76.2 nM) and displayed a 16-fold selectivity for the (human) A_1_ vs. the A_2A_ AR.

None of the designed compounds lacking a substituent at the N3-position of the tricyclic purinedione core (series **13**)—which corresponds to the xanthine N1 position—displayed any significant affinity for ARs (Table 2). The presence of a substituent at that position appeared to be essential for blocking A_1_ and A_2A_ ARs, e.g., compare 1-methyltetrahydropyrazino[2,1-*f* ]-purinedione derivative **13h** with its 1,3-dimethyltetrahydropyrazino[2,1-*f* ]-purinedione derivative **10b**, or compare **13a** and **13b** with **14a** and **14e**, respectively.

**Table 2:** Adenosine receptor affinities of 1-methyltetrahydropyrazino[2,1-*f* ]purinediones **13**.

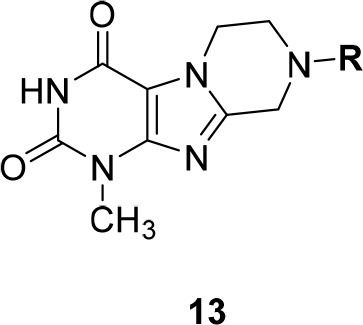					
		**K**_i_ ± **SEM (nM) human (h); rat (r)**			
**Compd**	**R**	**A_1_ vs. [^3^H]CCPA^a^**	**A_2A_ vs. [^3^H]MSX-2^a^**	**A_2B_ vs. [^3^H]PSB-603^b^**	**A_3_ vs. [^3^H]PSB-11^b^**
**13a**	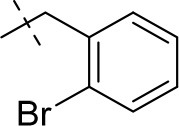			>1,000 (**h**) (1%)^c^	14,700 ± 4,700 (**h**)^a^
		>1,500 (**r**) (17%)^c^	>1,000 (**r**) (-23%)^c^		
**13b**	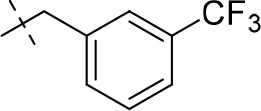			>1,000 (**h**) (2%)^c^	>10,000 (**h**) (35%)^c^
		>1,500 (**r**) (−1%)^c^	>1,000 (**r**) (5%)^c^		
**13c**	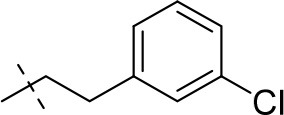			>1,000 (**h**) (18%)^c^	>10,000 (**h**) (49%)^c^
		>1,500 (**r**) (3%)^c^	>1,000 (**r**) (1%)^c^		
**13d**	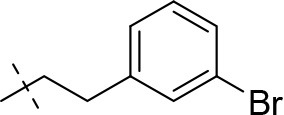			>1,000 (**h**) (1%)^c^	ca. 10,000 (**h**) (45%)^c^
		>1,500 (**r**) (1%)^c^	>1,000 (**r**) (15%)^c^		
**13e**	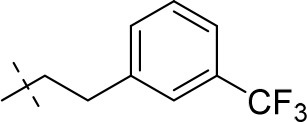			>300 (**h**) (8%)^c^	>10,000 (**h**) (39%)^c^
		>1,500 (**r**) (9%)^c^	>1,000 (**r**) (4%)^c^		
**13f**	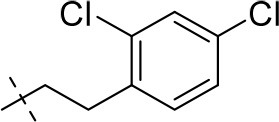			>1,000 (**h**) (1%)^c^	17,200 ± 4,500 (**h**)^a^
		>1,500 (**r**) (5%)^c^	>1,000 (**r**) (19%)^c^		
**13g**	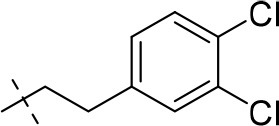			>1,000 (**h**) (1%)^c^	>10,000 (**h**) (41%)^c^
		>1,500 (**r**) (3%)^c^	>1,000 (**r**) (10%)^c^		
**13h**	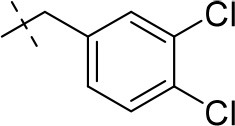			>1,000 (**h**) (3%)^c^	>1,000 (**h**) (14%)*^c^*
		>1,500 (**r**) (36%)^c^	>1,000 (**r**) (−9%)^c^		

a *n = 3*.

b *n = 2*.

c *% inhibition of radioligand binding at indicated concentration*.

Similar to series **14**, all compounds of the N1,N3-diethyl-substituted tetrahydropyrazino[2,1-*f* ]purinedione series **15** displayed higher affinity for the A_1_ than for the A_2A_ AR subtype (Table 3). Comparison of **15b** with **14g** as well as **15c** with **14i** reveals that elongation of the substituent in the N1-position from methyl (series **14**) to ethyl (series **15**) resulted in an increase in affinity for the A_3_ AR. The *m*-bromobenzyl derivative **15d** was found to be a very potent and selective antagonist of the human and rat A_1_ AR (K_i_, human A_1_ AR = 13.6 nM; K_i_, rat A_1_ AR = 21.5 nM). Introduction of a second substituent (fluorine atom) at the benzyl moiety of **15d** (compounds **15k** and **15l**) resulted in a decrease in both A_1_ AR affinity and selectivity vs. the A_2A_ AR. Within the examples having a disubstituted 8-benzyl moiety, compounds having a trifluoromethyl substituent in *meta*-position and second substituent in *para*- or *meta*-position (**15i**, **15p**, **15q**) show affinity for the rat A_2A_ AR. However, the A_1_ AR tolerates a *m*-trifluoromethyl,*p*-chloro substitution pattern at the 8-benzyl moiety (**15p**).

**Table 3:** Adenosine receptor affinity of 1,3-diethyltetrahydropyrazino[2,1-*f* ]purinediones **15**.

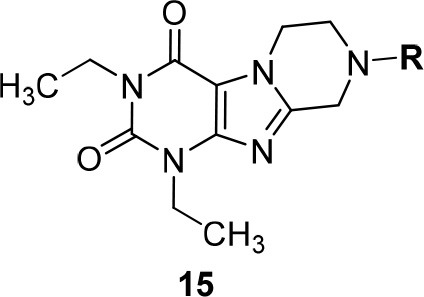					
		**K**_i_ ± **SEM (nM) human (h); rat (r)**			
**Compd**	**R**	**A_1_ vs. [^3^H]CCPA^a^**	**A_2A_ vs. [^3^H]MSX-2^a^**	**A_2B_ vs. [^3^H]PSB-603^b^**	**A_3_ vs. [^3^H]PSB-11^b^**
**15a**	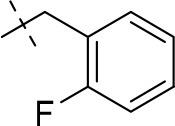	156 ± 5 (**h**)		>1,000 (**h**) (12%)^c^	7,820 ± 1,240 (**h**)^a^
		32.0 ± 0.9 (**r**)	2,000 ± 430 (**r**)		
**15b**	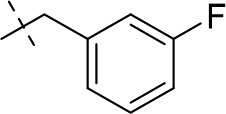	207 ± 11 (**h**)		>1,000 (**h**) (9%)^c^	7,020 ± 870 (**h**)^a^
		47.2 ± 19.9 (**r**)	1,580 ± 80 (**r**)		
**15c**	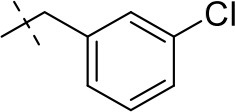	128 ± 17 (**h**)	2,380 ± 70 (**h**)	>1,000 (**h**) (12%)^c^	5,410 ± 850 (**h**)^a^
		85.9 ± 28.6 (**r**)	>1,000 (**r**) (22%)^c^		
**15d (PSB-18339)**	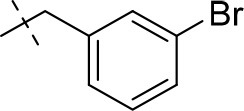	13.6 ± 2.1 (**h**)	1,050 ± 300 (**h**)	496 ± 135 (**h**)^a^	7,220 ± 1,170 (**h**)^a^
		21.5 ± 8.3 (**r**)	1,040 ± 90 (**r**)		
**15e**	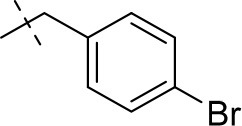		4,420 ± 1,170 (**h**)	>1,000 (**h**) (39%)^c^	>1,000 (**h**) (12%)^c^
		54.0 ± 9.1 (**r**)	1,140 ± 290 (**r**)		
**15f**	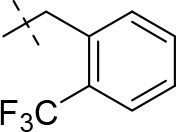	129 ± 9 (**h**)		>1,000 (**h**) (2%)^c^	4,060 ± 930 (**h**)^a^
		51.4 ± 14.7 (**r**)	>1,000 (**r**) (32%)^c^		
**15g**	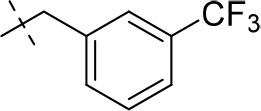	84.8 ± 2.4 (**h**)	277 ± 59 (**h**)	>1,000 (**h**) (27%)^c^	>10,000 (**h**) (36%)^c^
		30.0 ± 5.7 (**r**)	1,030 ± 130 (**r**)		
**15h**	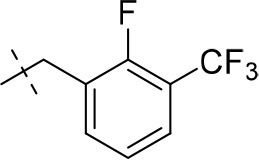	189 ± 29 (**h**)	663 ± 54 (**h**)	>1,000 (**h**) (30%)^c^	>10,000 (**h**) (29%)^c^
		172 ± 28 (**r**)	895 ± 210 (**r**)		
**15i**	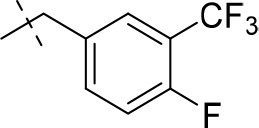			>1,000 (**h**) (33%)^c^	>10,000 (**h**) (36%)^c^
		>1,500 (**r**) (19%)^c^	>1,000 (**r**) (30%)^c^	
**15j**	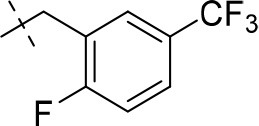	39.4 ± 7.4 (**h**)	781 ± 68 (**h**)	>1,000 (**h**) (27%)^c^	>10,000 (**h**) (25%)^c^
		124 ± 21 (**r**)	600 ± 54 (**r**)		
**15k**	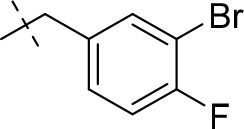	362 ± 98 (**h**)	756 ± 271 (**h**)	>1,000 (**h**) (34%)^c^	7,190 ± 2,200 (**h**)^a^
		270 ± 60 (**r**)	>1,000 (**r**) (30%)^c^		
**15l**	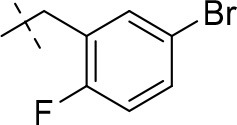	75.4 ± 16.1 (**h**)	598 ± 92 (**h**)	>1,000 (**h**) (24%)^c^	13,300 ± 4,300 (**h**)^a^
		33.5 ± 5.6 (**r**)	756 ± 46 (**r**)		
**15m**	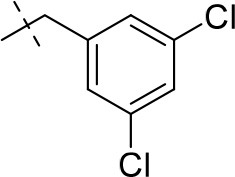	207 ± 48 (**h**)	631 ± 122 (**h**)	>1,000 (**h**) (23%)^c^	4,720 ± 720 (**h**)^a^
		89.6 ± 21.4 (**r**)	2,200 ± 180 (**r**)		
**15n**	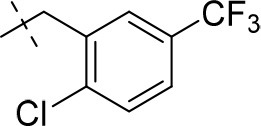	22.6 ± 2.7 (**h**)	613 ± 34 (**h**)	>1,000 (**h**) (23%)^c^	>10,000 (**h**) (38%)^c^
		19.6 ± 3.0 (**r**)	871 ± 50 (**r**)		
**15o**	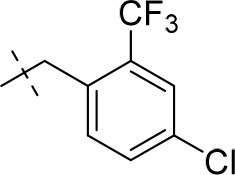			>1,000 (**h**) (16%)^c^	1,690 ± 320 (**h**)^c^
		>1,500 (**r**) (41%)^c^	>1,000 (**r**) (19%)^c^
**15p**	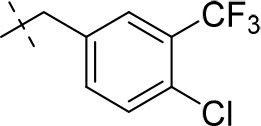	129 ± 22 (**h**)		>1,000 (**h**) (32%)^c^	7,180 ± 860 (**h**)^c^
		588 ± 28 (**r**)	>1,000 (**r**) (40%)^c^		
**15q**	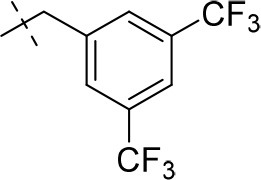			>1,000 (**h**) (7%)^c^	>10,000 (**h**) (40%)^c^
		>1,500 (**r**) (6%)^c^	>1,000 (**r**) (13%)^c^		
**15r**	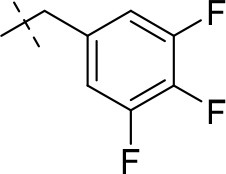	1,190 ± 27 (**h**)	4,110 ± 890 (**h**)	>1,000 (**h**) (23%)^c^	>10,000 (**h**) (14%)^c^
		>1,500 (**r**) (25%)^c^	>1,000 (**r**) (36%)^c^		

a *n = 3*.

b *n = 2*.

c *% inhibition of radioligand binding at indicated concentration*.

Within the 1-ethyl-3-propargyltetrahydropyrazino[2,1-*f* ]purinedione series **20**, benzyl derivative **20e** was found to be a balanced dual A_1_/A_2A_ AR antagonist with good potency in both species, rat and human (Table 4). Introduction of a methoxy group in the *ortho*-position in **20e** led to a similarly potent dual A_1_/A_2A_ antagonist in humans (**20f**, K_i_ A_1_ = 210 nM; K_i_ A_2A_ = 311 nM) but showed larger species differences in rat. N8-Phenethyl-substituted tetrahydropyrazinopurinedione **20h** was most potent and selective A_2A_ AR of this series in humans (K_i_ hA_2A_ = 149 nM). In case of the rat receptor, the opposite results were observed. Compound **20h** displayed a lower K_i_ value for the A_1_ AR than for A_2A_ AR (K_i_ A_2A_ = 1,700 nM; K_i_ A_1_ = 117 nM). A methoxy group in the *para*-position of the phenethyl ring (**20k**, **20l**) led to reduced affinities at both rat adenosine receptor subtypes.

**Table 4:** Adenosine receptor affinity of 1-ethyl-3-propargyltetrahydropyrazino[2,1-*f* ]purinediones **20**.

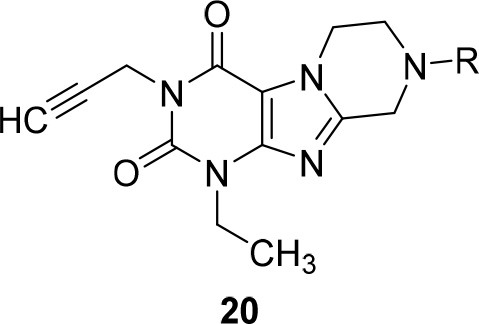					
		**K**_i_ ± **SEM (nM) human (h); rat (r)**			
**Compd**	**R**	**A_1_ vs. [^3^H]CCPA^a^**	**[^3^H]MSX-2^a^**	**A_2B_ vs.[^3^H]PSB-603^b^**	**A_3_ vs. [^3^H]PSB-11^b^**
**20a**	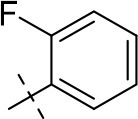			>1,000 (**h**) (8%)^c^	4,270 ± 890 (**h**)^a^
		1,140 ± 260 (**r**)	1,380 ± 260 (**r**)		
**20b**	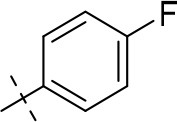	1,530 ± 180 (**h**)	378 ± 49 (**h**)	>1,000 (**h**) (15%)^c^	2,390 ± 340 (**h**)^a^
		410 ± 60 (**r**)	128 ± 20 (**r**)		
**20c**	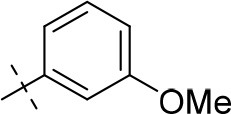	946 ± 166 (**h**)	289 ± 85 (**h**)	>1,000 (**h**) (38%)^c^	7,160 ± 950 (**h**)^a^
		328 ± 70 (**r**)	250 ± 80 (**r**)		
**20d**	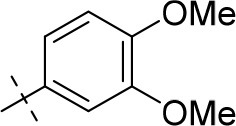	1,670 ± 320 (**r**)	1,580 ± 340 (**r**)	>1,000 (**h**) (8%)^c^	3,640 ± 720 (**h**)^a^
**20e (PSB-1869)**	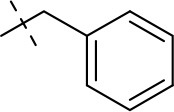	180 ± 28 (**h**)	282 ± 38 (**h**)	>1,000 (**h**) (2%)^c^	>10,000 (**h**) (35%)^c^
		118 ± 28 (**r**)	245 ± 39 (**r**)		
**20f**	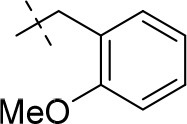	210 ± 90 (**h**)	311 ± 106 (**h**)	>1,000 (**h**) (18%)^c^	7,640 ± 700 (**h**)^a^
		32.7 ± 5.8 (**r**)	1,200 ± 140 (**r**)		
**20g**	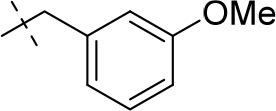	124 ± 90 (**h**)	542 ± 169 (**h**)	>1,000 (**h**) (22%)^c^	>10,000 (**h**) (40%)^c^
		118 ± 22 (**r**)	769 ± 77 (**r**)		
**20h**	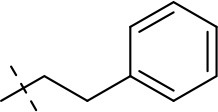	2,440 ± 400 (**h**)	149 ± 28 (**h**)	>1,000 (**h**) (16%)^c^	>10,000 (**h**) (36%)^c^
		117 ± 2 (**r**)	1,700 ± 160 (**r**)		
**20i**	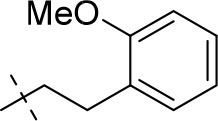	1,260 ± 250 (**h**)	3,230 ± 730 (**h**)	>1,000 (**h**) (24%)^c^	5,830 ± 370 (**h**)^a^
		701 ± 204 (**r**)	883 ± 265 (**r**)		
**20j**	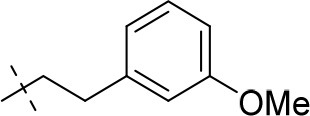	1,020 ± 350 (**h**)	25,500 ± 5,400 (**h**)	>1,000 (**h**) (11%)^c^	>10,000 (**h**) (13%)^c^
		669 ± 35 (**r**)	798 ± 134 (**r**)		
**20k**	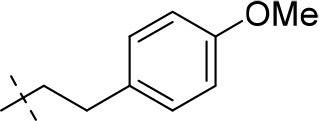	1,600 ± 610 (**r**)	2,700 ± 300 (**r**)	>1,000 (**h**) (10%)^c^	>10,000 (**h**) (22%)^c^
**20l**	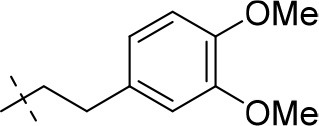	3,800 ± 330 (**r**)	7,400 ± 220 (**r**)	>1,000 (**h**) (9%)^c^	>10,000 (**h**) (12%)^c^

a *n = 3*.

b *n = 2*.

c *% inhibition of radioligand binding at indicated concentration*.

In the last series of compounds (**16**–**19**), the influence of ethyl and cyclopropyl at the N1-position as well as ethyl and propargyl at the N3-position was probed (Table 5). These were combined with a 3,4-dichlorobenzyl substituent at N8 as in lead structure **10b** (1,3-dimethyl-substituted analog, see Figure 3). Compound **16a** was found to be a potent dual A_1_/A_2A_ AR antagonists with ancillary MAO-B inhibitory activity, similarly to **10b**. Switching from a 3,4-dichloro substitution pattern (**16a**) of the benzyl moiety to 3,5-dichloro substitution (in **16b**) resulted in an improvement in affinity for both human and rat A_1_ AR. Compared to **16a**, a cyclopropyl moiety at the N1-position (compound **17**) was less tolerated. A propargyl substituent at N3 combined with a 3,4-dichlorobenzyl at position N8 (**19a**) somewhat improved the affinity for the human A_3_ AR. Changing the substitution pattern on the benzyl ring from 3,4-dichloro to 2-chloro-5-trifluoromethyl (in **19b**) eliminated the affinity for the A_3_ AR and increased the affinity for the A_1_/A_2A_ ARs.

**Table 5:** Adenosine receptor affinities of 1-ethyl-3-methyltetrahydropyrazino[2,1-*f* ]purinediones **16**, 1-cyclopropyl-3-methyltetrahydropyrazino[2,1-*f* ]purinedione **17**, 1-methyl-3-propyltetrahydropyra-zino[2,1-*f* ]purinedione **18** and 1-methyl-3-propargyltetrahydropyrazino[2,1-*f* ]purinediones **19**.

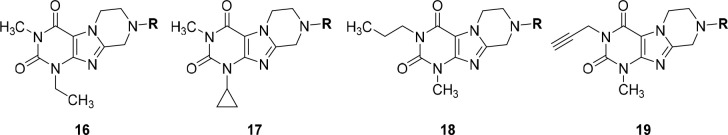					
		**K**_i_ ± **SEM (nM) human (h); rat (r)**			
**Compd**	**R**	**A_1_ vs. [^3^H]CCPA^a^**	**A_2A_ vs.[^3^H]MSX-2^a^**	**A_2B_ vs. [^3^H]PSB-603^b^**	**A_3_ vs. [^3^H]PSB-11^b^**
**1-ETHYL-3-METHYLTETRAHYDROPYRAZINO[2,1-*****f*****]PURINEDIONES 16**					
**16a (PSB-18405)**	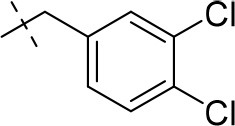	396 ± 154 (**h**)	1,620 ± 280 (**h**)	>1,000 (**h**) (5%)^c^	>10,000 (**h**) (19%)^c^
		236 ± 23 (**r**)	1,100 ± 390 (**r**)		
**16b**	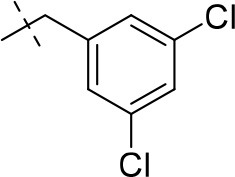	143 ± 16 (**h**)	2,230 ± 640 (**h**)	>1,000 (**h**) (5%)^c^	>10,000 (**h**) (23%)^c^
		49.3 ± 8.0 (**r**)	1,490 ± 270 (**r**)		
**1-CYCLOPROPYL-3-METHYLTETRAHYDROPYRAZINO[2,1-*****f*****]PURINEDIONE 17**					
**17**	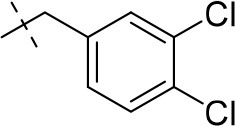	1,130 ± 180 (**h**)		>100 (**h**) (2%)^c^	>10,000 (**h**) (26%)^c^
		584 ± 8 (**r**)	3,940 ± 390 (**r**)		
**1-METHYL-3-PROPYLTETRAHYDROPYRAZINO[2,1-*****f*****]PURINEDIONE 18**					
**18**	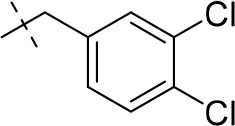	475 ± 47 (**h**)	2,110 ± 370 (**h**)	>1,000 (**r**) (33%)^c^	12,700 ± 2,500 (**h**)^a^
		152 ± 12 (**r**)	675 ± 104 (**r**)		
**1-METHYL-3-PROPARGYLTETRAHYDROPYRAZINO[2,1-*****f*****]PURINEDIONES 19**					
**19a**	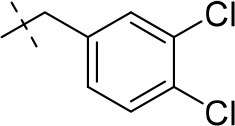	967 ± 211 (**h**)	1,580 ± 550 (**h**)	>1,000 (**h**) (23%)^c^	5,630 ± 210 (**h**)^a^
		292 ± 21 (**r**)	642 ± 194 (**r**)		
**19b**	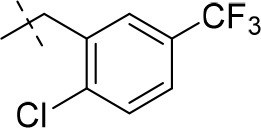	110 ± 4 (**h**)	712 ± 129 (**h**)	>1,000 (**h**) (3%)^c^	>10,000 (**h**) (25%)^c^
		153 ± 10 (**r**)	414 ± 49 (**r**)		

a *n = 3*.

b n = 2

c *% inhibition of radioligand binding at indicated concentration*.

#### Docking studies at A_1_ and A_2A_ adenosine receptors

**Figure 5:**
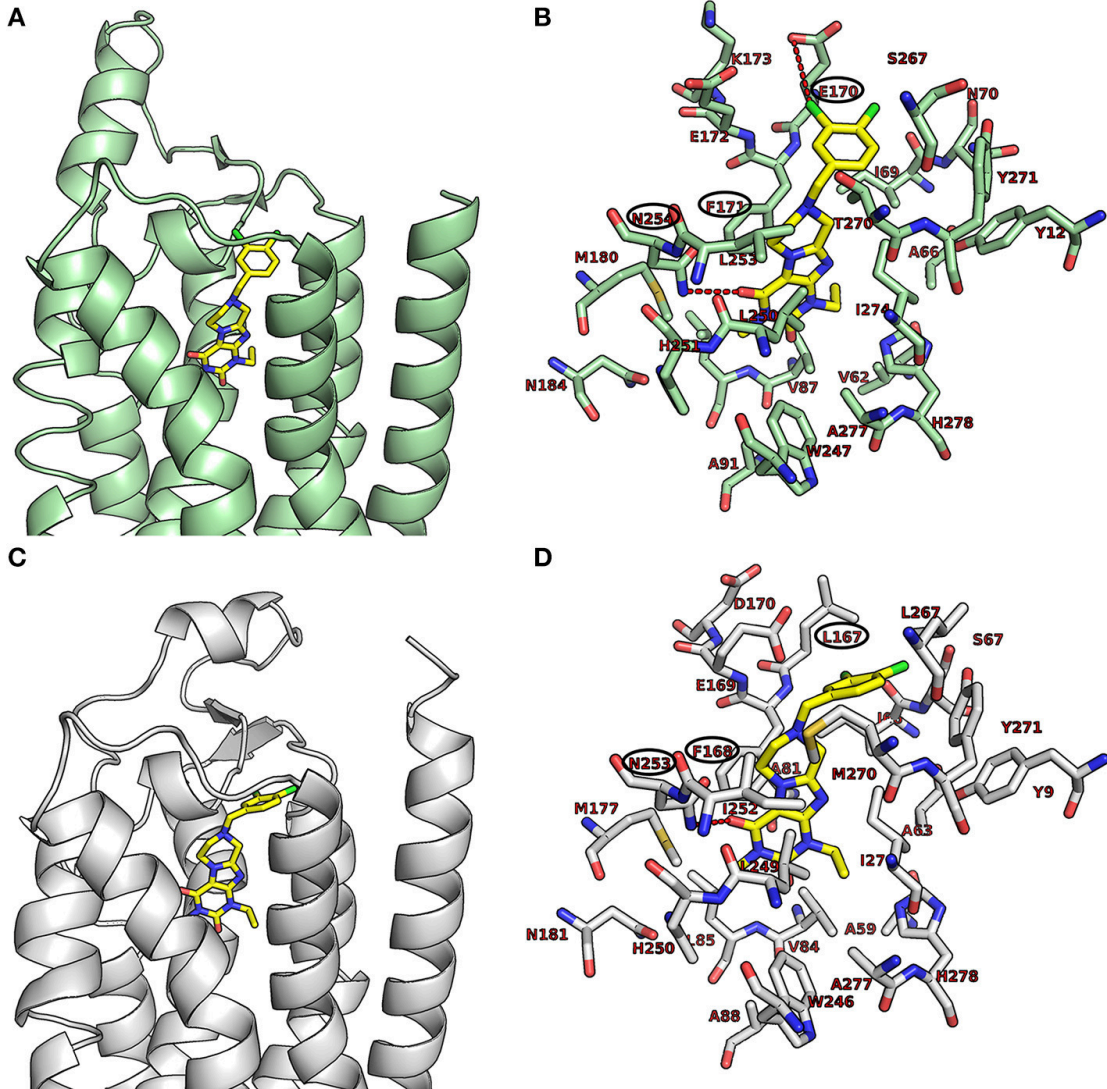
Putative binding pose of **16a**. **(A)** Docked pose of **16a** (yellow) in the binding pocket of the human A_1_ AR; **(B)** shows important amino acids in the binding pocket that are believed to interact with **16a**. **(C)** Docked pose of **16a** (yellow) in the binding pocket of the human A_2A_ AR; **(D)** shows important amino acids in the binding pocket that likely interact with **16a**. The human A_1_ AR (pale green) and the human A_2A_ AR (gray) are displayed in the cartoon representation, the important amino acids as stick model. Oxygen atoms are colored in red, nitrogen atoms in blue, and sulfur in yellow. The interactions are indicated by red dotted lines, and amino acids found to be responsible for the key interactions are encircled in black.

As shown in Figures 5A,B, the purinedione core structure of the dual A_1_/A_2A_ AR antagonist **16a** forms one of the key π-π stacking interactions with Phe171 and utilizes the hydrophobic surface provided by Leu250 in the human A_1_ AR. The carbonyl group at position C4 which corresponds to the C6-carbonyl of xanthine forms another key hydrogen bond interaction with Asn254. The methyl substituent at N1 (corresponding to N3 of xanthine) of purinedione derivative **16a** binds within the hydrophobic sub-pocket formed by the residues Leu88, Met180 and Leu250. Similarly, the ethyl substituent at N3 binds in another sub-pocket formed by Ala66, Ile69, Val87, Ile274, and His278. The 3,4-dichlorobenzyl substitution at N8 was found to occupy the pocket formed by the residues Tyr12, Ile69, Asn70, Glu170, Glu172, Ser267, and Tyr271. The tetrahydropyrazine ring which is annelated to the xanthine core, and the methylene group in the benzyl moiety direct the aromatic substituent into a specific binding pocket. A possible electrostatic interaction between the chloro substituent at the 3-position of the benzyl moiety and Glu170 may be beneficial for interaction with the human A_1_AR. This was supported by the observed high affinity of compound **16b** with 3,5-dichloro substitution, a modification which possibly increases the chance to form interactions with Glu170. In comparison to classical 8-substituted xanthine derivatives such as PSB-36 found in recently published X-ray structures (Cheng et al., 2017), compound **16a** and related compounds featuring a tricyclic core structure are somewhat less potent possibly due to the loss of the free N7-H in xanthines, which forms interactions with Asn254 or water-mediated interactions with Glu172.

As shown in Figures 5C,D, compound **16a** follows a similar interaction pattern in the human A_2A_ AR for the purinedione core forming key hydrophobic and hydrogen bond interactions with Phe168 and Asn253, respectively, as observed for the human A_1_AR. The orthosteric binding pocket where the tricyclic purinedione binds is largely identical among all subtypes of human ARs. The 3,4-dichlorobenzyl substitution at N8 also occupies a similar binding pocket in the human A_2A_ AR as found in docking studies of the human A_1_ AR. However, the lack of electrostatic interactions with the chloro substituents at the benzyl moiety may reduce the binding affinity at the human A_2A_ AR in comparison to the human A_1_ AR, since the glutamic acid (Glu170 in the human A_1_ AR) is replaced with a non-polar leucine (Leu167) in the human A_2A_ AR. The selectivity of compound **16a** vs. the two other human AR subtypes, A_2B_ and A_3_, may be explained by different residues, lysine and glutamate, respectively, which are present in the binding pocket in comparison to Ser267 (human A_1_ AR) or Leu267 (human A_2A_ AR). The different amino acid residues would require different substitution patterns on the tricyclic ring system in order to result in binding affinity for the human A_2B_ and A_3_ ARs.

**Figure 6:**
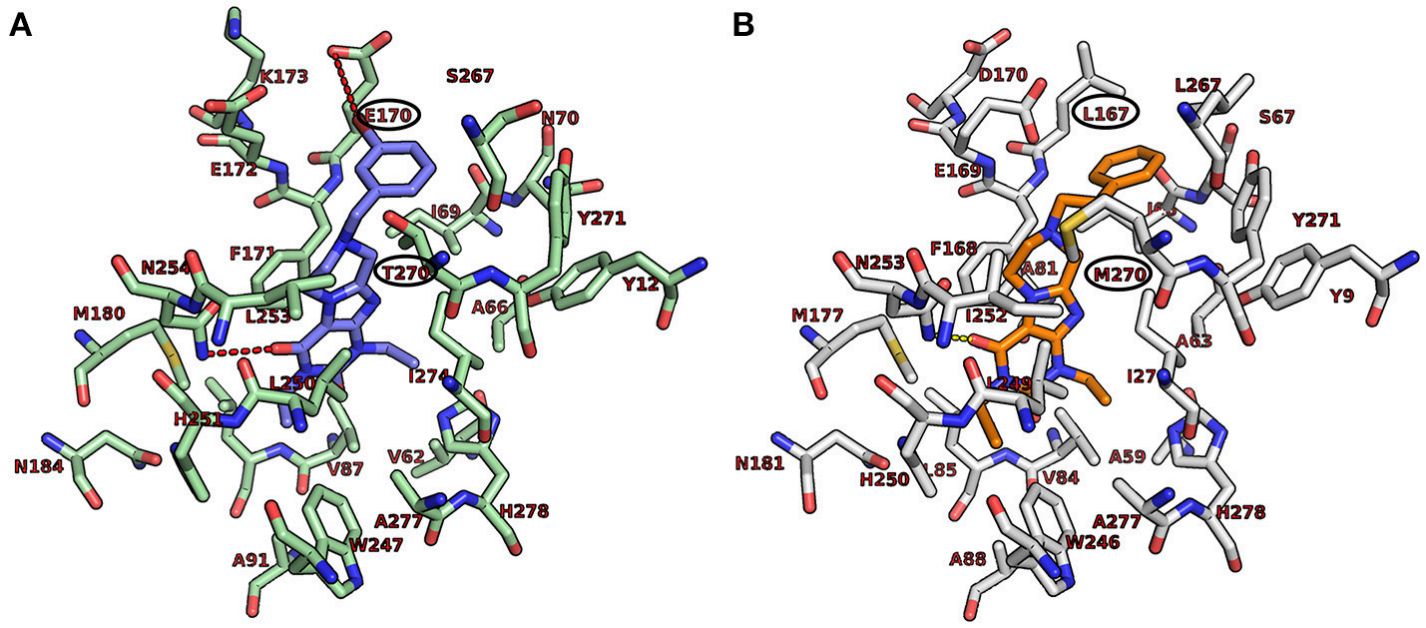
Putative binding pose of **15d** (A_1_-selective) and **20h** (A_2A_-selective). **(A)** Docked pose of **15d** (marine blue) with the important amino acids in the binding pocket of the human A_1_ AR. **(B)** Docked pose of **20h** (orange) with the important amino acids in the binding pocket of the human A_2A_ AR. The interactions are indicated by red dotted lines and the important amino acids responsible for selectivity are encircled in black.

We additionally docked the A_1_-selective compound **15d** (Figure 6A), which contains a *m*-bromobenzyl residue. Its selectivity for the A_1_ vs. the A_2A_ AR can be explained by strong electrostatic interactions between bromine and Glu170. This may be the reason for the increased affinity of the bromo-substituted benzyl derivative in comparison to the fluoro- (**15b**) or the chloro- (**15c**) substituted analogs. The A_2A_-selectivity of compound **20h** (Figure 6B), a phenethyl derivative, is likely due to strong hydrophobic interaction with Met270, which controls the positioning of the compound toward the binding pocket. The obtained orientation of **20h** may be further stabilized by hydrophobic interactions with the residues Leu167 and Leu267. The proposed hypothesis that the hydrophobic residue Met270 likely plays a role is supported by the results at the rat A_1_ AR, in which that methionine is replaced by isoleucine (Ile270) showing better affinity in comparison to threonine (Thr270) present in the human A_1_ AR (see Sequence Alignment in Figure S1, Supplementary Material).

#### Species differences at adenosine receptors

**Figure 7:**
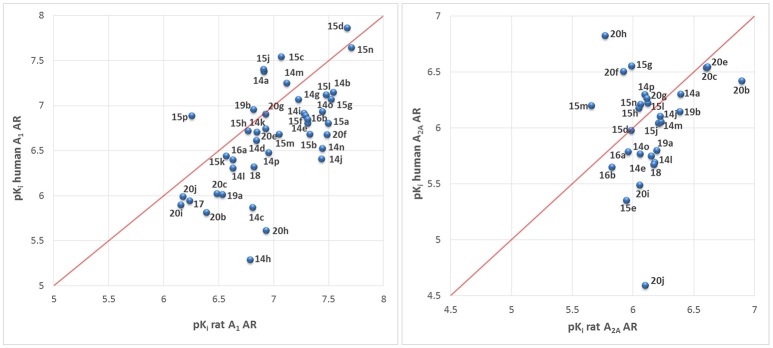
Correlation of affinities at human vs. rat A_1_ and A_2A_ ARs.

The majority of compounds was investigated at both, rat as well as human A_1_ and A_2A_ ARs. This was done because previous studies had revealed major species differences for some classes of AR antagonists between human and rodent receptors (Maemoto et al., 1997; Burbiel et al., 2016; Szymanska et al., 2016). Rats or mice are typically used for preclinical studies. Therefore, it is important to determine the affinity of tool compounds and preclinical drug candidates in rodent species. The correlation of pK_i_ values obtained at rat vs. human A_1_ and A_2A_ ARs is depicted in Figure 7. Many of the compounds were somewhat more potent at rat than at human receptors, although for some compounds the opposite was true. The correlation was in general quite good (less than 3–5-fold difference in K_i_ values), although some outliers were observed (see Figure 7) confirming subtle differences in the binding sites.

#### Structure-activity relationships at monoamine oxidases

The inhibitory activities of the tetrahydropyrazino[2,1-*f* ]purinediones **13**–**20** at human MAO-A and MAO-B are listed in Table 6. All compounds, tested at a concentration of 10 μM, were found to be inactive at MAO-A. In case of MAO-B, compound **13g** bearing a 3,4-dichlorophenethyl moiety at the N8-position was the only example within the series of 1-methyltetrahydropyrazino[2,1-*f* ]purinediones **13** displaying an IC_50_ value in the submicromolar range. Alteration of the substitution pattern on the phenyl ring from 3,4-dichloro to 2,4-dichloro resulted in a 3-fold reduction in MAO-B inhibitory potency. The length of the linker between N8 and the di-chlorophenyl ring was not that important for the inhibitory activity. Shortening of the linker by one methylene group resulted only in a negligible decrease of inhibitory activity (compare **13h** vs. **13g**).

**Table 6:** MAO-A and MAO-B inhibition of tetrahydropyrazino[2,1-*f* ]purinediones **13–20** and standard inhibitors.

	**IC**_**50**_ ± **SEM (nM)^a^**	
**Compd**.	**Human MAO-A**	**Human MAO-B**
**STANDARD COMPOUNDS**		
Selegiline	nd^b^	6.13 ± 0.85
Safinamide	nd	7.67 ± 1.81
Lazabemide	nd	17.6 ± 4.2
Istradefylline (**1**)	>10,000 (18%)^c^	>10,000 (20%)^c^
Caffeine (**2**)	>500,000 (33%)^c^	>500,000 (16%)^c^
CSC (**3**)	>10,000 (23%)^c^	18.1 ± 3.3
**1-METHYLTETRAHYDROPYRAZINO[2,1-*****f*****]PURINEDIONES 13**		
**13a**	>10,000 (13%)^c^	4,530 ± 150
**13b**	nd	>10,000 (14%)^c^
**13c**	nd	ca. 10,000 (54%)^c^
**13e**	nd	ca. 10,000 (47%)^c^
**13f**	>10,000 (13%)^c^	2,880 ± 330
**13g**	>10,000 (12%)^c^	864 ± 64
**13h**	>10,000 (12%)^c^	1,090 ± 70
**3-ETHYL-1-METHYLTETRAHYDROPYRAZINO[2,1-*****f*****]PURINEDIONES 14**		
**14a**	nd	>10,000 (37%)^c^
**14b**	nd	>10,000 (34%)^c^
**14c**	>10,000 (2%)^c^	1,630 ± 120
**14d**	>10,000 (5%)^c^	≥10,000 (49%)^c^
**14e**	>10,000 (12%)^c^	<10,000 (64%)^c^
**14f**	nd	>10,000 (32%)^c^
**14g**	nd	≥10,000 (48%)^c^
**14h**	nd	>10,000 (24%)^c^
**14i**	>10,000 (20%)^c^	≤10,000 (54%)^c^
**14j**	>10,000 (23%)^c^	3,980 ± 750
**14k**	nd	>10,000 (43%)^c^
**14l**	>10,000 (8%)^c^	<10,000 (65%)^c^
**14m**	>10,000 (9%)^c^	1,430 ± 60
**14n**	>10,000 (19%)^c^	3,740 ± 350
**14o**	>10,000 (15%)^c^	3,070 ± 180
**14p**	nd	>10,000 (23%)^c^
**14q**	>10,000 (8%)^c^	<10,000 (56%)^c^
**1,3-DIETHYLTETRAHYDROPYRAZINO[2,1-*****f*****]PURINEDIONES 15**		
**15a**	nd	>10,000 (22%)^c^
**15b**	nd	≥10,000 (49%)^c^
**15c**	nd	≥10,000 (47%)^c^
**15d (PSB-18339)**	nd	≥10,000 (49%)^c^
**15e**	>10,000 (25%)^c^	6,510 ± 270
**15f**	nd	>10,000 (41%)^c^
**15g**	nd	>10,000 (10%)^c^
**15h**	nd	>10,000 (32%)^c^
**15i**	>10,000 (8%)^c^	3,070 ± 200
**15j**	>10,000 (7%)^c^	<10,000 (58%)^c^
**15k**	>10,000 (−10%)^c^	<10,000 (63%)^c^
**15l**	>10,000 (8%)^c^	1,310 ± 160
**15m**	>10,000 (1%)^c^	<10,000 (66%)^c^
**15n**	>10,000 (−4%)^c^	≤10,000 (54%)^c^
**15o**	nd	>10,000 (5%)^c^
**15p**	>10,000 (6%)^b^	<10,000 (67%)^c^
**15q**	nd	>10,000 (27%)^c^
**15r**	>10,000 (7%)^c^	524 ± 26
**1-ETHYL-3-METHYLTETRAHYDROPYRAZINO[2,1-*****f*****]PURINEDIONES 16**		
**16a (PSB-18405)**	>10,000 (6%)^c^	106 ± 10
**16b**	>10,000 (8%)^c^	136 ± 5
**1-CYCLOPROPYL-3-METHYLTETRAHYDROPYRAZINO[2,1-*****f*****]PURINEDIONE 17**		
**17**	>10,000 (7%)^c^	3,690 ± 250
**1-METHYL-3-PROPYLTETRAHYDROPYRAZINO[2,1-*****f*****]PURINEDIONE 18**		
**18**	>10,000 (9%)^c^	2,910 ± 110
**1-METHYL-3-PROPARGYLTETRAHYDROPYRAZINO[2,1-*****f*****]PURINEDIONES 19**		
**19a**	>10,000 (12%)^c^	679 ± 17
**19b**	>10,000 (5%)^c^	<10,000 (59%)^c^
**1-ETHYL-3-PROPARGYLTETRAHYDROPYRAZINO[2,1-*f*]PURINEDIONES 20**		
**20a**	nd	>10,000 (29%)^c^
**20b**	nd	>10,000 (35%)^c^
**20c**	>10,000 (6%)^c^	≤10,000 (51%)^c^
**20d**	nd	>10,000 (13%)^c^
**20e (PSB-1869)**	nd	>10,000 (20%)^c^
**20f**	nd	>10,000 (27%)^c^
**20g**	nd	>10,000 (41%)^c^
**20h**	nd	>10,000 (27%)^c^
**20i**	nd	>10,000 (26%)^c^
**20j**	nd	>10,000 (30%)^c^
**20k**	nd	>10,000 (25%)^c^
**20l**	nd	>10,000 (3%)^c^

a *n = 3*.

b *nd, not determined*.

c *% inhibition at indicated concentration*.

Moderately to weakly active MAO-B inhibitors could also be identified in the series of 3-ethyl-1-methyltetrahydropyrazino[2,1-*f* ]purinediones **14**. In the group of compounds having a mono-substituted (halogen or trifluoromethyl) benzyl ring at the N8-position (**14a**-**i**), 4-bromo-derivative **14c** displayed the highest MAO-B inhibitory potency (IC_50_ = 1,630 nM). Compounds **14e** and **14i** having a CF_3_ or Cl at position 3 of the aromatic ring, respectively, also showed an inhibition of greater than 50% at a test concentration of 10 μM. In case of the compounds bearing two substituents on the phenyl ring, a 2,5-disubstitution pattern was shown to be favorable for MAO-B inhibition (compounds **14j**, **14m**, **14o**).

Within the series of 1,3-diethyl-substituted tetrahydropyrazino[2,1-*f* ]purinediones **15**, compounds having a di-substituted phenyl ring (**15i**-**q**) showed a higher MAO-B inhibitory potency as compared to the mono-substituted derivatives of this series (**15a**-**h**). In general, a 3,5- and a 3,4-disubstitution pattern of the phenyl ring was beneficial for MAO-B inhibition. The most potent MAO-B inhibitor of this series was derivative **15r** (IC_50_ = 524 nM) bearing an 3,4,5-trifluorobenzyl moiety at the N8-position. This is in good agreement with results observed within the reported 1,3-dimethyltetrahydropyrazino[2,1-*f* ]purine-2,4-dione series (Brunschweiger et al., 2014).

Comparison of all tetrahydropyrazino[2,1-*f* ]purinediones bearing a 3,4-dichlorobenzyl moiety at the N8-position (compounds **13h**, **16a**, **17**, **18**, and **19a**) revealed that the N1-ethyl and N3-methyl substitution pattern was the best for MAO-B inhibition. Dual A_1_/A_2A_ AR antagonist **16a** was the most potent MAO-B inhibitor of the present series showing an IC_50_ value in the nanomolar range (IC_50_ = 106 nM). Dual A_1_/A_2A_ AR antagonist **16b** having a 3,5-dichloro substitution pattern on the benzene ring inhibited MAO-B (IC_50_ = 136 nM) with almost equal potency as its 3,4-dichloro isomer **16a**.

The only compound of the 3-ethyl-1-methyltetrahydropyrazino[2,1-*f* ]purinediones series **20** displaying inhibition of more than 50% at a high test concentration of 10 μM was **20c** bearing a 3-methoxyphenyl moiety at the N8-position, whereas all other derivatives of this series were inactive.

#### Docking studies at monoamine oxidase B

**Figure 8:**
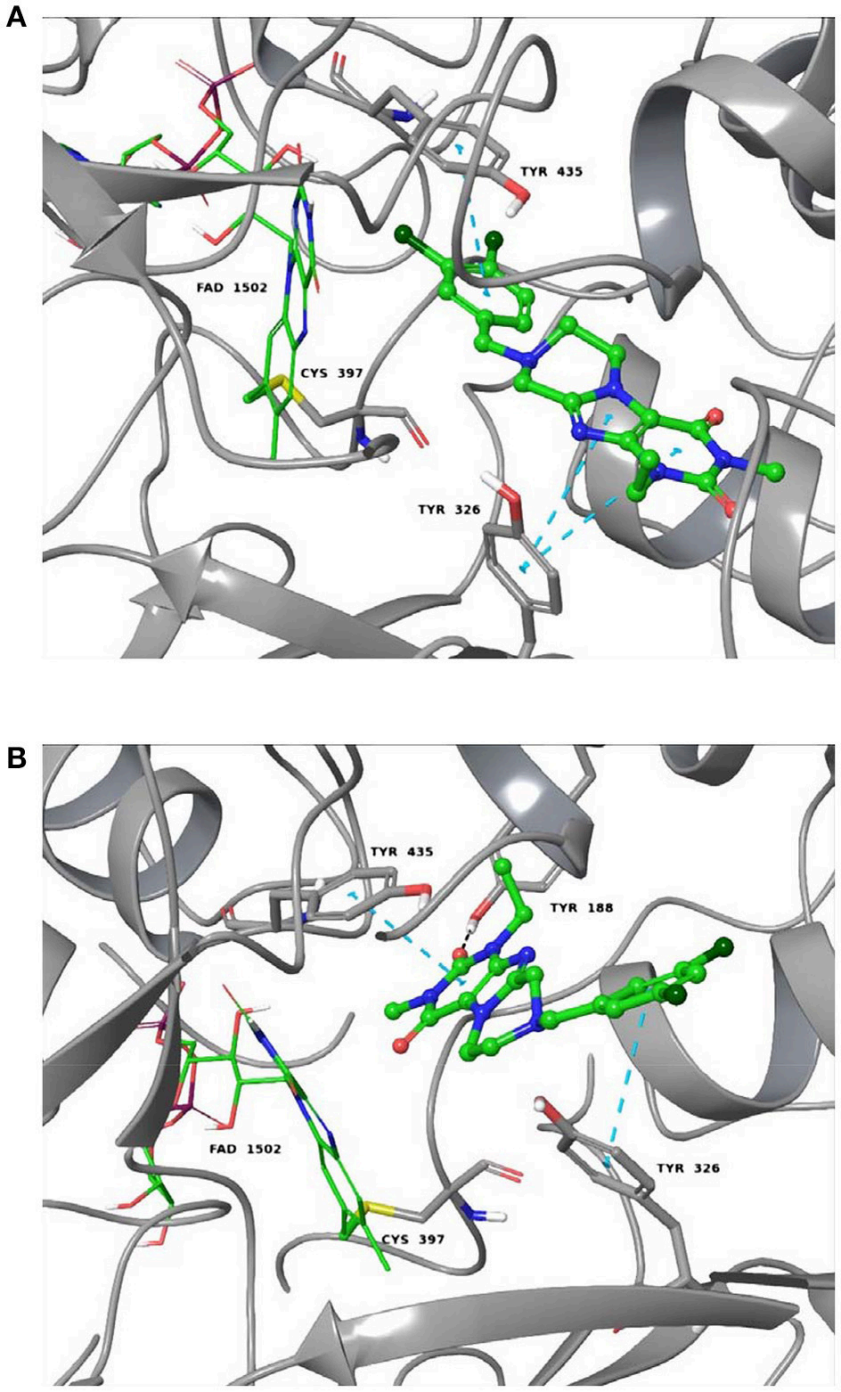
Molecular interactions **(A)** between inhibitor **16a** and MAO-B, and **(B)** between **16b** and MAO-B. The protein is shown as gray cartoon while the ligands are shown as green carbon sticks; H-bonds and π-π interactions are depicted as dashed black and cyan lines respectively. Amino acid residues involved in the interactions are shown as gray sticks. The covalently bound cofactor flavine adenine dinucleotide (FAD) is depicted on the left hand side as green sticks. Nitrogen atoms are in blue, oxygen atoms in red, hydrogen atoms in white, phosphorus atoms in purple.

In Figure 8, the main interactions between MAO-B and inhibitors **16a** (**A**) and **16b** (**B**), the two active compounds, are depicted. Stacking interactions appear to stabilize the tricyclic inhibitors **16a** and **16b** within the MAO-B binding site. In case of **20l**, which was randomly selected from the group of inactive compounds, this kind of contacts are not detected, which may be the reason for its different biological profile. It is worth noting that Tyr326 in the active site plays an essential role in binding: it is likely involved in interactions with the two active inhibitors **16a** and **16b**, as with the standard inhibitor safinamide (Binda et al., 2007). Moreover, as we recently observed in case of linear ligands (Carradori et al., 2016; Meleddu et al., 2017), opposite head-tail orientations can easily occur according to docking results due to comparable energy levels. The presence of Cl at position 5 on the benzyl moiety of **16b** induces a modest steric hindrance with Tyr188, sufficient to force its inverted orientation in the best pose with respect to **16a**.

A comparative analysis performed by the induced-fit docking G-score estimation revealed that compound **16b** can establish a better molecular recognition within the MAO-B isoform with respect to MAO-A. The G-score gap is in the order of about 6.7 kcal/mol and likely due to the different binding pocket volumes between those isoforms (Alcaro et al., 2010). The π-π interaction of **16b** with Tyr326 in MAO-B (Figure 8B) cannot be established in the isoform A, where this residue is replaced with and Ile residue.

#### Water solubility

1,3,8-Substituted tetrahydropyrazino[2,1-*f* ]purine-2,4(1*H*,3*H*)-diones have previously been shown to possess excellent water-solubility at pH 1 due to protonation of the nitrogen atom N8 (Brunschweiger et al., 2014). This is expected to facilitate dissolution in the stomach. Depending on the substitution pattern they may also be well soluble at higher pH values (Brunschweiger et al., 2014). In the present study we measured thermodynamic solubility of only very few compounds exemplarily (**14a**-**e**, **14g, 15f**,**g**) and confirmed their high solubility at pH 1 (see Table S1 in Supplementary Material). Most of these compounds were still soluble at pH 7.4. The best soluble derivative was the 3-ethyl-1-methyl-8-*m*-fluorobenzyl derivative **14g** with solubilities of >1.5 g/L (4.2 mM) at pH 1, 50 mg/L (0.14 mM) at pH 4, and 40 mg/L (0.084 mM) at pH 7.4.

## Conclusions

A large library of novel 1,3,8-substituted tetrahydropyrazino[2,1-*f* ]purinediones was synthesized. For the first time we systematically and extensively studied the exchange of methyl groups in the 1- and 3-position of the theophylline-/caffeine-derived tricyclic scaffold for a variety of alkyl residues including cyclic and unsaturated ones. Series of compounds with different 1- and 3-substituent were also obtained. The compounds were tested for antagonistic potency at all four ARs as well as for inhibitory potency at both MAO enzymes with the goal to improve potency at A_1_ and A_2A_ ARs and MAO-B, which are (potential) targets for the treatment of neurodegenerative diseases, in particular for PD. The A_1_ AR affinity was dramatically improved by 3-ethyl-1-methyl (e.g., **14a**), 1,3-diethyl (**15d**) and 1-ethyl-3-propargyl (**20e**) substitution. Good A_2A_ affinity was obtained for 3-ethyl-1-propargyl derivatives **20**, e.g., **20h** and **20e**. Compounds with a balanced A_1_/A_2A_ inhibitory potency were also obtained in this group (e.g., **20e**). Significantly increased MAO-B inhibitory activity while keeping selectivity vs. the isoenzyme MAO-A, which is important to avoid side-effects, was obtained by 1-ethyl-3-methyl substitution of the pyrazinopurinedione structure (**16a**, **16b**). Molecular docking studies based on recently published X-ray structures of A_1_ and A_2A_ ARs, and of MAO-B supported the results obtained by SAR analysis. Besides modulating affinities, replacement of the methyl groups in the 1- and 3-position of pyrazinopurinediones may also be useful for fine-tuning metabolic stability of the compounds since the methyl groups of caffeine and theophylline are known to be subject to oxidative demethylation (Fredholm et al., 1999). The present study provides new insights into the SARs of tricyclic xanthine derivatives, which are suitable scaffolds for multi-target drug development. As a next important step, such multi-target drugs will have to be tested in *in vivo* models in comparison to drugs selective for a single target to prove their potential superiority.

## Author contributions

CM and AB designed the study inspired by KK-K, and CM supervised the experiments; AB, SU, and JHo performed the syntheses; BL, SH and PKü performed the biological evaluation of the compounds; PKo and CM analyzed the data and wrote the manuscript. VN, AM, SA, MW, and CM supervised and/or performed the molecular modeling studies. All authors read and contributed to the manuscript.

### Conflict of interest statement

The authors declare that the research was conducted in the absence of any commercial or financial relationships that could be construed as a potential conflict of interest.
