# Supplementary material for: Probing Substituents in the 1- and 3-Position: Tetrahydropyrazino-Annelated Water-Soluble Xanthine Derivatives as Multi-Target Drugs With Potent Adenosine Receptor Antagonistic Activity
Source: Front Chem. 2018 Jun 26;6:206. doi: 10.3389/fchem.2018.00206 (PMC6028563; doi:10.3389/fchem.2018.00206)
Supplement: Supplementary file 1 [file Data_Sheet_1.PDF]

Supplementary Material

Probing substituents in the 1- and 3-position: Tetrahydropyrazino-annelated water-soluble xanthine derivatives as multi-target drugs with potent adenosine receptor antagonistic activity

Pierre Koch,[1,#] Andreas Brunschweiger,[1] Vigneshwaran Namasivayam,[1] Stefan Ullrich,[1] Annalisa Maruca,[3] Beatrice Lazzaretto,[1] Petra Küppers,[1] Sonja Hinz,[1] Jörg Hockemeyer,[1] Michael Wiese,[2] Jag Heer,[4] Stefano Alcaro,[3] Katarzyna Kiec-Kononowicz,[5] and Christa E. Müller[1,*]

[1]PharmaCenter Bonn, Pharmaceutical Institute, Pharmaceutical Chemistry I, University of Bonn, An der Immenburg 4, 53121 Bonn, Germany

[2]Pharmaceutical Institute, Pharmaceutical Chemistry II, University of Bonn, An der Immenburg 4, 53121 Bonn, Germany

[3]Dipartimento di Scienze della Salute, Università "Magna Græcia" di Catanzaro, Campus Universitario "S. Venuta", Viale Europa, Loc. Germaneto, 88100 Catanzaro, Italy

[4]Jag Heer, UCB Celltech, UCB Pharma S.A., Slough, Berkshire, United Kingdom

[5]Department of Technology and Biotechnology of Drugs, Faculty of Pharmacy, Jagiellonian University Medical College, 30688 Kraków, Poland

*** Correspondence:** Christa E. Müller: christa.mueller@uni-bonn.de

Table of Contents

Synthesis of intermediates **22**, **23**, **11**, **12** and **29**. S2

Carbon NMR of final compounds. S6

Figure S1. Sequence alignment of human and rat A_1 and A_{2A} adenosine receptor subtypes. S16

Table S1. Solubility of selected compounds at three different pH values (in mg/mL). S17

Synthesis of intermediates 22, 23, 11, 12 and **29.**

General procedure for the preparation of 8-hydroxymethylpurine-2,4-diones (22a-e) (General procedure E)

5,6-Diaminouracil **21a-e** (5 g) and glycolic acid (1.2 equiv.) were stirred without solvent at 100 °C for 1 h. After 2 min, the mixture usually liquefied to turn solid again after a few minutes. After 1 h H_2O (50 mL) was added to the solid. The pH of the solution was set to 12-13 by addition of NaOH pellets. To effect ring closure the alkaline solution was stirred for 4 h at 100 °C. Then, it was cooled to 0 °C and acidified by addition of conc. aq. HCl. The 8-hydroxymethylxanthine precipitated slowly overnight at 4 °C in the fridge. It was filtered off and washed with H_2O (3 × 20 mL) and MeOH (2 ×10 mL).

3-Ethyl-8-(hydroxymethyl)-1*H*-purine-2,6(3*H*,7*H*)-dione (22a)

General procedure E. Yield: 85 %; mp: 285 °C; ^{1}H-NMR (MeOD) δ 4.73 (s, 2H, 8-CH_2), 4.13 (q, ^3J= 6.90 Hz, 2H, N3-CH_2), 1.34 (t, ^3J= 7.25 Hz, 3H, N3-CH_2-$\underline{CH_3}$). ^{13}C-NMR (MeOD) δ 156.6 (C-9a), 155.7 (C-4), 153.7 (C-2), 150.0 (C-10a), 108.6 (C-4a), 58.8 (8-CH_2), 39.3 (N3-CH_2), 13.9 (N3-CH_2-$\underline{CH_3}$). ESI-MS: negative mode 209.2 $[M\text{-}H]^-$, positive mode 211.0 $[M+H]^+$.

3-Cyclopropyl-8-(hydroxymethyl)-1*H*-purine-2,6(3*H*,7*H*)-dione (22b)

General procedure E. Yield: 82 %; mp: 286 °C; ^{1}H-NMR (MeOD) δ 4.75 (s, 2H, 8-CH_2), 2.97-2.93 (m, 2H, H-1, cyclopropyl), 1.20-1.16 (m, 2H, H-2 and H-3, cyclopropyl), 1.05-1.01 (m, 2H, H-2 and H-3, cyclopropyl). ^{13}C-NMR (MeOD) δ 156.6 (C-9a), 155.7 (C-4), 153.7 (C-2), 150.0 (C-10a), 108.6 (C-4a), 58.8 (8-CH_2), 26.1 (C-1, cyclopropyl), 9.1 (C-2 and C-3, cyclopropyl). ESI-MS: negative mode 209.2 $[M\text{-}H]^-$, positive mode 211.0 $[M+H]^+$.

8-(Hydroxymethyl)-3-methyl-1*H*-purine-2,6(3*H*,7*H*)-dione (ABS 198a) (22c)

General procedure E. Yield: 88 %; mp: 282 °C; ^{1}H-NMR (MeOD) δ 4.73 (s, 2H, 8-CH_2), 3.53 (s, 3H, N3-CH_3). ^{13}C-NMR (MeOD) δ 156.6 (C-9a), 155.7 (C-4), 153.7 (C-2), 150.0 (C-10a), 108.6 (C-4a), 58.9 (8-CH_2), 29.8 (N3-CH_3). ESI-MS: negative mode 195.2 $[M\text{-}H]^-$, positive mode 197.2 $[M+H]^+$.

1-Ethyl-8-(hydroxymethyl)-3-methyl-1*H*-purine-2,6(3*H*,7*H*)-dione (22d)

General procedure E. Yield: 80 %; mp: 266 °C; ^{1}H-NMR (MeOD) δ 4.73 (s, 2H, 8-CH_2), 4.08 (q, ^3J= 6.90 Hz, 2H, N1-CH_2), 3.58 (s, 3H, N3-CH_3), 1.25 (t, ^3J= 7.25 Hz, 3H, N1-CH_2-$\underline{CH_3}$). ^{13}C-NMR (MeOD) δ 156.6 (C-9a), 155.7 (C-4), 153.7 (C-2), 150.0 (C-10a), 108.6 (C-4a), 58.8 (8-CH_2), 37.8 (N1-CH_2), 30.6 (N3-CH_3), 13.8 (N1-CH_2-$\underline{CH_3}$). ESI-MS: negative mode 223.2 $[M\text{-}H]^-$, positive mode 225.2 $[M+H]^+$.

1,3-Diethyl-8-(hydroxymethyl)-1*H*-purine-2,6(3*H*,7*H*)-dione (22e)

General procedure E. Yield: 72 %; mp: 235 °C; ^{1}H-NMR (MeOD) δ 4.73 (s, 2H, 8-CH_2), 4.13 (q, ^3J= 6.90 Hz, 2H, N3-CH_2), 4.08 (q, ^3J= 6.90 Hz, 2H, N1-CH_2), 1.32 (t, ^3J= 7.25 Hz, 3H, N3-CH_2-$\underline{CH_3}$), 1.25 (t, ^3J= 7.25 Hz, 3H, N1-CH_2-$\underline{CH_3}$). ^{13}C-NMR (MeOD) δ 156.6 (C-9a), 155.7 (C-4), 153.7 (C-2), 150.0 (C-10a), 108.6 (C-4a), 58.8 (8-CH_2), 38.5 (N3-CH_2), 36.3 (N1-CH_2), 13.8 (N3-CH_2-$\underline{CH_3}$), 13.6 (N1-CH_2-$\underline{CH_3}$). ESI-MS: negative mode 237.2 $[M\text{-}H]^-$, positive mode 239.2 $[M+H]^+$.

General procedure for the preparation of 7-(2-bromoethyl)-8-hydroxymethylpurine-2,4-diones 23a-e (General procedure F)

8-Hydroxymethylpurine-2,4-dione **22a-e** (1.0 g) was dissolved in a mixture of DMF (10 mL), DIPEA (2 mL) and 1,2-dibromoethane (1 mL). The solution was stirred overnight at 70 °C. Then, the volatiles were removed by rotary evaporation and the product was purified by silica gel chromatography using a gradient of CH_2Cl_2 to CH_2Cl_2/MeOH 40:1 as eluent.

7-(2-Bromoethyl)-3-ethyl-8-(hydroxymethyl)-1*H*-purine-2,6(3*H*,7*H*)-dione (23a)

General procedure F. Yield: 54 %; mp: 250 °C; ^{1}H-NMR (MeOD) δ 4.81 (t, ^3J= 6.60 Hz, 2H, N7-CH_2), 4.74 (s, 2H, 8-CH_2), 4.08 (q, ^3J= 6.90 Hz, 2H, N3-CH_2), 3.88 (t, ^3J= 6.65 Hz, 2H, N7-CH_2-$\underline{CH_2}$), 1.32 (t, ^3J= 7.25 Hz, 3H, N3-CH_2-$\underline{CH_3}$). ^{13}C-NMR (MeOD) δ 156.6 (C-9a), 155.7 (C-4), 153.7 (C-2), 150.0 (C-10a), 108.6 (C-4a), 58.8 (8-CH_2), 50.7 (N7-CH_2), 37.3 (N3-CH_2), 13.8 (N3-CH_2-$\underline{CH_3}$). ESI-MS: negative mode 315.2 and 317.2 $[M-H]^-$, positive mode 317.0 and 319.0 $[M+H]^+$.

7-(2-Bromoethyl)-3-cyclopropyl-8-(hydroxymethyl)-1*H*-purine-2,6(3*H*,7*H*)-dione (23b)

General procedure F. Yield: 63 %; mp: 240 °C; ^{1}H-NMR (MeOD) δ 4.81 (t, ^3J= 6.60 Hz, 2H, N7-CH_2), 4.74 (s, 2H, 8-CH_2), 3.88 (t, ^3J= 6.65 Hz, 2H, N7-CH_2-$\underline{CH_2}$), 2.97-2.93 (m, 2H, H-1, cyclopropyl), 1.20-1.16 (m, 2H, H-2 and H-3, cyclopropyl), 1.05-1.01 (m, 2H, H-2 and H-3, cyclopropyl). ^{13}C-NMR (MeOD) δ 156.6 (C-9a), 155.7 (C-4), 153.7 (C-2), 150.0 (C-10a), 108.6 (C-4a), 58.8 (8-CH_2), 50.7 (N7-CH_2), 26.1 (C-1, cyclopropyl), 9.1 (C-2 and C-3, cyclopropyl). ESI-MS: negative mode 326.2 and 328.2 $[M-H]^-$, positive mode 328.0 and 330.0 $[M+H]^+$.

7-(2-Bromoethyl)-8-(hydroxymethyl)-3-methyl-1*H*-purine-2,6(3*H*,7*H*)-dione (23c)

General procedure F. Yield: 62 %; mp: 256 °C; ^{1}H-NMR (MeOD) δ 4.81 (t, ^3J= 6.60 Hz, 2H, N7-CH_2), 4.74 (s, 2H, 8-CH_2), 3.88 (t, ^3J= 6.65 Hz, 2H, N7-CH_2-$\underline{CH_2}$), 3.54 (s, 3H, N3-CH_3). ^{13}C-NMR (MeOD) δ 156.6 (C-9a), 155.7 (C-4), 153.7 (C-2), 150.0 (C-10a), 108.6 (C-4a), 58.8 (8-CH_2), 50.7 (N7-CH_2), 31.7 (N7-CH_2-$\underline{CH_2}$), 30.0 (N3-CH_3). ESI-MS: negative mode 301.2 and 303.2 $[M-H]^-$, positive mode 303.0 and 305.0 $[M+H]^+$.

7-(2-Bromoethyl)-1-ethyl-8-(hydroxymethyl)-3-methyl-1*H*-purine-2,6(3*H*,7*H*)-dione (23d)

General procedure F. Yield: 67 %; mp: 256 °C; ^{1}H-NMR (MeOD) δ 4.81 (t, ^3J= 6.60 Hz, 2H, N7-CH_2), 4.74 (s, 2H, 8-CH_2), 4.08 (q, ^3J= 6.90 Hz, 2H, N1-CH_2), 3.88 (t, ^3J= 6.65 Hz, 2H, N7-CH_2-$\underline{CH_2}$), 3.54 (s, 3H, N3-CH_3), 1.25 (t, ^3J= 7.25 Hz, 3H, N1-CH_2-$\underline{CH_3}$). ^{13}C-NMR (MeOD) δ 156.6 (C-9a), 155.7 (C-4), 153.7 (C-2), 150.0 (C-10a), 108.6 (C-4a), 58.8 (8-CH_2), 50.7 (N7-CH_2), 37.8 (N1-CH_2), 31.7 (N7-CH_2-$\underline{CH_2}$), 30.0 (N3-CH_3), 13.8 (N1-CH_2-$\underline{CH_3}$). ESI-MS: negative mode 328.2 and 330.2 $[M-H]^-$, positive mode 330.0 and 332.0 $[M+H]^+$.

7-(2-Bromoethyl)-1,3-diethyl-8-(hydroxymethyl)-1*H*-purine-2,6(3*H*,7*H*)-dione (23e)

General procedure F. Yield: 77 %; mp: 213 °C; ^{1}H-NMR (MeOD) δ 4.81 (t, ^3J= 6.60 Hz, 2H, N7-CH_2), 4.74 (s, 2H, 8-CH_2), 4.13 (q, ^3J= 6.90 Hz, 2H, N3-CH_2), 4.08 (q, ^3J= 6.90 Hz, 2H, N1-CH_2), 3.88 (t, ^3J= 6.65 Hz, 2H, N7-CH_2-$\underline{CH_2}$), 1.32 (t, ^3J= 7.25 Hz, 3H, N3-CH_2-$\underline{CH_3}$), 1.25 (t, ^3J= 7.25 Hz, 3H, N1-CH_2-$\underline{CH_3}$). ^{13}C-NMR (MeOD) δ 156.6 (C-9a), 155.7 (C-4), 153.7 (C-2), 150.0 (C-10a), 108.6 (C-4a), 58.8 (8-CH_2), 50.7 (N7-CH_2), 38.5 (N3-CH_2), 36.3 (N1-CH_2), 13.4 (N1-CH_2-$\underline{CH_3}$), 13.3 (N3-CH_2-$\underline{CH_3}$). ESI-MS: negative mode 342.2 and 344.2 $[M-H]^-$, positive mode 344.0 and 346.0 $[M+H]^+$.

General procedure for the preparation of 8-substituted 6,7,8,9-tetrahydropyrazino[2,1-*f*]purine-2,4(1*H*,3*H*)-diones (General procedure A)

7-(2-Bromoethyl)-8-hydroxymethylpurine-2,4-dione (400 mg) was dissolved in dry CH_2Cl_2 (50 mL). The solution was cooled to 0 °C and PBr_3 (0.4 mL) was added dropwise. The solution was allowed to warm to rt and stirred for 1 h. Then it was cooled to 0 °C again. To hydrolyze the excess of PBr_3 saturated aq. $NaHCO_3$-solution (5 mL) was added and the pH was set to 7-8 by addition of $NaHCO_3$. Then, the lower layer was separated in a separating funnel and the aqueous layer was extracted with CH_2Cl_2 (2 × 50 mL). The organic extracts were combined, dried over Na_2SO_4 and the solvent was removed by rotary evaporation. The residue was dissolved in a mixture of dimethoxyethane (10 mL) and DIPEA (0.5 mL). To effect ring closing reaction, an appropriate amine was added and the solution was stirred overnight at rt. The volatiles were removed by rotary evaporation and the product precipitated upon addition of H_2O (20 mL). For purification, the compound was either filtered off and washed with H_2O (3 × 5 mL) and diethylether (3 × 10 mL) or subjected to flash-chromatography (silica gel, CH_2Cl_2:MeOH 1:0 to 40:1).

8-(3,4-Dichlorobenzyl)-1-ethyl-6,7,8,9-tetrahydropyrazino[2,1-*f*]purine-2,4(1*H*,3*H*)-dione (11a)

General procedure A. Yield: 68 %; mp: 226 °C; ^{1}H-NMR ($CDCl_3$) δ 7.44 (d, 1H, ^4J= 1.90 Hz, H-2, phenyl), 7.43 (d, 1H, ^3J= 8.20 Hz, H-5, phenyl), 7.20 (dd, 1H, ^3J= 8.20 Hz, ^4J= 2.40 Hz, H-6, phenyl), 4.34 (t, 2H, ^3J= 5.35 Hz, 2 x H-6), 4.00 (q, 2H, ^3J= 7.25 Hz, N1-CH_2), 3.76 (s, 2H, N8-CH_2), 3.74 (s, 2H, 2 x H-9), 2.96 (t, 2H, ^3J= 5.35 Hz, 2 x H-7), 1.32 (t, 3H, ^3J= 7.25 Hz, N1-CH_2-$\underline{CH_3}$). ^{13}C-NMR ($CDCl_3$) δ 154.5 (C-9a), 150.6 (C-4), 149.5 (C-2), 148.2 (C-10a), 136.9 (C-1, phenyl), 132.8 (C-3, phenyl), 131.9 (C-4, phenyl), 130.6 and 130.4 (C-2 and C-5, phenyl), 127.7 (C-6, phenyl), 106.9 (C-4a), 60.7 (N8-CH_2), 51.2 (C-7), 48.8 (C-9), 44.2 (C-6), 37.9 (N1-CH_2), 13.4 (N1-CH_2-$\underline{CH_3}$). ESI-MS: positive mode 394.3 $[M+H]^+$. HPLC: 98.2 % (A) and 98.0 % (B).

8-(3,5-Dichlorobenzyl)-1-ethyl-3-methyl-6,7,8,9-tetrahydropyrazino[2,1-*f*]purine-2,4(1*H*,3*H*)-dione (11b)

General procedure A. Yield: 64 %; mp: 235 °C; ^{1}H-NMR ($CDCl_3$) δ 7.28 (d, 1H, ^4J= 1.90 Hz, H-4, phenyl), 7.24 (d, 2H, ^4J= 1.90 Hz, H-2 and H-6, phenyl), 4.36 (t, 2H, ^3J= 5.35 Hz, 2 x H-6), 4.09 (q, 2H, ^3J= 7.25 Hz, N1-CH_2), 3.73 (s, 2H, N8-CH_2), 3.68 (s, 2H, 2 x H-9), 2.95 (t, 2H, ^3J= 5.35 Hz, 2 x H-7), 1.30 (t, 3H, ^3J= 7.25 Hz, N1-CH_2-$\underline{CH_3}$). ^{13}C-NMR ($CDCl_3$) δ 154.2 (C-9a), 150.4 (C-4), 149.7 (C-2), 148.2 (C-10a), 140.2 (C-1, phenyl), 135.3 and 135.0 (C-3 and C-5, phenyl), 128.0 (C-4, phenyl), 127.5 and 127.2 (C-2 and C-6, phenyl), 106.9 (C-4a), 60.9 (N8-CH_2), 51.2 (C-7), 48.9 (C-9), 44.3 (C-6), 38.0 (N1-CH_2), 13.5 (N1-CH_2-$\underline{CH_3}$). ESI-MS: positive mode 394.4 $[M+H]^+$. HPLC: 99.7 % (A) and 99.7 % (B).

8-(3,4-Dichlorobenzyl)-1-cyclopropyl-6,7,8,9-tetrahydropyrazino[2,1-*f*]purine-2,4(1*H*,3*H*)-dione (12)

General procedure A. Yield: 75 %; mp: 247 °C; ^{1}H-NMR ($CDCl_3$) δ 8.03 (br s, 1H, H-3), 7.44 (d, 1H, ^4J= 1.85 Hz, H-2, phenyl), 7.40 (d, 1H, ^3J= 8.20 Hz, H-5, phenyl), 7.16 (dd, 1H, ^3J= 8.20 Hz, ^4J= 2.20 Hz, H-6, phenyl), 4.30 (t, 2H, ^3J= 5.35 Hz, 2 x H-6), 3.73 (s, 2H, N8-CH_2), 3.68 (s, 2H, 2 x H-9), 2.94-2.92 (m, 3H, 2 x H-7 and H-1, cyclopropyl), 1.19-1.12 (m, 2H, H-2 and H-3, cyclopropyl), 1.00-0.96 (2H, m, H-2 and H-3, cyclopropyl). ^{13}C-NMR ($CDCl_3$) δ 154.2 (C-9a), 151.3 (C-4), 150.5 (C-2), 147.9 (C-10a), 137.0 (C-1, phenyl), 132.8 (C-3, phenyl), 131.9 (C-4, phenyl), 130.7 and 130.6 (C-2 and C-5, phenyl), 128.1 (C-6, phenyl), 107.0 (C-4a), 60.7 (N8-CH_2), 51.3 (C-7), 48.8 (C-9), 44.2 (C-6), 25.4

(C-1, cyclopropyl), 8.1 (C-2 and C-3, cyclopropyl). ESI-MS: positive mode 406.0 $[M+H]^+$. ESI-MS: positive mode 406.0 $[M+H]^+$. HPLC: 98.2 % (A) and 97.5 % (B).

Synthesis of 7-(2-Bromoethyl)-8-*N*-boc-aminomethyl-3-methyl-1-propargylxanthine (29).

5,6-Diamino-1-ethyl-3-propargyluracil (**25**) (0.83 g, 4.0 mmol) was dissolved in methanol (10 mL) and added directly to a freshly prepared solution of *N*-Boc-glycine (0.77 g, 4.4 mmol) and EDC (0.84 g, 4.4 mmol) in methanol (10 mL). The reaction mixture was stirred for 1 h at rt, then the solvent was evaporated and the residue crystallized upon addition of water (20 mL). The obtained 5-amido-6-amino-1-methyl-3-propargyluracil **26** (yield: 40 %) was filtered off, dried and used directly in the next step. Compound **26** (0.36 g, 1 mmol) was dissolved in 6 mL of a mixture of aq. 1N-NaOH and dioxane (1:1) and stirred for 10 min at 100°C to effect closure of the xanthine ring. The solution was cooled to rt and concentrated in vacuo to about half of the original volume. Then, H_2O (6 mL) were added, it was cooled to 0°C and carefully acidified to pH 3-4 using aqueous 5N-HCl. The xanthine **27** precipitated from the solution as a sticky mass. The suspension was extracted with CH_2Cl_2 (3 × 20 mL). The extract was dried over Na_2SO_4, and evaporated to dryness. The residue was dissolved in a mixture of 10 mL of DMF and 0.5 mL of DIPEA. To the solution was added 1,2-dibromoethane (940 mg, 5 mmol) and it was stirred at 70 °C for 16 h. Then, the volatiles were removed in vacuo and xanthine **28** was purified by silica gel column chromatography using CH_2Cl_2/MeOH 40:1.

Carbon NMR of final Compounds

8-(2-Bromobenzyl)-1-methyl-6,7,8,9-tetrahydropyrazino[2,1-*f*]purine-2,4(1*H*,3*H*)-dione (13a)

^{13}C-NMR ($CDCl_3$) δ 154.9 (C-9a), 151.7 (C-4), 148.4 (C-2), 147.7 (C-10a), 135.6 (C-1, phenyl), 133.1 (C-3, phenyl), 131.0 (C-6, phenyl), 129.4 (C-4, phenyl), 127.6 (C-5, phenyl), 124.8 (C-2, phenyl), 106.6 (C-4a), 60.8 (N8-CH_2), 51.0 (C-7), 48.8 (C-9), 44.2 (C-6), 29.7 (N1-CH_3).

1-Methyl-8-(3-(trifluoromethyl)benzyl)-6,7,8,9-tetrahydropyrazino[2,1-*f*]purine-2,4(1*H*,3*H*)-dione (13b)

^{13}C-NMR ($CDCl_3$) δ 154.6 (C-9a), 151.3 (C-4), 150.4 (C-2), 148.2 (C-10a), 137.7 (C-1, phenyl), 132.1 (C-6, phenyl), 131.0 (q, $^2J_{C,F}$= 32.3 Hz, C-3, phenyl), 129.1 (C-5, phenyl), 125.5 (q, $^3J_{C,F}$= 3.7 Hz, C-2, phenyl), 124.7 (q, $^3J_{C,F}$= 3.6 Hz, C-2, phenyl), 123.9 (q, $^1J_{C,F}$= 272.4 Hz, CF_3), 106.8 (C-4a), 61.3 (N8-CH_2), 51.1 (C-7), 48.7 (C-9), 44.2 (C-6), 28.9 (N1-CH_3).

8-(3-Chlorophenethyl)-1-methyl-6,7,8,9-tetrahydropyrazino[2,1-*f*]purine-2,4(1*H*,3*H*)-dione (13c)

^{13}C-NMR ($CDCl_3$) δ 154.9 (C-9a), 151.7 (C-4), 148.4 (C-2), 147.6 (C-10a), 141.3 (C-1, phenyl), 134.3 (C-3, phenyl), 129.8 (C-5, phenyl), 128.8 (C-6, phenyl), 126.8 (C-2, phenyl), 126.7 (C-4, phenyl), 106.7 (C-4a), 57.3 (N8-CH_2), 51.3 (C-7), 49.0 (C-9), 44.2 (C-6), 33.7 (N8-CH_2-$\underline{CH_2}$), 29.7 (N1-CH_3).

8-(3-Bromophenethyl)-1-methyl-6,7,8,9-tetrahydropyrazino[2,1-*f*]purine-2,4(1*H*,3*H*)-dione (13d)

^{13}C-NMR ($CDCl_3$) δ 155.0 (C-9a), 151.7 (C-4), 148.5 (C-2), 147.6 (C-10a), 141.5 (C-1, phenyl), 131.7 (C-6, phenyl), 130.0 (C-3, phenyl), 129.6 (C-5, phenyl), 127.3 (C-2, phenyl), 122.6 (C-4, phenyl), 106.5 (C-4a), 58.7 (N8-CH_2), 51.3 (C-7), 49.1 (C-9), 44.1 (C-6), 33.2 (N8-CH_2-$\underline{CH_2}$), 29.7 (N1-CH_3). ESI-MS: negative mode 404.0 $[M-H]^-$, positive mode 406.4 $[M+H]^+$.

1-Methyl-8-(3-(trifluoromethyl)phenethyl)-6,7,8,9-tetrahydropyrazino[2,1-*f*]purine-2,4(1*H*,3*H*)-dione (13e)

^{13}C-NMR ($CDCl_3$) δ 155.0 (C-9a), 151.7 (C-4), 148.5 (C-2), 147.6 (C-10a), 140.1 (C-1, phenyl), 132.1 (C-6, phenyl), 131.0 (C-3, phenyl), 128.9 (C-5, phenyl), 125.2 (C-2, phenyl), 123.4 (q, $^1J_{C,F}$= 271.2 Hz, CF_3), 123.0 (C-4, phenyl), 106.5 (C-4a), 58.6 (N8-CH_2), 51.3 (C-7), 49.1 (C-9), 44.1 (C-6), 33.7 (N8-CH_2-$\underline{CH_2}$), 29.7 (N1-CH_3).

8-(2,4-Dichlorophenethyl)-1-methyl-6,7,8,9-tetrahydropyrazino[2,1-*f*]purine-2,4(1*H*,3*H*)-dione (13f)

^{13}C-NMR ($CDCl_3$) δ 155.0 (C-9a), 151.7 (C-4), 148.5 (C-2), 147.6 (C-10a), 135.3 (C-1, phenyl), 134.6 (C-2, phenyl), 133.0 (C-4, phenyl), 131.5 (C-6, phenyl), 129.4 (C-3, phenyl), 127.3 (C-5, phenyl), 106.5 (C-4a), 56.9 (N8-CH_2), 51.1 (C-7), 49.0 (C-9), 44.1 (C-6), 30.6 (N8-CH_2-$\underline{CH_2}$), 29.7 (N1-CH_3).

8-(3,4-Dichlorophenethyl)-1-methyl-6,7,8,9-tetrahydropyrazino[2,1-*f*]purine-2,4(1*H*,3*H*)-dione (13g)

^{13}C-NMR ($CDCl_3$) δ 155.0 (C-9a), 151.7 (C-4), 149.5 (C-2), 147.6 (C-10a), 140.5 (C-1, phenyl), 131.4, 131.5 and 133.2 (C-2, C-3, C-4, C-5 and C-6, phenyl), 106.5 (C-4a), 58.3 (N8-CH_2), 51.1 (C-7), 49.0 (C-9), 44.1 (C-6), 32.6 (N8-CH_2-$\underline{CH_2}$), 29.7 (N1-CH_3).

8-(3,4-Dichlorophenethyl)-1-methyl-6,7,8,9-tetrahydropyrazino[2,1-*f*]purine-2,4(1*H*,3*H*)-dione (13h)

^{13}C-NMR (DMSO-d_6) δ 154.3 (C-9a), 150.9 (C-4), 149.5 (C-2), 147.7 (C-10a), 138.8 (C-1, phenyl), 131.0 (C-2, phenyl), 130.6 (C-5, phenyl), 130.5 (C-6, phenyl), 129.8 (C-3, phenyl), 129.0 (C-4, phenyl), 106.2 (C-4a), 58.6 (N8-CH_2), 50.3 (C-7), 47.7 (C-9), 43.6 (C-6), 28.4 (N1-CH_3).

8-(2-Bromobenzyl)-3-ethyl-1-methyl-6,7,8,9-tetrahydropyrazino[2,1-*f*]purine-2,4(1*H*,3*H*)-dione (14a)

^{13}C-NMR ($CDCl_3$) δ 154.9 (C-9a), 151.7 (C-4), 148.4 (C-2), 147.7 (C-10a), 135.6 (C-1, phenyl), 133.1 (C-3, phenyl), 131.0 (C-6, phenyl), 129.4 (C-4, phenyl), 127.6 (C-5, phenyl), 124.8 (C-2, phenyl), 106.6 (C-4a), 60.8 (N8-CH_2), 51.0 (C-7), 48.8 (C-9), 44.2 (C-6), 36.3 (N3-CH_2), 29.7 (N1-CH_3), 13.3 (N3-CH_2-$\underline{CH_3}$).

8-(3-Bromobenzyl)-3-ethyl-1-methyl-6,7,8,9-tetrahydropyrazino[2,1-*f*]purine-2,4(1*H*,3*H*)-dione (14b)

^{13}C-NMR ($CDCl_3$) δ 154.9 (C-9a), 151.7 (C-4), 148.4 (C-2), 147.7 (C-10a), 132.7 (C-1, phenyl), 132.2 (C-2, phenyl), 130.7 (C-4, phenyl), 128.5 (C-5, phenyl), 128.4 (C-6, phenyl), 123.1 (C-3, phenyl), 106.8 (C-4a), 60.7 (N8-CH_2), 50.1 (C-7), 48.5 (C-9), 42.9 (C-6), 36.3 (N3-CH_2), 29.7 (N1-CH_3), 13.3 (N3-CH_2-$\underline{CH_3}$).

8-(4-Bromobenzyl)-3-ethyl-1-methyl-6,7,8,9-tetrahydropyrazino[2,1-*f*]purine-2,4(1*H*,3*H*)-dione (14c)

^{13}C-NMR ($CDCl_3$) δ 154.9 (C-9a), 151.7 (C-4), 148.4 (C-2), 147.7 (C-10a), 135.6 (C-1, phenyl), 131.0 (C-3 and C-5, phenyl), 130.6 (C-2 and C-6, phenyl), 121.7 (C-4, phenyl), 106.6 (C-4a), 61.2 (N8-CH_2), 51.0 (C-7), 48.8 (C-9), 44.2 (C-6), 36.3 (N3-CH_2), 29.7 (N1-CH_3), 13.3 (N3-CH_2-$\underline{CH_3}$).

3-Ethyl-1-methyl-8-(2-(trifluoromethyl)benzyl)-6,7,8,9-tetrahydropyrazino[2,1-*f*]purine-2,4(1*H*,3*H*)-dione (14d)

^{13}C-NMR ($CDCl_3$) δ 160.3 (C-2, phenyl), 155.0 (C-9a), 151.7 (C-4), 148.5 (C-2), 147.6 (C-10a), 135.9 (C-1, phenyl), 132.1 (C-5, phenyl), 130.4 (C-6, phenyl), 128.9 (q, $^2J_{C,F}$= 30.3 Hz, C-2, phenyl), 127.6 (C-4, phenyl), 126.1 (q, $^3J_{C,F}$= 5.7 Hz, C-3, phenyl), 124.3 (q, $^1J_{C,F}$= 273.9 Hz, CF_3), 105.2 (C-4a), 61.1 (N8-CH_2), 50.8 (C-7), 49.0 (C-9), 44.1 (C-6), 36.3 (N3-CH_2), 29.7 (N1-CH_3), 13.3 (N3-CH_2-$\underline{CH_3}$).

3-Ethyl-1-methyl-8-(3-(trifluoromethyl)benzyl)-6,7,8,9-tetrahydropyrazino[2,1-*f*]purine-2,4(1*H*,3*H*)-dione (14e)

^{13}C-NMR ($CDCl_3$) δ 154.7 (C-9a), 151.3 (C-4), 148.5 (C-2), 147.6 (C-10a), 137.8 (C-1, phenyl), 132.1 (C-6, phenyl), 131.1 (q, $^2J_{C,F}$= 32.3 Hz, C-3, phenyl), 129.1 (C-5, phenyl), 125.5 (q, $^3J_{C,F}$= 3.7 Hz, C-

2, phenyl), 124.7 (q, $^{3}J_{C,F}$= 3.7 Hz, C-4, phenyl), 124.0 (q, $^{1}J_{C,F}$= 272.2 Hz, CF_3), 105.2 (C-4a), 57.4 (N8-CH_2), 50.8 (C-7), 49.0 (C-9), 44.1 (C-6), 36.3 (N3-CH_2), 29.7 (N1-CH_3), 13.3 (N3-CH_2-$\underline{CH_3}$).

3-Ethyl-1-methyl-8-(4-(trifluoromethyl)benzyl)-6,7,8,9-tetrahydropyrazino[2,1-*f*]purine-2,4(1*H*,3*H*)-dione (14f)

^{13}C-NMR ($CDCl_3$) δ 154.7 (C-9a), 151.3 (C-4), 148.5 (C-2), 147.6 (C-10a), 141.7 (C-1, phenyl), 130.2 (q, $^{2}J_{C,F}$= 32.6 Hz, C-4, phenyl), 129.1 (C-2 and C-6, phenyl), 125.6 (q, $^{3}J_{C,F}$= 3.7 Hz, C-3 and C-5, phenyl), 124.0 (q, $^{1}J_{C,F}$= 272.2 Hz, CF_3), 106.5 (C-4a), 57.0 (N8-CH_2), 50.1 (C-7), 48.5 (C-9), 42.9 (C-6), 36.3 (N3-CH_2), 29.7 (N1-CH_3), 13.3 (N3-CH_2-$\underline{CH_3}$).

3-Ethyl-8-(3-fluorobenzyl)-1-methyl-6,7,8,9-tetrahydropyrazino[2,1-*f*]purine-2,4(1*H*,3*H*)-dione (14g)

^{13}C-NMR ($CDCl_3$) δ 163.0 (d, $^{1}J_{C,F}$= 246.5 Hz, C-3, phenyl), 155.0 (C-9a), 151.7 (C-4), 148.5 (C-2), 147.6 (C-10a), 139.3 (d, $^{3}J_{C,F}$= 7.0 Hz, C-1, phenyl), 130.1 (d, $^{3}J_{C,F}$= 8.2 Hz, C-5, phenyl), 124.4 (d, $^{4}J_{C,F}$= 2.6 Hz, C-6, phenyl), 115.6 (d, $^{2}J_{C,F}$= 21.5 Hz, C-2, phenyl), 114.8 (d, $^{2}J_{C,F}$= 21.2 Hz, C-4, phenyl), 106.5 (C-4a), 61.2 (N8-CH_2), 51.2 (C-7), 48.8 (C-9), 44.3 (C-6), 36.3 (N3-CH_2), 29.7 (N1-CH_3), 13.3 (N3-CH_2-$\underline{CH_3}$).

3-Ethyl-8-(4-fluorobenzyl)-1-methyl-6,7,8,9-tetrahydropyrazino[2,1-*f*]purine-2,4(1*H*,3*H*)-dione (ABS 324) (14h)

^{13}C-NMR ($CDCl_3$) δ 162.4 (d, $^{1}J_{C,F}$= 246.2 Hz, C-4, phenyl), 154.7 (C-9a), 151.3 (C-4), 148.4 (C-2), 147.6 (C-10a), 132.3 (d, $^{4}J_{C,F}$= 3.1 Hz, C-1, phenyl), 130.5 (d, $^{3}J_{C,F}$= 8.0 Hz, C-2 and C-6, phenyl), 115.5 (d, $^{2}J_{C,F}$= 21.4 Hz, C-3 and C-5, phenyl), 106.6 (C-4a), 61.1 (N8-CH_2), 51.2 (C-7), 48.7 (C-9), 44.3 (C-6), 36.3 (N3-CH_2), 29.6 (N1-CH_3), 13.3 (N3-CH_2-$\underline{CH_3}$).

8-(3-Chlorobenzyl)-3-ethyl-1-methyl-6,7,8,9-tetrahydropyrazino[2,1-*f*]purine-2,4(1*H*,3*H*)-dione (14i)

^{13}C-NMR ($CDCl_3$) δ 154.9 (C-9a), 151.7 (C-4), 148.4 (C-2), 147.6 (C-10a), 141.7 (C-1, phenyl), 132.8 (C-3, phenyl), 130.0 (C-5, phenyl), 128.9 (C-6, phenyl), 125.5 (C-2, phenyl), 123.2 (C-4, phenyl), 106.5 (C-4a), 58.7 (N8-CH_2), 51.3 (C-7), 49.1 (C-9), 44.0 (C-6), 36.3 (N3-CH_2), 29.7 (N1-CH_3), 13.3 (N3-CH_2-$\underline{CH_3}$).

8-(2,5-Dichlorobenzyl)-3-ethyl-1-dimethyl-6,7,8,9-tetrahydropyrazino[2,1-*f*]purine-2,4(1*H*,3*H*)-dione (14j)

^{13}C-NMR ($CDCl_3$) δ 155.0 (C-9a), 151.7 (C-4), 148.5 (C-2), 147.6 (C-10a), 135.6 (C-1, phenyl), 133.1 (C-2, phenyl), 132.5 (C-5, phenyl), 130.9 (C-6, phenyl), 130.6 (C-3, phenyl), 129.2 (C-4, phenyl), 106.6 (C-4a), 57.9 (N8-CH_2), 51.0 (C-7), 48.9 (C-9), 44.1 (C-6), 36.3 (N3-CH_2), 29.7 (N1-CH_3), 13.3 (N3-CH_2-$\underline{CH_3}$).

8-(2,6-Dichlorobenzyl)-3-ethyl-1-methyl-6,7,8,9-tetrahydropyrazino[2,1-*f*]purine-2,4(1*H*,3*H*)-dione (14k)

^{13}C-NMR ($CDCl_3$) δ 155.0 (C-9a), 151.7 (C-4), 148.5 (C-2), 147.6 (C-10a), 137.0 (C-2 and C-6, phenyl), 133.4 (C-1, phenyl), 129.7 (C-4, phenyl), 128.6 (C-3 and C-5, phenyl), 106.5 (C-4a), 55.4 (N8-CH_2), 51.1 (C-7), 49.0 (C-9), 44.1 (C-6), 36.3 (N3-CH_2), 29.7 (N1-CH_3), 13.3 (N3-CH_2-$\underline{CH_3}$).

3-Ethyl-8-(2-fluoro-3-(trifluoromethyl)benzyl)-1-methyl-6,7,8,9-tetrahydropyrazino[2,1-*f*]purine-2,4(1*H*,3*H*)-dione (14l)

^{13}C-NMR ($CDCl_3$) δ 158.2 (d, $^{1}J_{C,F}$= 254.4 Hz, C-2, phenyl), 155.0 (C-9a), 151.7 (C-4), 148.5 (C-2), 147.6 (C-10a), 134.9 (d, $^{3}J_{C,F}$= 4.3 Hz, C-6, phenyl), 126.8 (d, $^{4}J_{C,F}$= 3.6 Hz, C-5, phenyl), 125.3 (d, $^{2}J_{C,F}$= 13.6 Hz, C-1, phenyl), 124.1 (d, $^{3}J_{C,F}$= 4.4 Hz, C-4, phenyl), 122.5 (q, $^{3}J_{C,F}$= 272.3 Hz, CF_3), 118.9-118.3 (m, C-3, phenyl), 106.5 (C-4a), 53.5 (N8-CH_2), 50.5 (C-7), 48.8 (C-9), 43.7 (C-6), 36.3 (N3-CH_2), 29.7 (N1-CH_3), 13.3 (N3-CH_2-$\underline{CH_3}$).

8-(2-Chloro-5-(trifluoromethyl)benzyl)-3-ethyl-1-methyl-6,7,8,9-tetrahydropyrazino[2,1-*f*]purine-2,4(1*H*,3*H*)-dione (14m)

^{13}C-NMR ($CDCl_3$) δ 154.7 (C-9a), 151.4 (C-4), 148.5 (C-2), 147.4 (C-10a), 138.1 (C-2, phenyl), 135.6 (C-1, phenyl), 130.3 (C-5, phenyl), 129.6 (q, $^{2}J_{C,F}$= 33.0 Hz, C-3, phenyl), 127.3 (q, $^{3}J_{C,F}$= 3.6 Hz, C-6, phenyl), 125.7 (q, $^{3}J_{C,F}$= 3.6 Hz, C-4, phenyl), 123.6 (q, $^{1}J_{C,F}$= 272.7 Hz, CF_3), 106.8 (C-4a), 58.0 (N8-CH_2), 51.3 (C-7), 49.0 (C-9), 44.2 (C-6), 36.3 (N3-CH_2), 29.7 (N1-CH_3), 13.3 (N3-CH_2-$\underline{CH_3}$).

8-(5-Bromo-2-fluorobenzyl)-3-ethyl-1-methyl-6,7,8,9-tetrahydropyrazino[2,1-*f*]purine-2,4(1*H*,3*H*)-dione (14o)

^{13}C-NMR ($CDCl_3$) δ 160.3 (d, $^{1}J_{C,F}$= 247.5 Hz, C-2, phenyl), 154.6 (C-9a), 151.3 (C-4), 148.4 (C-2), 147.5 (C-10a), 133.8 ($^{3}J_{C,F}$= 7.6 Hz, C-6, phenyl), 132.4 ($^{3}J_{C,F}$= 8.3 Hz, C-4, phenyl), 125.6 ($^{2}J_{C,F}$= 15.7 Hz, C-1, phenyl), 117.4 ($^{2}J_{C,F}$= 23.8 Hz, C-3, phenyl), 116.9 ($^{4}J_{C,F}$= 3.2 Hz, C-5, phenyl), 106.7 (C-4a), 53.8 (N8-CH_2), 50.9 (C-7), 48.8 (C-9), 44.2 (C-6), 36.3 (N3-CH_2), 29.6 (N1-CH_3), 13.3 (N3-CH_2-$\underline{CH_3}$).

3-Ethyl-8-(3-fluoro-5-(trifluoromethyl)benzyl)-1-methyl-6,7,8,9-tetrahydropyrazino[2,1-*f*]purine-2,4(1*H*,3*H*)-dione (14p)

^{13}C-NMR ($CDCl_3$) δ 162.7 (d, $^{1}J_{C,F}$= 249.7 Hz, C-2, phenyl), 154.7 (C-9a), 151.3 (C-4), 148.5 (C-2), 147.3 (C-10a), 140.9 (d, $^{3}J_{C,F}$= 7.3 Hz, C-1, phenyl), 132.8 (dq, $^{3}J_{C,F}$= 3.8 Hz, $^{2}J_{C,F}$= 33.4 Hz, C-5, phenyl), 121.1 (q, $^{3}J_{C,F}$= 3.3 Hz, C-6, phenyl), 120.9 (q, $^{1}J_{C,F}$= 272.7 Hz, CF_3), 119.0 (d, $^{2}J_{C,F}$= 21.5 Hz, C-2, phenyl), 112.4 (d, $^{2}J_{C,F}$= 24.5 Hz, C-4, phenyl), 106.8 (C-4a), 60.9 (N8-CH_2), 51.2 (C-7), 48.9 (C-9), 44.2 (C-6), 36.4 (N3-CH_2), 29.6 (N1-CH_3), 13.3 (N3-CH_2-$\underline{CH_3}$).

8-(3,5-Bis(trifluoromethyl)benzyl)-3-ethyl-1-methyl-6,7,8,9-tetrahydropyrazino[2,1-*f*]purine-2,4(1*H*,3*H*)-dione (14q)

^{13}C-NMR ($CDCl_3$) δ 155.0 (C-9a), 151.3 (C-4), 148.4 (C-2), 147.1 (C-10a), 139.6 (C-1, phenyl), 132.1 (q, $^{2}J_{C,F}$= 33.4 Hz, C-3 and C-5, phenyl), 128.7 (C-2 and C-6, phenyl), 123.2 (q, $^{1}J_{C,F}$= 272.8 Hz, 2 × CF_3), 121.9 (q, $^{3}J_{C,F}$= 3.6 Hz, C-4, phenyl), 106.8 (C-4a), 60.8 (N8-CH_2), 51.3 (C-7), 48.9 (C-9), 44.1 (C-6), 36.4 (N3-CH_2), 29.6 (N1-CH_3), 13.3 (N3-CH_2-$\underline{CH_3}$).

8-(3-Chlorobenzyl)-1,3-diethyl-6,7,8,9-tetrahydropyrazino[2,1-*f*]purine-2,4(1*H*,3*H*)-dione (15c)

^{13}C-NMR ($CDCl_3$) δ 154.8 (C-9a), 151.7 (C-4), 148.0 (C-2), 147.7 (C-10a), 138.8 (C-1, phenyl), 134.6 (C-3, phenyl), 129.9 (C-5, phenyl), 129.0 (C-6, phenyl), 128.0 (C-2, phenyl), 127.0 (C-4, phenyl), 106.8 (C-4a), 61.4 (N8-CH_2), 51.3 (C-7), 48.9 (C-9), 44.3 (C-6), 38.5 (N1-CH_2), 36.3 (N3-CH_2), 13.4 (N1-CH_2-$\underline{CH_3}$), 13.3 (N3-CH_2-$\underline{CH_3}$).

8-(3-Bromobenzyl)-1,3-diethyl-6,7,8,9-tetrahydropyrazino[2,1-*f*]purine-2,4(1*H*,3*H*)-dione (15d)

^{13}C-NMR ($CDCl_3$) δ 154.9 (C-9a), 151.7 (C-4), 148.4 (C-2), 147.7 (C-10a), 132.7 (C-1, phenyl), 132.2 (C-2, phenyl), 130.7 (C-4, phenyl), 128.5 (C-5, phenyl), 128.4 (C-6, phenyl), 123.1 (C-3, phenyl), 106.8 (C-4a), 60.7 (N8-CH_2), 50.1 (C-7), 48.5 (C-9), 42.9 (C-6), 38.5 (N1-CH_2), 36.3 (N3-CH_2), 14.0 (N1-CH_2-$\underline{CH_3}$), 13.3 (N3-CH_2-$\underline{CH_3}$).

8-(4-Bromobenzyl)-1,3-diethyl-6,7,8,9-tetrahydropyrazino[2,1-*f*]purine-2,4(1*H*,3*H*)-dione (15e)

^{13}C-NMR ($CDCl_3$) δ 154.9 (C-9a), 151.7 (C-4), 148.4 (C-2), 147.7 (C-10a), 135.6 (C-1, phenyl), 131.0 (C-3 and C-5, phenyl), 130.6 (C-2 and C-6, phenyl), 121.7 (C-4, phenyl), 106.6 (C-4a), 61.2 (N8-CH_2), 51.0 (C-7), 48.8 (C-9), 44.2 (C-6), 38.5 (N1-CH_2), 36.3 (N3-CH_2), 13.4 (N1-CH_2-$\underline{CH_3}$), 13.3 (N3-CH_2-$\underline{CH_3}$).

1,3-Diethyl-8-(2-(trifluoromethyl)benzyl)-6,7,8,9-tetrahydropyrazino[2,1-*f*]purine-2,4(1*H*,3*H*)-dione (15f)

^{13}C-NMR ($CDCl_3$) δ 160.3 (C-2, phenyl), 154.8 (C-9a), 150.8 (C-4), 148.0 (C-2), 147.8 (C-10a), 135.9 (C-1, phenyl), 132.1 (C-5, phenyl), 130.5 (C-6, phenyl), 128.9 (q, $^2J_{C,F}$= 30.4 Hz, C-2, phenyl), 126.1 (q, $^3J_{C,F}$= 5.7 Hz, C-3, phenyl), 127.7 (C-4, phenyl), 124.3 (q, $^1J_{C,F}$= 273.8 Hz, CF_3), 106.9 (C-4a), 57.4 (N8-CH_2), 51.5 (C-7), 49.1 (C-9), 44.4 (C-6), 38.5 (N1-CH_2), 36.3 (N3-CH_2), 13.4 (N1-CH_2-$\underline{CH_3}$), 13.3 (N3-CH_2-$\underline{CH_3}$).

1,3-Diethyl-8-(3-(trifluoromethyl)benzyl)-6,7,8,9-tetrahydropyrazino[2,1-*f*]purine-2,4(1*H*,3*H*)-dione (15g)

^{13}C-NMR ($CDCl_3$) δ 154.8 (C-9a), 150.7 (C-4), 147.9 (C-2), 147.6 (C-10a), 137.8 (C-1, phenyl), 132.2 (C-6, phenyl), 131.8 (q, $^2J_{C,F}$= 32.3 Hz, C-3, phenyl), 129.1 (C-5, phenyl), 125.5 (q, $^3J_{C,F}$= 3.7 Hz, C-2, phenyl), 124. (q, $^3J_{C,F}$= 3.7 Hz, C-4, phenyl), 124.0 (q, $^1J_{C,F}$= 272.4 Hz, CF_3), 106.8 (C-4a), 61.4 (N8-CH_2), 51.3 (C-7), 49.0 (C-9), 44.2 (C-6), 38.5 (N1-CH_2), 36.3 (N3-CH_2), 13.4 (N1-CH_2-$\underline{CH_3}$), 13.3 (N3-CH_2-$\underline{CH_3}$).

1,3-Diethyl-8-(4-fluoro-3-(trifluoromethyl)benzyl)-6,7,8,9-tetrahydropyrazino[2,1-*f*]purine-2,4(1*H*,3*H*)-dione (15i)

^{13}C-NMR ($CDCl_3$) δ 159.2 (d, $^1J_{C,F}$= 255.3 Hz, C-4, phenyl), 154.8 (C-9a), 150.7 (C-4), 148.0 (C-2), 147.4 (C-10a), 134.1 (d, $^3J_{C,F}$= 8.1 Hz, C-6, phenyl), 133.0 (d, $^4J_{C,F}$= 3.8 Hz, C-1, phenyl), 127.4 (q, $^3J_{C,F}$= 3.4 Hz, C-2, phenyl), 122.4 (q, $^1J_{C,F}$= 272.9 Hz, CF_3), 118.0 (q, $^2J_{C,F}$= 27.3 Hz, C-3, phenyl), 117.2 (d, $^2J_{C,F}$= 20.8 Hz, C-5, phenyl), 106.5 (C-4a), 60.7 (N8-CH_2), 51.2 (C-7), 48.9 (C-9), 44.1 (C-6), 38.5 (N1-CH_2), 36.3 (N3-CH_2), 13.4 (N1-CH_2-$\underline{CH_3}$), 13.3 (N3-CH_2-$\underline{CH_3}$).

1,3-Diethyl-8-(2-Fluoro-5-(trifluoromethyl)benzyl)-6,7,8,9-tetrahydropyrazino[2,1-*f*]purine-2,4(1*H*,3*H*)-dione (15j)

^{13}C-NMR ($CDCl_3$) δ 163.0 (d, $^1J_{C,F}$= 252.6 Hz, C-2, phenyl), 154.8 (C-9a), 150.8 (C-4), 148.0 (C-2), 147.4 (C-10a), 128.4 (q, $^3J_{C,F}$= 2.9 Hz, C-6, phenyl), 127.3 (dq, $^3J_{C,F}$= 9.3 Hz, $^3J_{C,F}$= 3.7 Hz, C-4, phenyl), 124.0 (q, $^1J_{C,F}$= 270.4 Hz, CF_3), 125.3 (dq, $^4J_{C,F}$= 2.5 Hz, $^2J_{C,F}$= 25.8 Hz, C-5, phenyl), 124.6 (d, $^2J_{C,F}$= 15.4 Hz, C-1, phenyl), 116.3 (q, $^2J_{C,F}$= 23.5 Hz, C-3, phenyl), 106.9 (C-4a), 54.0 (N8-CH_2), 51.1 (C-7), 49.0 (C-9), 44.2 (C-6), 38.5 (N1-CH_2), 36.3 (N3-CH_2), 13.4 (N1-CH_2-$\underline{CH_3}$), 13.3 (N3-CH_2-$\underline{CH_3}$).

8-(3-Bromo-4-fluorobenzyl)-1,3-diethyl-6,7,8,9-tetrahydropyrazino[2,1-*f*]purine-2,4(1*H*,3*H*)-dione (15k)

^{13}C-NMR ($CDCl_3$) δ 158.6 (d, $^{1}J_{C,F}$= 247.8 Hz, C-4, phenyl), 154.8 (C-9a), 150.7 (C-4), 148.0 (C-2), 147.6 (C-10a), 134.2 (d, $^{4}J_{C,F}$= 3.8 Hz, C-1, phenyl), 133.8 (C-2, phenyl), 129.3 (d, $^{3}J_{C,F}$= 7.1 Hz, C-6, phenyl), 116.5 (d, $^{2}J_{C,F}$= 22.4 Hz, C-5, phenyl), 109.3 (d, $^{2}J_{C,F}$= 21.1 Hz, C-3, phenyl), 106.8 (C-4a), 60.6 (N8-CH_2), 51.3 (C-7), 48.9 (C-9), 44.2 (C-6), 38.5 (N1-CH_2), 36.3 (N3-CH_2), 13.4 (N1-CH_2-$\underline{CH_3}$), 13.3 (N3-CH_2-$\underline{CH_3}$).

8-(5-Bromo-2-fluorobenzyl)-1,3-diethyl-6,7,8,9-tetrahydropyrazino[2,1-*f*]purine-2,4(1*H*,3*H*)-dione (15l)

^{13}C-NMR ($CDCl_3$) δ 160.3 (d, $^{1}J_{C,F}$= 247.5 Hz, C-2, phenyl), 154.8 (C-9a), 150.7 (C-4), 148.0 (C-2), 147.5 (C-10a), 133.8 (d, $^{3}J_{C,F}$= 4.1 Hz, C-6, phenyl), 132.5 (d, $^{3}J_{C,F}$= 8.3 Hz, C-4, phenyl), 125.7 (d, $^{2}J_{C,F}$= 15.6 Hz, C-1, phenyl), 117.4 (d, $^{2}J_{C,F}$= 23.8 Hz, C-3, phenyl), 116.9 (d, $^{4}J_{C,F}$= 3.3 Hz, C-5, phenyl), 106.8 (C-4a), 54.0 (N8-CH_2), 51.0 (C-7), 48.9 (C-9), 44.3 (C-6), 38.5 (N1-CH_2), 36.3 (N3-CH_2), 13.4 (N1-CH_2-$\underline{CH_3}$), 13.3 (N3-CH_2-$\underline{CH_3}$).

8-(3,5-Dichlorobenzyl)-1,3-diethyl-6,7,8,9-tetrahydropyrazino[2,1-f]purine-2,4(1*H*,3*H*)-dione (15m)

^{13}C-NMR ($CDCl_3$) δ 154.8 (C-9a), 150.7 (C-4), 148.0 (C-2), 147.4 (C-10a), 140.2 (C-1, phenyl), 135.3 (C-3 and C-5, phenyl), 128.0 (C-4, phenyl), 127.2 (C-2 and C-6, phenyl), 106.8 (C-4a), 60.8 (N8-CH_2), 51.3 (C-7), 49.0 (C-9), 44.2 (C-6), 38.5 (N1-CH_2), 36.3 (N3-CH_2), 13.4 (N1-CH_2-$\underline{CH_3}$), 13.3 (N3-CH_2-$\underline{CH_3}$).

8-(2-Chloro-5-(trifluoromethyl)benzyl)-1,3-diethyl-6,7,8,9-tetrahydropyrazino[2,1-*f*]purine-2,4(1*H*,3*H*)-dione (15n)

^{13}C-NMR ($CDCl_3$) δ 154.8 (C-9a), 150.7 (C-4), 148.0 (C-2), 147.4 (C-10a), 138.1 (C-2, phenyl), 135.6 (C-1, phenyl), 130.3 (C-5, phenyl), 129.6 (q, $^{2}J_{C,F}$= 33.1 Hz, C-3, phenyl), 127.3 (q, $^{3}J_{C,F}$= 3.7 Hz, C-6, phenyl), 125.8 (q, $^{3}J_{C,F}$= 3.5 Hz, C-4, phenyl), 123.6 (q, $^{1}J_{C,F}$= 272.4 Hz, CF_3), 106.9 (C-4a), 58.0 (N8-CH_2), 51.3 (C-7), 49.1 (C-9), 44.2 (C-6), 38.5 (N1-CH_2), 36.3 (N3-CH_2), 13.4 (N1-CH_2-$\underline{CH_3}$), 13.3 (N3-CH_2-$\underline{CH_3}$).

8-(4-Chloro-2-(trifluoromethyl)benzyl)-1,3-diethyl-6,7,8,9-tetrahydropyrazino[2,1-*f*]purine-2,4(1*H*,3*H*)-dione (15o)

^{13}C-NMR ($CDCl_3$) δ 155.0 (C-9a), 150.7 (C-4), 148.0 (C-2), 147.5 (C-10a), 134.5 (C-4, phenyl), 133.7 (C-1, phenyl), 132.2 (C-5, phenyl), 132.0 (C-6, phenyl), 130.4 (q, $^{2}J_{C,F}$= 31.1 Hz, C-2, phenyl), 126.4 (q, $^{3}J_{C,F}$= 5.9 Hz, C-3, phenyl), 123.5 (q, $^{1}J_{C,F}$= 274.3 Hz, CF_3), 106.9 (C-4a), 56.9 (N8-CH_2), 51.5 (C-7), 49.0 (C-9), 44.4 (C-6), 38.5 (N1-CH_2), 36.3 (N3-CH_2), 13.4 (N1-CH_2-$\underline{CH_3}$), 13.3 (N3-CH_2-$\underline{CH_3}$).

8-(4-Chloro-3-(trifluoromethyl)benzyl)-1,3-diethyl-6,7,8,9-tetrahydropyrazino[2,1-*f*]purine-2,4(1*H*,3*H*)-dione (15p)

^{13}C-NMR ($CDCl_3$) δ 154.8 (C-9a), 150.8 (C-4), 148.0 (C-2), 147.3 (C-10a), 136.1 (C-1, phenyl), 133.1 (C-6, phenyl), 131.8 (C-5, phenyl), 131.7 (C-4, phenyl), 128.7 (q, $^{2}J_{C,F}$= 31.6 Hz, C-3, phenyl), 127.8 (q, $^{3}J_{C,F}$= 5.3 Hz, C-2, phenyl), 122.7 (q, $^{1}J_{C,F}$= 273.3 Hz, CF_3), 106.9 (C-4a), 60.8 (N8-CH_2), 51.4 (C-7), 49.0 (C-9), 44.2 (C-6), 38.5 (N1-CH_2), 36.3 (N3-CH_2), 13.4 (N1-CH_2-$\underline{CH_3}$), 13.3 (N3-CH_2-$\underline{CH_3}$).

8-(3,5-Bis(trifluoromethyl)benzyl)-1,3-diethyl-6,7,8,9-tetrahydropyrazino[2,1-*f*]purine-2,4(1*H*,3*H*)-dione (15q)

^{13}C-NMR ($CDCl_3$) δ 154.8 (C-9a), 150.8 (C-4), 148.0 (C-2), 147.1 (C-10a), 139.7 (C-1, phenyl), 132.1 (q, $^{2}J_{C,F}$= 33.4 Hz, C-3 and C-5, phenyl), 128.8 (q, $^{3}J_{C,F}$= 2.8 Hz, C-2 and C-6, phenyl), 123.2 (q, $^{1}J_{C,F}$= 272.9 Hz, 2 × CF_3), 122.0 (q, $^{3}J_{C,F}$= 3.6 Hz, C-4, phenyl), 106.9 (C-4a), 60.8 (N8-CH_2), 51.3 (C-7), 49.1 (C-9), 44.2 (C-6), 38.6 (N1-CH_2), 36.3 (N3-CH_2), 13.4 (N1-CH_2-$\underline{CH_3}$), 13.3 (N3-CH_2-$\underline{CH_3}$).

1,3-Diethyl-8-(3,4,5-trifluorobenzyl)-6,7,8,9-tetrahydropyrazino[2,1-*f*]purine-2,4(1*H*,3*H*)-dione (15r)

^{13}C-NMR ($CDCl_3$) δ 154.8 (C-9a), 151.4 (ddd, $^{1}J_{C,F}$= 250.5 Hz, $^{2}J_{C,F}$= 10.0 Hz, $^{3}J_{C,F}$= 3.7 Hz, C-3 and C-5, phenyl), 150.7 (C-4), 148.0 (C-2), 147.3 (C-10a), 139.3 (dd, $^{1}J_{C,F}$= 251.8 Hz, $^{2}J_{C,F}$= 15.6 Hz, C-4, phenyl), 133.4 (C-1, phenyl), 112.5 (dd, $^{2}J_{C,F}$= 16.4 Hz, $^{3}J_{C,F}$= 4.9 Hz, C-2 and C-6, phenyl), 106.8 (C-4a), 60.6 (N8-CH_2), 51.3 (C-7), 48.9 (C-9), 44.2 (C-6), 38.5 (N1-CH_2), 36.3 (N3-CH_2), 13.4 (N1-CH_2-$\underline{CH_3}$), 13.3 (N3-CH_2-$\underline{CH_3}$).

8-(3,4-Dichlorobenzyl)-1-ethyl-3-methyl-6,7,8,9-tetrahydropyrazino[2,1-*f*]purine-2,4(1*H*,3*H*)-dione (16a)

^{13}C-NMR ($CDCl_3$) δ 155.0 (C-9a), 151.1 (C-4), 148.0 (C-2), 147.6 (C-10a), 137.0 (C-1, phenyl), 132.8 (C-3, phenyl), 131.8 (C-4, phenyl), 130.7 and 130.6 (C-2 and C-5, phenyl), 128.1 (C-6, phenyl), 106.7 (C-4a), 60.7 (N8-CH_2), 51.3 (C-7), 48.9 (C-9), 44.2 (C-6), 38.6 (N1-CH_2), 27.7 (N3-CH_3), 13.3 (N1-CH_2-$\underline{CH_3}$).

8-(3,5-Dichlorobenzyl)-1-ethyl-3-methyl-6,7,8,9-tetrahydropyrazino[2,1-f]purine-2,4(1*H*,3*H*)-dione (16b)

^{13}C-NMR ($CDCl_3$) δ 155.1 (C-9a), 151.2 (C-4), 148.0 (C-2), 147.4 (C-10a), 140.2 (C-1, phenyl), 135.3 (C-3 and C-5, phenyl), 128.0 (C-4, phenyl), 127.2 (C-2 and C-6, phenyl), 106.7 (C-4a), 60.9 (N8-CH_2), 51.3 (C-7), 49.0 (C-9), 44.2 (C-6), 38.6 (N1-CH_2), 27.8 (N3-CH_3), 13.4 (N1-CH_2-$\underline{CH_3}$).

8-(3,4-Dichlorobenzyl)-1-cyclopropyl-3-methyl-6,7,8,9-tetrahydropyrazino[2,1-*f*]purine-2,4(1*H*,3*H*)-dione (17)

^{13}C-NMR ($CDCl_3$) δ 155.0 (C-9a), 151.7 (C-4), 149.5 (C-2), 147.6 (C-10a), 137.0 (C-1, phenyl), 132.8 (C-3, phenyl), 131.9 (C-4, phenyl), 130.7 and 130.6 (C-2 and C-5, phenyl), 128.1 (C-6, phenyl), 106.8 (C-4a), 60.7 (N8-CH_2), 51.3 (C-7), 48.8 (C-9), 44.3 (C-6), 27.7 (N3-CH_3), 26.1 (C-1, cyclopropyl), 8.1 (C-2 and C-3, cyclopropyl).

8-(3,4-Dichlorobenzyl)-1-methyl-3-propargyl-6,7,8,9-tetrahydropyrazino[2,1-*f*]purine-2,4(1*H*,3*H*)-dione (18)

^{13}C-NMR ($CDCl_3$) δ 154.9 (C-9a), 151.5 (C-4), 148.5 (C-2), 147.5 (C-10a), 137.0 (C-1, phenyl), 132.8 (C-3, phenyl), 131.8 (C-4, phenyl), 130.7 (C-2, phenyl), 130.6 (C-5, phenyl), 128.1 (C-6, phenyl), 106.7 (C-4a), 60.6 (N8-CH_2), 51.3 (C-7), 48.8 (C-9), 44.2 (C-6), 42.8 (N3-CH_2), 29.7 (N1-CH_3), 21.3 (N3-CH_2-$\underline{CH_2}$), 11.3 (N3-CH_2-CH_2-$\underline{CH_3}$).

8-(3,4-Dichlorobenzyl)-1-methyl-3-propargyl-6,7,8,9-tetrahydropyrazino[2,1-*f*]purine-2,4(1*H*,3*H*)-dione (19a)

^{13}C-NMR ($CDCl_3$) δ 153.8 (C-9a), 150.8 (C-4), 148.9 (C-2), 148.0 (C-10a), 136.9 (C-1, phenyl), 132.8 (C-2, phenyl), 131.9 (C-5, phenyl), 130.7 (C-6, phenyl), 130.6 (C-3, phenyl), 128.0 (C-4, phenyl), 106.5 (C-4a), 78.7 (N3-CH_2-$\underline{C}$≡CH), 70.4 (N3-CH_2-C≡$\underline{CH}$), 60.6 (N8-CH_2), 51.2 (C-7), 48.8 (C-9), 44.3 (C-6), 30.6 (N3-CH_2), 29.8 (N1-CH_3).

8-(2-Chloro-5-(trifluoromethyl)benzyl)-1-methyl-3-propargyl-6,7,8,9-tetrahydropyrazino[2,1-*f*]purine-2,4(1*H*,3*H*)-dione (19b)

^{13}C-NMR ($CDCl_3$) δ 153.8 (C-9a), 150.9 (C-4), 148.8 (C-2), 148.0 (C-10a), 138.1 (C-2, phenyl), 135.5 (C-1, phenyl), 130.4 (C-5, phenyl), 129.8 (q, $^2J_{C,F}$= 33.3 Hz, C-3, phenyl), 127.3 (q, $^3J_{C,F}$= 3.5 Hz, C-6, phenyl), 125.8 (q, $^3J_{C,F}$= 3.4 Hz, C-4, phenyl), 123.6 (q, $^1J_{C,F}$= 272.3 Hz, CF_3), 106.6 (C-4a), 78.7 (N3-CH_2-C≡$\underline{CH}$), 70.4 (N3-CH_2-$\underline{C}$), 57.9 (N8-CH_2), 51.2 (C-7), 48.9 (C-9), 44.3 (C-6), 30.4 (N3-CH_2), 29.8 (N1-CH_3).

1-Ethyl-8-(4-fluorophenyl)-3-propargyl-6,7,8,9-tetrahydropyrazino[2,1-*f*]purine-2,4(1*H*,3*H*)-dione (20b)

^{13}C-NMR ($CDCl_3$) δ 159.0 (C-9a), 157.1 (C-4), 155.7 (d, $^1J_{C,F}$= 246.3 Hz, C-4, phenyl), 151.7 (C-2), 148.4 (C-10a), 147.7 (C-1, phenyl), 118.7 (d, $^3J_{C,F}$= 7.0 Hz, C-2 and C-6, phenyl), 116.3 (d, $^2J_{C,F}$= 20.4 Hz, C-2 and C-6, phenyl), 105.9 (C-4a), 78.8 (N3-CH_2-$\underline{C}$≡CH), 70.3 (N3-CH_2-C≡$\underline{CH}$), 48.9 (C-7), 47.1 (C-9), 44.3 (C-6), 38.9 (N1-CH_2), 30.3 (N3-CH_2), 13.4 (N1-CH_2–$\underline{CH_3}$).

1-Ethyl-8-(3-methoxyphenyl)-3-propargyl-6,7,8,9-tetrahydropyrazino[2,1-*f*]purine-2,4(1*H*,3*H*)-dione (20c)

^{13}C-NMR ($CDCl_3$) δ 160.9 (C-3, phenyl), 154.0 (C-9a), 150.3 (C-4), 150.2 (C-2), 148.5 (C-10a), 147.8 (C-1, phenyl), 130.4 (C-5, phenyl), 108.9 (C-4, phenyl), 106.7 (C-6, phenyl), 106.0 (C-4a), 103.0 (C-2, phenyl), 78.8 (N3-CH_2-$\underline{C}$≡CH), 70.4 (N3-CH_2-C≡$\underline{CH}$), 55.3 (OCH_3), 48.0 (C-7), 46.4 (C-9), 44.0 (C-6), 38.9 (N1-CH_2), 30.3 (N3-CH_2), 13.4 (N1-CH_2–$\underline{CH_3}$).

8-(3,4-Dimethoxyphenyl)-1-ethyl-3-propargyl-6,7,8,9-tetrahydropyrazino[2,1-f]purine-2,4(1*H*,3*H*)-dione (20d)

^{13}C-NMR ($CDCl_3$) δ 154.0 (C-9a), 150.3 (C-4), 149.9 (C-2), 148.4 and 148.0 (C-3 and C-4, phenyl), 143.6 (C-10a), 129.2 (C-1, phenyl), 112.1 (C-6, phenyl), 108.6 (C-5, phenyl), 106.5 (C-4a), 103.2 (C-2, phenyl), 78.8 (N3-CH_2-$\underline{C}$≡CH), 70.4 (N3-CH_2-C≡$\underline{CH}$), 56.3 and 56.1 (2 × OCH_3), 49.4 (C-7), 47.9 (C-9), 44.2 (C-6), 38.9 (N1-CH_2), 30.3 (N3-CH_2), 13.4 (N1-CH_2–$\underline{CH_3}$).

8-Benzyl-1-ethyl-3-propargyl-6,7,8,9-tetrahydropyrazino[2,1-*f*]purine-2,4(1*H*,3*H*)-dione (20e)

^{13}C-NMR ($CDCl_3$) δ 153.7 (C-9a), 149.9 (C-4), 148.2 (C-2), 141.4 (C-10a), 131.4 (C-3 and C-5, phenyl), 130.7 (C-1, phenyl), 129.6 (C-2 and C-6, phenyl), 127.0 (C-4, phenyl), 107.0 (C-4a), 78.4 (N3-CH_2-$\underline{C}$≡CH), 70.8 (N3-CH_2-C≡$\underline{CH}$), 60.0 (N8-CH_2), 47.7 (C-7), 47.6 (C-9), 40.9 (C-6), 39.0 (N1-CH_2), 30.4 (N3-CH_2), 13.2 (N1-CH_2–$\underline{CH_3}$).

1-Ethyl-8-(2-methoxybenzyl)-3-propargyl-6,7,8,9-tetrahydropyrazino[2,1-*f*]purine-2,4(1*H*,3*H*)-dione (20f)

^{13}C-NMR ($CDCl_3$) δ 157.9 (C-2, phenyl), 153.9 (C-9a), 150.3 (C-4), 150.0 (C-2), 148.3 (C-10a), 130.2 (C-4, phenyl), 129.0 (C-6, phenyl), 124.3 (C-1, phenyl), 120.5 (C-5, phenyl), 110.7 (C-3, phenyl), 106.5 (C-4a), 78.9 (N3-CH_2-$\underline{C}$≡CH), 70.2 (N3-CH_2-C≡$\underline{CH}$), 58.5 (N8-CH_2), 55.2 (OCH_3), 51.1 (C-7), 48.8 (C-9), 44.4 (C-6), 38.7 (N1-CH_2), 30.2 (N3-CH_2), 13.4 (N1-CH_2–$\underline{CH_3}$).

1-Ethyl-8-(3-methoxybenzyl)-3-propargyl-6,7,8,9-tetrahydropyrazino[2,1-*f*]purine-2,4(1*H*,3*H*)-dione (20g)

^{13}C-NMR ($CDCl_3$) δ 159.9 (C-3, phenyl), 153.9 (C-9a), 150.3 (C-4), 148.6 (C-2), 148.4 (C-10a), 138.1 (C-1, phenyl), 129.6 (C-5, phenyl), 120.7 (C-6, phenyl), 114.5 (C-4, phenyl), 113.2 (C-2, phenyl), 106.6 (C-4a), 78.8 (N3-CH_2-$\underline{C}$≡CH), 70.3 (N3-CH_2-C≡$\underline{CH}$), 61.9 (N8-CH_2), 55.2 (OCH_3), 51.4 (C-7), 48.8 (C-9), 44.4 (C-6), 38.8 (N1-CH_2), 30.3 (N3-CH_2), 13.4 (N1-CH_2–$\underline{CH_3}$).

1-Ethyl-8-phenethyl-3-propargyl-6,7,8,9-tetrahydropyrazino[2,1-*f*]purine-2,4(1*H*,3*H*)-dione (20h)

^{13}C-NMR ($CDCl_3$) δ 153.9 (C-9a), 150.1 (C-4), 148.4 (C-2), 143.8 (C-10a), 136.5 (C-1, phenyl), 129.0 and 128.6 (C-2, C-3, C-5 and C-6, phenyl), 127.3 (C-4, phenyl), 106.9 (C-4a), 78.4 (N3-CH_2-$\underline{C}$≡CH), 70.6 (N3-CH_2-C≡$\underline{CH}$), 58.0 (N8-CH_2), 49.6 (C-7), 48.3 (C-9), 41.8 (C-6), 39.0 (N1-CH_2), 31.7 (N8-CH_2-$\underline{CH_2}$), 30.4 (N3-CH_2), 13.3 (N1-CH_2–$\underline{CH_3}$).

1-Ethyl-8-(2-methoxyphenethyl)-3-propargyl-6,7,8,9-tetrahydropyrazino[2,1-*f*]purine-2,4(1*H*,3*H*)-dione (20i)

^{13}C-NMR ($CDCl_3$) δ 157.4 (C-2, phenyl), 155.0 (C-9a), 153.9 (C-4), 150.2 (C-2), 148.5 (C-10a), 130.2 (C-2 and C-4, phenyl), 127.5 (C-1, phenyl), 120.6 (C-5, phenyl), 110.3 (C-3, phenyl), 106.5 (C-4a), 78.7 (N3-CH_2-$\underline{C}$≡CH), 70.4 (N3-CH_2-C≡$\underline{CH}$), 65.8 (N8-CH_2), 55.4 (OCH_3), 48.0 (C-7), 44.0 (C-9), 43.0 (C-6), 38.8 (N1-CH_2), 30.3 (N8-CH_2-$\underline{CH_2}$), 30.3 (N3-CH_2), 13.4 (N1-CH_2–$\underline{CH_3}$).

1-Ethyl-8-(3-methoxyphenethyl)-3-propargyl-6,7,8,9-tetrahydropyrazino[2,1-*f*]purine-2,4(1*H*,3*H*)-dione (20j)

^{13}C-NMR ($CDCl_3$) δ 159.7 (C-3, phenyl), 153.9 (C-9a), 150.3 (C-4), 148.4 (C-2), 148.4 (C-10a), 140.9 (C-1, phenyl), 129.5 (C-5, phenyl), 121.0 (C-6, phenyl), 111.6 (C-4, phenyl), 111.4 (C-2, phenyl), 106.6 (C-4a), 78.8 (N3-CH_2-$\underline{C}$≡CH), 70.3 (N3-CH_2-C≡$\underline{CH}$), 59.0 (N8-CH_2), 55.2 (OCH_3), 51.4 (C-7), 49.1 (C-9), 44.3 (C-6), 38.8 (N1-CH_2), 33.7 (N8-CH_2-$\underline{CH_2}$), 30.3 (N3-CH_2), 13.4 (N1-CH_2–$\underline{CH_3}$).

1-Ethyl-8-(4-methoxyphenethyl)-3-propargyl-6,7,8,9-tetrahydropyrazino[2,1-*f*]purine-2,4(1*H*,3*H*)-dione (20k)

^{13}C-NMR ($CDCl_3$) δ 161.6 (C-4, phenyl), 153.9 (C-9a), 150.1 (C-4), 148.4 (C-2), 144.1 (C-10a), 129.6 (C-2 and C-6, phenyl), 128.5 (C-1, phenyl), 114.4 (C-3 and C-5, phenyl), 106.9 (C-4a), 78.5 (N3-CH_2-$\underline{C}$≡CH), 70.6 (N3-CH_2-C≡$\underline{CH}$), 58.3 (N8-CH_2), 55.3 (OCH_3), 49.7 (C-7), 48.3 (C-9), 41.9 (C-6), 39.0 (N1-CH_2), 31.0 (N8-CH_2-$\underline{CH_2}$), 30.4 (N3-CH_2), 13.1 (N1-CH_2–$\underline{CH_3}$).

1-Ethyl-8-(2,3-dimethoxyphenethyl)-3-propargyl-6,7,8,9-tetrahydropyrazino[2,1-*f*]purine-2,4(1*H*,3*H*)-dione (20l)

^{13}C-NMR ($CDCl_3$) δ 153.8 (C-9a), 150.0 (C-4), 149.4 (C-2), 148.4 (C-3 and C-4, phenyl), 142.5 (C-10a), 128.1 (C-1, phenyl), 120.6 (C-6, phenyl), 111.7 (C-5, phenyl), 111.6 (C-2, phenyl), 107.0 (C-4a), 78.4 (N3-CH_2-<u>C</u>≡CH), 70.7 (N3-CH_2-C≡<u>CH</u>), 58.0 (N8-CH_2), 55.9 (2 × OCH_3), 49.2 (C-7), 48.3 (C-9), 41.2 (C-6), 39.1 (N1-CH_2), 30.9 (N8-CH_2-<u>CH_2</u>), 30.5 (N3-CH_2), 13.3 (N1-CH_2–<u>CH_3</u>).

```
SP|P25099|AA1R_RAT    MPPYISAFQAAYIGIEVLIALVSVPGNVLVIWAVKVNQALRDATFCFIVSLAVADVAVGA 60
SP|P30543|AA2AR_RAT   ------MGSSVYITVELAIAVLAILGNVLVCWAVWINSNLQNVTNFFVVSLAAADIAVGV 54
SP|P30542|AA1R_HUMAN  MPPSISAFQAAYIGIEVLIALVSVPGNVLVIWAVKVNQALRDATFCFIVSLAVADVAVGA 60
SP|P29274|AA2AR_HUMAN MPI---MGSSVYITVELAIAVLAILGNVLVCWAVWLNSNLQNVTNYFVVSLAAADIAVGV 57
                              .:.** :*: **:::: ***** *** :*. *::.*  *:****.**:***.

SP|P25099|AA1R_RAT    LVIPLAILINIGPQTYFHTCLMVACPVLILTQSSILALLAIAVDRYLRVKIPLRYKTVVT 120
SP|P30543|AA2AR_RAT   LAIPFAITISTGFCAACHGCLFFACFVLVLTQSSIFSLLAIAIDRYIAIRIPLRYNGLVT 114
SP|P30542|AA1R_HUMAN  LVIPLAILINIGPQTYFHTCLMVACPVLILTQSSILALLAIAVDRYLRVKIPLRYKMVVT 120
SP|P29274|AA2AR_HUMAN LAIPFAITISTGFCAACHGCLFIACFVLVLTQSSIFSLLAIAIDRYIAIRIPLRYNGLVT 117
                      *.**:** *. *  :  * **:.** **:******::*****:***: ::*****: :**

SP|P25099|AA1R_RAT    QRRAAVAIAGCWILSLVVGLTPMFGWNNLSVVEQDWRANGSVGEPVIKCEFEKVISMEYM 180
SP|P30543|AA2AR_RAT   GVRAKGIIAICWVLSFAIGLTPMLGWNNCSQKD--GNSTKTCGEGRVTCLFEDVVPMNYM 172
SP|P30542|AA1R_HUMAN  PRRAAVAIAGCWILSFVVGLTPMFGWNNLSAVERAWAANGSMGEPVIKCEFEKVISMEYM 180
SP|P29274|AA2AR_HUMAN GTRAKGIIAICWVLSFAIGLTPMLGWNNCGQPKEGKNHSQGCGEGQVACLFEDVVPMNYM 177
                        **   ** **:**:.:*****:**** .  .     .   **  : * **.*: *:**

SP|P25099|AA1R_RAT    VYFNFFVWVLPPLLLMVLIYLEVFYLIRKQLNKKVSASS--GDPQKYYGKELKIAKSLAL 238
SP|P30543|AA2AR_RAT   VYYNFFAFVLLPLLLMLAIYLRIFLAARRQLKQMESQPLPGERTRSTLQKEVHAAKSLAI 232
SP|P30542|AA1R_HUMAN  VYFNFFVWVLPPLLLMVLIYLEVFYLIRKQLNKKVSASS--GDPQKYYGKELKIAKSLAL 238
SP|P29274|AA2AR_HUMAN VYFNFFACVLVPLLLMLGVYLRIFLAARRQLKQMESQPLPGERARSTLQKEVHAAKSLAI 237
                      **:***. ** *****: :**.:*   *:**::  *        :.   **:: *****:

SP|P25099|AA1R_RAT    ILFLFALSWLPLHILNCITLFCPTCQ-KPSILIYIAIFLTHGNSAMNPIVYAFRIHKFRV 297
SP|P30543|AA2AR_RAT   IVGLFALCWLPLHIINCFTFFCSTCRHAPPWLMYLAIILSHSNSVVNPFIYAYRIREFRQ 292
SP|P30542|AA1R_HUMAN  ILFLFALSWLPLHILNCITLFCPSC-HKPSILTYIAIFLTHGNSAMNPIVYAFRIQKFRV 297
SP|P29274|AA2AR_HUMAN IVGLFALCWLPLHIINCFTFFCPDCSHAPLWLMYLAIVLSHTNSVVNPFIYAYRIREFRQ 297
                      *: ****.******:**:*:**  * : *  * *:**.*:* **.:**::**:**::**

SP|P25099|AA1R_RAT    TFLKIWNDHFRCQPKPPIDEDLPEEK-----AED------------------------- 326
SP|P30543|AA2AR_RAT   TFRKIIRTHVLRRQEPFQAGGSSAWALAAHSTEGEQVSLRLNGHPLGVWANGSATHSGRR 352
SP|P30542|AA1R_HUMAN  TFLKIWNDHFRCQPAPPIDEDLPEER-----PDD------------------------- 326
SP|P29274|AA2AR_HUMAN TFRKIIRSHVLRQQEPFKAAGTSARVLAAHGSDGEQVSLRLNGHPPGVWANGSAPHPERR 357
                      ** ** . *.  :  *    .           :.

SP|P25099|AA1R_RAT    ------------------------------------------------------------
SP|P30543|AA2AR_RAT   PNGYTLGLGGGGSAQGSPRDVELPTQER------QEGQEHPGLRGHLVQARVGASSWSSE 406
SP|P30542|AA1R_HUMAN  ------------------------------------------------------------
SP|P29274|AA2AR_HUMAN PNGYALGLVSGGSAQESQGNTGLPDVELLSHELKGVCPEPPGLDDPLAQDGAGVS----- 412


SP|P25099|AA1R_RAT    ----
SP|P30543|AA2AR_RAT   FAPS 410
SP|P30542|AA1R_HUMAN  ----
SP|P29274|AA2AR_HUMAN ----
```

Figure S1. Sequence alignment of human and rat A_1 and A_{2A} adenosine receptor subtypes. Important residues in the binding pocket are highlighted in yellow.

Table S1. Solubility of selected compounds at three different pH values (in mg/mL).[a]

**Compound**	pH 1	pH 4	pH 7.4
**14a**	**0.2**	0.0009	0.0008
**14b**	**0.1**	0.001	0.0009
**14c**	**1.3**	0.006	0.003
**14d**	**0.2**	0.001	<0.0005
**14e**	**0.4**	0.002	0.002
**14g**	**>1.5**	**0.05**	**0.04**
**15f**	**0.3**	0.0009	0.0007
**15g**	**0.6**	0.002	0.002

[a]determined by thermodynamic solubility measurements.
